# Systemic Activation of the Antioxidant System by Root Priming With Non‐Pathogenic *Fusarium oxysporum* in Flax Infected With Pathogenic *Fusarium oxysporum*

**DOI:** 10.1111/1758-2229.70263

**Published:** 2026-01-08

**Authors:** Marta Burgberger, Justyna Mierziak, Wioleta Wojtasik

**Affiliations:** ^1^ Department of Genetic Biochemistry, Faculty of Biotechnology Wroclaw University Wroclaw Poland

**Keywords:** flax, non‐pathogenic *Fusarium oxysporum*, pathogenic *Fusarium oxysporum*, priming, ROS metabolism, systemic response

## Abstract

Plants rely on specialised adaptive mechanisms to enhance resistance against environmental stress. One such mechanism, priming, enables faster and stronger defence responses upon subsequent stress exposure. This study examines whether the non‐pathogenic *Fusarium oxysporum* Fo47 primes flax by colonising roots and activating antioxidant defences. Flax plants primed with Fo47 and those treated with both Fo47 and the pathogenic strain *F. oxysporum* Foln were analysed for fungal colonisation, *PR genes* expression and antioxidant systems: enzymatic (ROS metabolism‐related genes expression, catalase and superoxide dismutase activity, hydrogen peroxide and superoxide anion levels) and non‐enzymatic (phenolic compound content and antioxidant potential). The results demonstrate that Fo47 colonises host tissues, significantly reducing Foln penetration and colonisation, particularly in primed plants. Root‐specific suppression of Foln by Fo47 was stronger than systemic suppression in shoots. Fo47 induced early *chitinase* and *NADPH oxidases D* transcript accumulation and reduced superoxide anion level in roots, likely triggering defence activation. Notably, Fo47 also activated both enzymatic and non‐enzymatic antioxidant systems in shoots, suggesting a systemic priming effect. These findings underscore the potential of non‐pathogenic *F. oxysporum* strains in sustainable plant protection strategies.

## Graphical abstract

*Fusarium oxysporum* Fo47 primes flax by locally suppressing pathogens in roots and systemically activating antioxidant defences in shoots, offering a sustainable plant protection strategy.

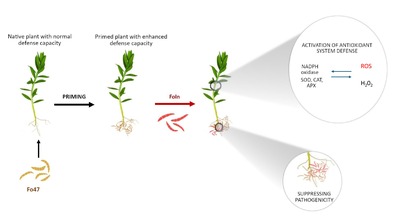

## 1 Introduction

In their natural environment, plants must constantly defend themselves against abiotic and biotic stress factors, including pathogen invasion. *Fusarium oxysporum* is one of the most important plant pathogens with a direct impact on the global economy and food safety (Dean et al. 2012). This soilborne asexual fungus is known to include both pathogenic and non‐pathogenic strains. Typical symptoms of infection with pathogenic strains include stunting, wilting, chlorosis, necrosis and damage to vascular bundles. They are mainly the result of colonisation of plant vascular xylem tissue by this group of pathogens (Gordon 2017; Martínez‐Soto et al. 2023). *F. oxysporum* f. sp. *lini* (Foln) is one of the main pathogens attacking flax crops. It causes Fusarium wilt disease of flax, which leads to a significant decrease in yield and fibre quality (Planchon et al. 2021). *F. oxysporum* is capable of synthesising various mycotoxins, including beauvericin, enniatins, fusaric acid, moniliformin, naphthazarins, sambutoxin, and, under certain conditions, also type A trichothecenes. However, the production of these secondary metabolites is strongly dependent on the strain and environmental conditions. The presence of these toxins may affect both the pathogenicity of the fungus and the safety of crop plants and food products (Mirocha et al. 1989; Magan 2007; Li et al. 2013).

Root colonisation by an endophytic non‐pathogenic strain of *F. oxysporum* may reduce the symptoms of disease caused by pathogenic strains. One of the first *F. oxysporum* endophytes that has been shown to reduce the risk of developing Fusarium blight is the *F. oxysporum* Fo47 strain (Alabouvette et al. 1987). Endophytic Fo47 reduces Fusarium wilt in various crop species such as tomato, asparagus, chickpeas, and cotton (Trouvelot et al. 2002; He and Wolyn 2005; Kaur and Singh 2007; Zhang et al. 2018; Constantin et al. 2019). Such observations were also obtained in the case of flax. Duijiff and co‐authors showed that the use of the Fo47 strain allows to limit the development of Fusarium wilt in flax (Duijff et al. 1999). However, it is unclear how endophyte‐mediated Fusarium resistance is achieved, and the literature describes different mechanisms depending on the host. Under stressful conditions, plant cells are able to produce large amounts of reactive oxygen species (ROS), which consist mainly of H_2_O_2_. Research by Olivain and co‐authors indicates that both pathogenic and non‐pathogenic strains of *F. oxysporum* may trigger transient H_2_O_2_ production during flax colonisation (Olivain et al. 2003).

Plant cells produce ROS, such as hydrogen peroxide (H_2_O_2_), singlet oxygen (^1^O_2_), superoxide anion (O_2_^•−^), hydroxyl radical (•OH), as byproducts of their normal metabolism. ROS can act as toxic molecules, using this property as a weapon against pathogens, but ROS can also act as important signalling molecules in the defence response (Camejo et al. 2016; Wang et al. 2019; Sood 2025). Cell death caused by ROS can lead to necrosis of host cells from which pathogens take nutrients and thus limit the development of infection (Tian et al. 2016). The plant's early defensive reaction to a pathogen attack is termed oxidative burst that generates local and transient ROS production (Camejo et al. 2016). In addition to this direct response to the presence of a pathogen, ROS are also involved in cell signalling related to the induction of the expression of defence genes (e.g., *pathogenesis‐related 1, 2, 3, 4*, *glutathione S‐transferase*, *ascorbate peroxidase*, *catalase*, *superoxide dismutase*, *phenylalanine ammonia‐lyase*, *glucan synthase–like5, transcription factors WRKY, NPR1 regulator)* and interface with other signalling molecules, changes in the cell wall, hypersensitivity reaction (HR), callose deposition and systemic acquired immunity (SAR) (O'Brien et al. 2012). ROS production in response to biotic stress is biphasic: the first phase usually occurs within minutes of pathogen attack, but is transient and weak, while the second phase is much more intense and persistent (Camejo et al. 2016). However, ROS accumulation must be under the control of antioxidant systems to avoid excessive toxicity. The activity of antioxidant enzymes such as ascorbate peroxidase (APX), superoxide dismutase (SOD), NADPH oxidase (NADPHox) and catalase (CAT) plays an important role in these processes (Blokhina et al. 2003; Apel and Hirt 2004). One of the primary systems for removing H_2_O_2_ in plant cells is the ascorbate–glutathione cycle. Within this pathway, APX uses ascorbate as a dedicated electron donor to reduce H_2_O_2_ to water. In higher plants, different APX isoforms are present in various subcellular locations, such as chloroplasts, mitochondria, peroxisomes, and the cytosol, allowing fine control of H_2_O_2_ concentrations at both the organelle and whole cell level. APX is likely also to act as a H_2_O_2_ signalling regulator. In addition to using ascorbate, APXs have recently been shown to accept a wider range of substrates and to exhibit chaperone‐like activity, enabling them to contribute to diverse biological functions (Li 2023). SOD is an enzyme that facilitates the dismutation of the superoxide radical into molecular oxygen (O_2_) and hydrogen peroxide. As a metalloenzyme, SOD relies on bound metal ions to carry out this conversion. In plant cells, SODs Cu/Zn are typically present in the cytosol, chloroplasts, and sometimes in the apoplast; SODs Fe are localised within chloroplasts, while SODs Mn are mainly found in the mitochondrial matrix and in peroxisomes. SOD serves as the primary defence against oxidative damage and is crucial for regulating ROS generated under both abiotic and biotic stress conditions. Increased transcript abundance of SODs is observed in response to stress (Racchi 2013; Kunos et al. 2022). Plant immunity comprises tiers of pathogen recognition in which NADPH oxidases constitute central generators of ROS that initiate and shape defence signalling, and thereby influence resistance across multiple plant systems (*Arabidopsis* *thaliana*, tobacco, potato, rice, and barley). They are the key enzymes in generation of O•_2_^−^ by catalysing the transfer of electrons from NADPH to O_2_ (Hu et al. 2020; Zhu et al. 2024). Catalase is an iron‐containing protein composed of four identical subunits. The enzyme is widely present in peroxisomes, where it converts hydrogen peroxide into water and oxygen. Together with SOD and hydroxyperoxidases, catalase forms part of the antioxidant defence system that eliminates superoxide radicals. In most cases, CAT (because of its high KM) removes the majority of H_2_O_2_ that is transported to peroxisomes, while enzymes such as APX, which has a lower KM and thus a higher affinity for H_2_O_2_ and is distributed across various subcellular compartments, precisely regulates the levels of this reactive molecule. It is regarded as one of the primary antioxidant enzymes, playing a crucial role during both development and stress responses (Palma et al. 2020; Baker et al. 2023). Also, plants possess non‐enzymatic defence systems composed of naturally occurring antioxidant molecules. These include water‐soluble substances such as ascorbate, glutathione, phenolic compounds, and flavonoids, as well as lipid‐soluble metabolites like carotenoids and α‐tocopherols. These antioxidants neutralise ROS by donating electrons, converting them into less harmful forms, while the oxidised byproducts generated in this reaction remain relatively stable and non‐toxic (Rudenko et al. 2023). Disturbances in redox homeostasis, caused by changed levels of ROS or the efficiency of antioxidant systems, may play a key role in the defensive reactions of plant tissues to pathogens; therefore, in this work, we want to investigate whether this mechanism also plays a role during flax resistance to Fusarium wilt obtained through the non‐pathogenic strain *F. oxysporum*.

In order to investigate the protective effect of the non‐pathogenic strain *F. oxysporum* (Fo47) on the resistance of flax to the pathogenic strain *F. oxysporum* (Foln), the plants were treated with Fo47 and then after 4 days exposed to Foln. To check whether Fo47 can have a protective effect on flax during simultaneous infection with a pathogenic strain, the flax was treated with both strains simultaneously. In both cases, the control plants were plants not treated with any of the fungal strains and plants treated with Fo47 or Foln. The objective of this study was to determine whether priming with the non‐pathogenic strain Fo47 can activate the antioxidant system of flax and thereby enhance its resistance to the pathogenic strain Foln. We hypothesised that Fo47 treatment would systemically stimulate antioxidant defences, leading to reduced disease symptoms and improved plant performance.

## 2 Materials and Methods

### 2.1 Biological Material and Growth Conditions

Flax seeds ( *Linum usitatissimum* L., cv. *Nike*) were obtained from the Flax and Hemp Collection of the Institute of Natural Fibres and Medicinal Plants in Poland. Flax seeds were sterilised with 70% ethanol for 3 min and in 50% PPM (Plant Preservative Mixture; Plant Cell Technology, UK) for 10 min. Subsequently, seeds were sown on vermiculite supplemented with Murashige and Skoog medium (MS medium; Sigma‐Aldrich). Seedling growth was conducted for 14 days under a controlled environment in a plant growth chamber, maintaining a 16‐h light (21°C) and 8‐h darkness (16°C) regime, light intensity: 23 mmol/s/m^3^, and they were subsequently used for further experiments.

For the experiments, the non‐pathogenic strain of *F. oxysporum* (Fo47) (ATCC Number: MYA‐1198) and the pathogenic strain *F. oxysporum* f.sp. *lini* (Bolley) Snyder et Hansen (Foln) (ATCC MYA‐1201) were acquired from ATCC (USA). The *Fusarium* fungi were cultured on Petri dishes with PDA medium at 28°C. Spores, comprising a mix of macro‐ and microconidia, were harvested after flooding the plate with 3 mL of sterile water. Spore count was performed using a haemocytometer. Spore suspensions were diluted to 10^6^ spores/ml to execute the inoculations.

### 2.2 Flax Inoculation With *Fusarium oxysporum* Strains

**FIGURE 1:**
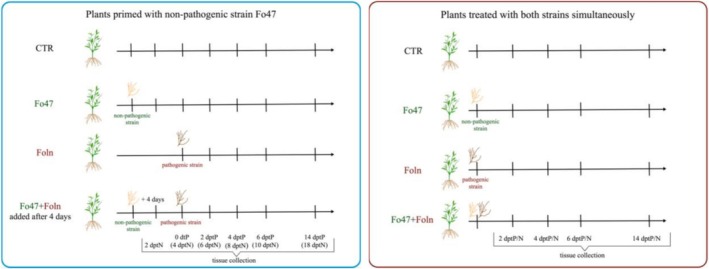
Two research models. Flax plants primed with the non‐pathogenic strain of *Fusarium oxysporum* and then after 4 days treated with the pathogenic strain of *Fusarium oxysporum*—blue frame. Flax plants treated with both the non‐pathogenic strain of *F. oxysporum* and the pathogenic strain of *F. oxysporum* simultaneously—red frame. Ctr—non‐treated plants; Fo47—plants treated with the non‐pathogenic strain of *F. oxysporum* alone; Foln—plants treated with the pathogenic strain of *F. oxysporum* alone; Fo47 + Foln—plants primed with non‐pathogenic strain of *F. oxysporum* or plants treated with both strains of *F. oxysporum* simultaneously.

Two in vitro research models were conducted (Figure 1). Model 1: Two‐week‐old flax plants were treated with 0.5 mL of the non‐pathogenic strain at a concentration of 10^6^ spores/ml, and after 4 days with the pathogenic strain at the same concentration. Model 2: Two‐week‐old flax plants were treated simultaneously with 0.5 mL of the non‐pathogenic and pathogenic strain, both at a concentration of 10^6^ spores/ml. Appropriate controls were conducted, consisting of plants untreated with any fungal strain, plants treated solely with the pathogenic strain, or solely with the non‐pathogenic strain at appropriate time points. Roots and shoots were harvested separately. In model 1, samples were collected at the following time points: 2 dptN (2 days post treatment with Fo47), 4 dptN/0 dptP (4 days post treatment with Fo47/0 days post treatment with Foln), 6 dptN/2 dptP, 8 dptN/4 dptP, 10 dptN/6 dptP, and 8 dptN/14 dptP. In model 2, samples were collected at 2, 4, 6, and 14 days post treatment with the Foln and Fo47 strains (dptP/N). All harvested material was immediately frozen in liquid nitrogen and stored at −80°C until further analysis. The experiment was conducted in three biological replicates.

Phenotypic changes illustrating disease symptoms (yellowing of leaves, brown spots on leaves, wilting, necrosis, and browning of roots) were visually assessed and photographed after 28 days, and again after 42 days.

### 2.3 Microscopic Observation of the Progress of Fungal Infection

Whole plants were collected and treated with 0.15% TCA in ethanol: chloroform mixture (4:1, v/v) for 48 h. Then the roots were subjected to a series of rinses: 15 min in sterile water, 15 min in 50% ethanol—twice, 15 min in 50 mM NaOH—twice, three times in sterile water, 30 min in 0.1 M Tris–HCl buffer, pH = 8.5. After a series of washes, the tissue was stained. The roots were immersed in a safranin solution (0.2% w/v safranin in 10% v/v ethanol) for 3 min and washed three times with sterile water for 10 min each. The tested material was cut into sections using a razor blade and placed on microscope slides. Then, sections on slides were stained for 10 min with solophenylflavin 7GFE (0.1% w/v in 0.1 M Tris/HCl, pH 8.5) and washed again 3 times with sterile water. Microscopic preparations were observed under a ZEISS AXIO Scope A.1 fluorescence microscope (excitation wavelength 360–370 nm) and documented using software and a black and white AxioCam ICm1 camera at 40× magnification. After taking the photos, the colours were digitally applied to improve visualisation. Based on microscopic images, we created a 5‐point scale of the number of *F. oxysporum* hyphae in flax tissues, where: 0—clear, 1—single fungal hyphae, 2—few fungal hyphae, 3—numerous fungal hyphae and 4—very numerous fungal hyphae.

### 2.4 Disease Index (DI) and Symptom Assessment

Disease symptoms were evaluated at 28 and 42 days after treatment with the pathogenic strain *F. oxysporum* f. sp. *lini*. The assessment of symptom severity and calculation of the disease index were conducted following the methodology described by Attia et al. (2022). Symptom severity was classified into five distinct categories: (1) no visible symptoms; (2) slight yellowing of the lower leaves with minor wilting; (3) significant yellowing extending to the upper parts of the plant, accompanied by wilting; (4) severe wilting along with leaf discoloration, including necrosis, chlorosis, yellowing, and browning; (5) complete plant death.

The Disease Index (DI) was determined using a five‐level scale, applying the formula: DI% = ((0*n0) + (1*n1) + (2*n2) + (3*n3) + (4*n4))*100/nt*4, where n0‐n4 represent the number of plants assigned to each symptom category, nt denotes the total number of tested plants, and 4 signifies the highest severity level in the classification system.

### 2.5 Isolation of Plant and Fungal DNA

Isolation of both plant and fungal DNA was performed using CTAB (cetyl trimethylammonium bromide) buffer. 100 mg of the research material was ground in liquid nitrogen, 700 μL of CTAB buffer (100 mM Tris–HCl pH 8.0, 20 mM EDTA, 1.4 M NaCl, 1% β‐mercaptoethanol, 2 mg PVPP) was added, mixed and incubated in temp. 65°C for 30 min with shaking. The samples were centrifuged (10 min, 18,000×*g*); 450 μL of a mixture of chloroform:isoamyl alcohol (24:1) was added to the supernatant, mixed and centrifuged (8 min, 18,000×*g*). The aqueous phase was collected and 1 volume of isopropanol and 30 μL of 3 M NaOAc pH = 5.2 were added to it, mixed and left for 15 min at −20°C. The samples were centrifuged for 10 min, 18,000×*g*. The precipitate was washed with 70% ethanol. After ethanol removal, the DNA pellet was suspended in 40 μL of water with RNase (0.5 μg/μL) and incubated for 10 min at 65°C. DNA integrity was examined by gel electrophoresis on 1.0% (w/v) agarose, and the amount of DNA was determined spectrophotometrically.

### 2.6 Identification of Fungal DNA in Plants

The isolated DNA was used as a template for the PCR reaction, in which fragments of the Fo47 and Foln genomes were amplified. The distinction was possible due to the presence of a 68‐bp deletion in the propagated fragment of the Fo47 genome. A fragment of 152 bp for Fo47 and 220 bp for Foln was amplified. The sequences of primers are presented in Table S1. Program: 95°C, 3:00 min; 95°C, 0:30 min; 59°C, 0:30 min; 72°C, 0:05 min; 72°C, 5:00 min. PCR products were subjected to electrophoretic separation in a 2% agarose gel and at voltages adjusted to the size of the separated DNA in TBE buffer (90 mM Tris, 90 mM boric acid, 20 mM EDTA). The separated DNA was visualised under UV light.

### 2.7 Determination of the Relative Amount of Fungal DNA in Plants

A qPCR reaction was performed on DNA templates to amplify a fragment of the fungal gene encoding murein transglycosylase found in the genome of both *Fusarium* strains. Isolated DNA previously diluted to a final concentration of 100 ng/μL was used as the template for the reaction. The reaction was carried out using the StepOnePlus Real‐Time PCR System by Applied Biosystems. The sequences of primers are presented in Table S1.

### 2.8 Determination of the Amount of DNA of the Pathogenic *F. oxysporum* Strain in Plants

The amount of *F. oxysporum* DNA was determined by real‐time PCR using the AmpliTest *F. oxysporum* (pathogenic) kit (Amplicon, Poland) in accordance with the protocol provided by the manufacturer. A fragment of the *six 7* (*secreted in xylem 7*) gene of *F. oxysporum* of the pathogenic strain was amplified. Previously isolated DNA diluted to a final concentration of 100 ng/μl was used as a template for the reaction. The reactions were performed using the StepOnePlus Real‐Time PCR System thermal cycler from Applied Biosystems.

### 2.9 Isolation of RNA From Plant Material

TRIzol reagent (Thermo Fisher Scientific, USA) was used to isolate total RNA from shoots. Isolation was carried out according to the procedure provided by the manufacturer. Isolation of RNA from roots was performed using the Spectrum Plant Total RNA Kit (Sigma Aldrich, USA) according to the attached procedure. The concentration of the obtained RNA was determined by spectrophotometric measurement at a wavelength of 260 nm, and the purity was assessed based on the A260/A280 and A260/A230 ratios. Moreover, the integrity and quality of the isolated RNA were checked by electrophoretic separation under denaturing conditions. In order to get rid of the remains of genomic DNA, a digestion reaction was performed using DNase I (Thermo Fisher Scientific, USA) according to the procedure provided by the manufacturer.

### 2.10 The mRNA Levels Analysis

The mRNA level of the tested genes was determined using qPCR. The reaction template was cDNA. The Hight Capacity cDNA Reverse Transcription Kit (Thermo Fisher Scientific, USA) was used to synthesise cDNA on an RNA template. The reaction was carried out according to the procedure provided by the manufacturer. Real‐time PCR reactions were performed using the SYBR SG qPCR Master Mix kit from EURX, Poland in the StepOnePlus Real‐Time PCR System thermal cycler from Applied Biosystems. The reaction conditions were designed in accordance with the instructions provided by the kit manufacturer. Primers were designed using LightCycler Probe Design Software 2, and their specificity was confirmed by analysing PCR products using the melting curve method. The annealing temperature of the primers was 57°C, and their sequences are presented in Table S1. Each reaction was performed in triplicate. The actin gene was used as a reference gene for data normalisation. Changes in mRNA levels are presented as x‐fold relative amounts (RQ) standardised to actin compared to mRNA levels in control plants.

### 2.11 Superoxide Dismutase Activity

A 50 mg of ground in liquid nitrogen tissue was extracted in 500 μL of cold 0.1 M Tris–HCl buffer, pH 7.4, containing 0.5% Triton X‐100, 5 mM β‐mercaptoethanol, and protease inhibitors. The samples were centrifuged (14,000×*g*) for 15 min at 4°C, and the lysate was transferred to a new tube and used to determine enzyme activity using the Superoxide Dismutase (SOD) Activity Assay Kit (Sigma‐Aldrich) according to the procedure provided by the manufacturer. SOD activity was expressed in U/mL/mg protein (calculated based on the Bradford Method with BSA as a standard).

### 2.12 Catalase Activity

A 50 mg of ground in liquid nitrogen tissue was extracted in 0.5 mL of cold extraction buffer (0.1 M phosphate buffer pH = 7.5, 1 mM PMSF, 1 mM DTT, 2% PVPP, 0.1% EDTA, 1% Triton X‐100), and then placed on ice and disintegrated using a sonicator. The sonication process was carried out in 3 cycles of 15 s of disintegration and 10 s of break at an amplitude of 50%. Then, the samples were centrifuged at 15,000×*g* for 15 min at 4°C, and the protein concentration of the obtained lysates was determined according to the Bradford Method with BSA as a standard. The extracts obtained were used to determine catalase activity. 1.980 mL or 1.850 mL of phosphate buffer (1 M, pH 7.5) and 1 mL of 0.2% aqueous H_2_O_2_ solution were mixed in the reaction tube; 0.02 mL of plant extract from shoots or 0.15 mL from roots was added to the mixture, vortexed, and immediately transferred to a measuring cuvette. Absorbance was measured at a wavelength of 240 nm for 5 min, taking readings every 1 min against a blank. Catalase activity was determined based on the decrease in absorbance over time and the molar extinction coefficient of 39.4 mM^−1^ cm^−1^ (Aebi 1984).

### 2.13 Determination of Hydrogen Peroxide Content

A 50 mg of powdered sample was extracted in 200 μL of 20 mM phosphate buffer (pH 6.5) and then centrifuged at 10,000×*g* for 10 min at 4°C. The obtained supernatant was transferred into a clean microcentrifuge tube, and 50 μL of this extract was used for hydrogen peroxide quantification. Measurements were carried out with the Amplex Red Hydrogen Peroxide/Peroxidase Assay Kit (Life Technologies) according to the manufacturer's protocol. Detection of the signal was performed using a Varioskan Flash reader (Thermo Scientific).

### 2.14 Determination of Antioxidant Potential Using DPPH

A 100 mg of shoots or roots were ground in liquid nitrogen. Samples were extracted three times with 1 mL of methanol, centrifuging after each extraction. The resulting supernatants were combined and evaporated to dryness using a vacuum concentrator, then resuspended in 0.5 mL of methanol. The pellet remaining after methanol extraction was treated with 1 mL of 2 M NaOH and subjected to alkaline hydrolysis for 24 h at 37°C. Following hydrolysis, the samples were centrifuged, the supernatant was adjusted to pH 3 and extracted three times with 1 mL of ethyl acetate. The combined organic fractions were evaporated, and the residue was dissolved in 0.5 mL of methanol. Both methanolic (containing free phenolics) and ethyl acetate (containing bound phenolics) extracts were used for the determination of antioxidant activity with the DPPH assay. On a 96‐well microplate, 200 μL of 0.1 mM DPPH solution in methanol was added to each well, followed by 6 μL of sample. The mixture was incubated for 15 min in the dark, and absorbance was measured at 515 nm using a microplate reader. Controls included DPPH with methanol (control sample) and methanol only (blank).

### 2.15 Superoxide Radical Staining

The sufficient amount of NBT (nitroblue tetrazolium) staining solution (3.5 mg/mL) was prepared in potassium phosphate buffer (10 mM) with the addition of 10 mM NaN_3_. The plants were immersed and infiltrated under vacuum for 1 h. Previously stained plants were bleached in acetic acid‐glycerol‐ethanol (1/1/3) (v/v/v) solution at 100°C for 5 min. Plants were stored in glycerol‐ethanol (1/4) (v/v) solution. The NBT‐stained plants were grounded in liquid nitrogen. The powder was solubilised in 2 M KOH‐DMSO (1/1.16) (v/v). Probes were centrifuged for 10 min at 12,000×*g* at 4°C. The absorbance at 630 nm was measured. A standard curve was prepared using known amounts of NBT in the KOH‐DMSO ranging from 0 to 25 nmol.

### 2.16 Determination of Phenolic Compounds by UPLC

Phenylpropanoid compounds were extracted from 200 mg of shoots or roots using methanol. Samples were extracted three times with 1 mL methanol, centrifuged, and the combined supernatants were evaporated to dryness under reduced pressure and re‐dissolved in 0.5 mL methanol. Cell wall–bound phenylpropanoids were obtained from the residual pellet by alkaline hydrolysis (1 mL of 2 M NaOH, 24 h, 37°C). After centrifugation, the supernatant was acidified to pH 3 with HCl and extracted three times with ethyl acetate. The combined organic fractions were evaporated and re‐dissolved in 0.5 mL methanol. Extracts were analysed using an Acquity UPLC system (Waters, Milford, MA, USA) equipped with a PDA detector. Separation was performed on a BEH C18 column (2.1 × 100 mm, 1.7 μm) with a mobile phase of 0.1% formic acid in water (A) and acetonitrile (B), at a flow rate of 0.3 mL/min. The gradient program was: 0–1 min, 95% A; 2–12 min, 70% A; 11–15 min, 0% A; 17 min, 95% A. UV spectra were recorded from 210–500 nm, with quantification performed at 320 nm. Compounds were identified by retention times and spectral characteristics, and quantified using calibration curves from standards. Data acquisition and peak integration were performed using MassLynx software (Waters).

### 2.17 Statistical Analysis

In each experimental group, six jars were used, with each jar containing 16 plants. The collected plants were treated as a single pool. The experiment was conducted in three independent biological replicates. The results were presented as the mean of the three repetitions for each experiment, along with the corresponding standard deviation. Statistical analyses were carried out using Statistica 13 software (StatSoft, USA). To assess statistical significance, a one‐way ANOVA was performed, followed by Fisher's post hoc test. Differences were considered statistically significant when *p* < 0.05.

## 3 Results

### 3.1 Phenotypic Observations

**FIGURE 2:**
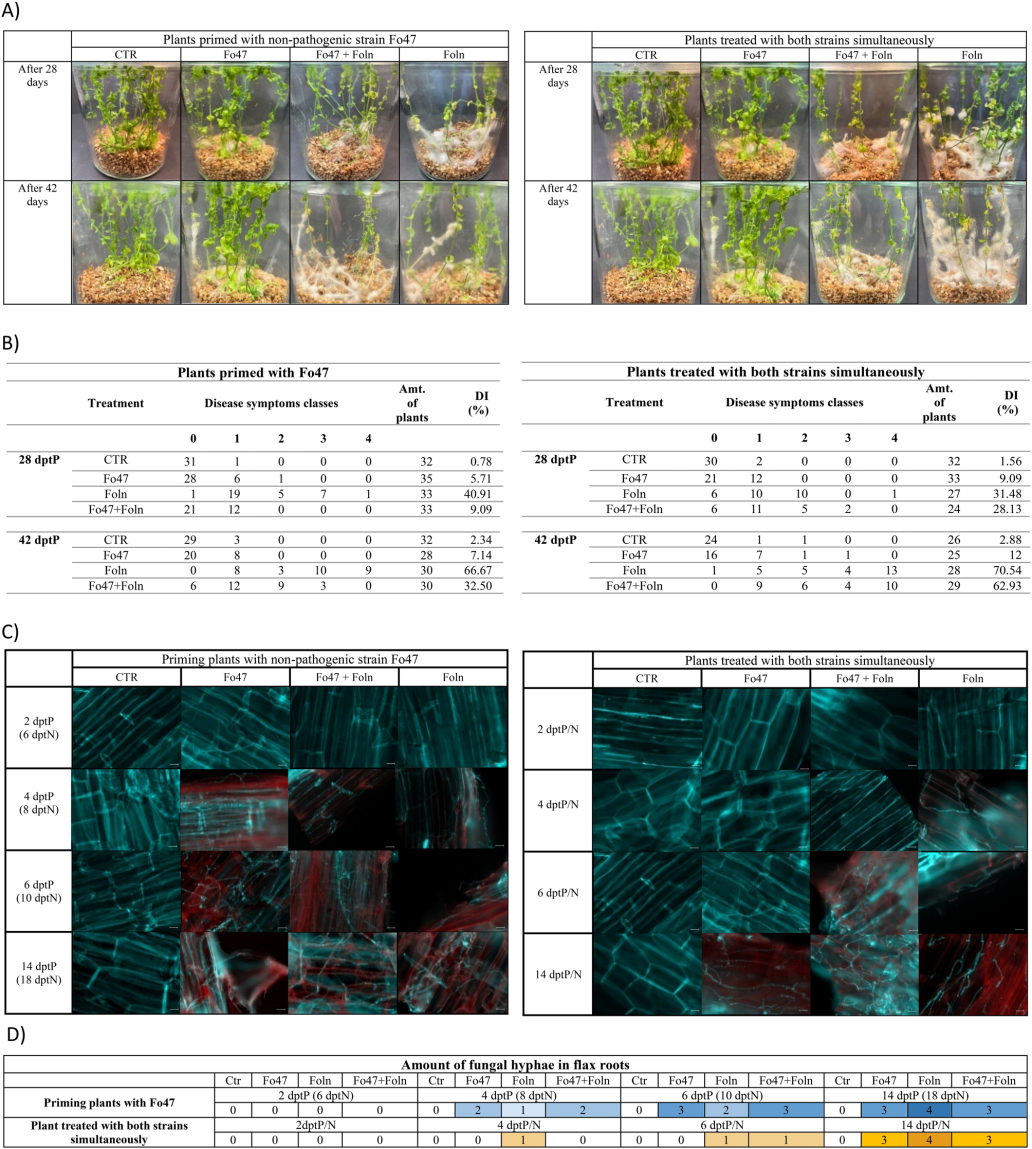
Colonisation of plants primed with non‐pathogenic strain *of Fusarium oxysporum* and plants treated with both strains *of F. oxysporum* simultaneously. (A) Phenotypic changes in plants treated with the non‐pathogenic strain of *F. oxysporum*, the pathogenic strain of *F. oxysporum*, both strains, primed with Fo47 and control plants observed at 28 and 42 days post‐treatment. (B) Disease Index—Based on the phenotypic assessment of plants from different treatment groups across three biological replicates, the number of plants falling into zero of four symptom severity categories was determined. Using this classification, the disease index was calculated (symptom severity categories and the formula for disease index calculation is included in methodology section). (C) Microscopic observations of flax roots was conducted at specific time points (dptP—days post treatment with the pathogenic strain Foln; dptN—days post treatment with the non‐pathogenic strain Fo47; dptP/N—days post treatment with both strains). Scale bar = 50 μm (D) The fungal amount in flax roots was visually assessed through microscopic observations conducted on samples from three independent biological replicates of the experiment. The number of hyphae was visually evaluated and classified into groups ranging from 0 (clear tissue) to 4 (tissue completely colonised by hyphae). 0—clear, 1—single fungal hyphae, 2—few fungal hyphae, 3—numerous fungal hyphae and 4—very numerous fungal hyphae.

In both analysed groups of plants, plants treated with the non‐pathogenic strain did not show significant phenotypic changes (Figure 2A). In the case of plants treated with the pathogenic strain, after 28 days after infection, we notice that the plants have become yellowish or brown, their leaves have curled and the stem has begun to bend, and mycelium growth is also observed above the vermiculite surface, as well as on the surface of the plants. After 42 days, these symptoms intensified. Primed plants showed signs of infection 28 and 42 days after treatment, but they were not as extensive as in the case of plants treated only with the pathogenic strain. Plants treated simultaneously with a non‐pathogenic and pathogenic strain became bent, the leaves began to curl and change colour to yellowish‐brown, and the growth of mycelium on their surface was also noticeable. However, these changes were less advanced than in the case of plants treated only with the Foln strain, but more extensive than in the case of primed plants.

Additionally, disease index (DI) analysis confirmed significant differences between the plant groups (Figure 2B). At 28 days post‐treatment (dptP), the DI for plants primed with Fo47 and after 4 days treated with Foln was 9.09%, whereas it reached 40.91% in plants treated with Foln and 5.71% in plants treated with Fo47 alone. At 42 dptP, the DI was 32.50% for plants primed with Fo47 and after 4 days treated with Foln, 66.67% for plants treated with Foln, and 7.14% for plants treated with Fo47 alone. In the group treated with both strains simultaneously, the DI values were as follows: at 28 dptP, 28.13% for plants treated with Fo47 and Foln, 31.48% for plants treated with Foln, and 9.09% for plants treated with Fo47; at 42 dptP, 62.93% for plants treated with both Fo47 and Foln, 70.54% for plants treated with Foln, and 12% for plants treated with Fo47 alone.

### 3.2 Visualisation of Fungal Hyphae in Flax Roots

The colonisation of flax roots by fungal strains is presented in microscope images (Figure 2C), based on which a five‐grade scale was established to quantify the presence of fungal hyphae in root tissues (Figure 2D). In untreated plants (Ctr), no fungal hyphae were observed in root tissues at any of the analysed time points. In plants treated exclusively with the non‐pathogenic strain Fo47, a small number of fungal hyphae were first observed at 4 dptP (8 dptN). These hyphae were distributed in various regions of the root tissue. At 6 and 14 dptP (10 and 18 dptN), the number of hyphae increased significantly, with their presence confirmed in numerous analysed cross‐sections. The hyphal abundance at these time points remained comparable. In plants treated exclusively with the pathogenic strain Foln, individual hyphae were detected at 4 dptP. At 6 dptP, a rapid increase in hyphal number was observed, with numerous hyphae present in most analysed cross‐sections. By 14 dptP, a very high density of hyphae was visible in the majority of examined samples. In primed plants, individual hyphae were observed at 4 dptP. By 6 dptP, the hyphal density had significantly increased, reaching high levels in many analysed cross‐sections. However, by 14 dptP, the number of hyphae did not increase further and remained at a level comparable to that observed at 6 dptP. In plants treated simultaneously with both Fo47 and Foln, individual fungal hyphae were detected at 6 dptP. By 14 dptP, the number of hyphae had increased and was visible in most analysed cross‐sections.

### 3.3 Presence of a Non‐Pathogenic and Pathogenic Strain of *Fusarium oxysporum* in the Tested Flax Plants

In order to confirm the presence of pathogenic and non‐pathogenic *F. oxysporum* strain in flax plants, PCR was performed on a fragment of the genome of these fungi. The amplified Fo47 genome fragment was 68 bp shorter than the Foln genome fragment, which made it possible to distinguish the resulting products on an agarose gel (Figure S1A). In the roots of plants treated with the non‐pathogenic strain, the presence of Fo47 DNA was confirmed already at 0 dptP (4 dptN), which increased over time. In plants treated with the pathogenic strain on day 2, we observe a relatively small amount of Foln DNA, but a significant amount of it is visible at 6 and 14 dptP. However, in the roots of primed plants at 2 and 4 dptP (6 and 8 dptN), only the PCR product amplified on the basis of the genome of the non‐pathogenic strain is visible. At 6 dptP, in the roots of these plants, a PCR product on the DNA template of the pathogenic strain additionally appears, the amount of which increases at 14 dptP. In the roots of the second group of plants, at 2 dptP/N only the PCR product from Fo47 is visible and it is in a small amount; however, it increases over time. In plants treated with the pathogenic strain, the PCR product is noticeable from 4 dptP. In plants treated with two strains at the same time, unlike primed plants, the visible product comes mainly from the pathogenic strain, and its amount increases with time. In the shoots of plants primed and plants treated with a non‐pathogenic strain, PCR products amplified only on the Fo47 genome are observed from 2 dptP (6 dptN), while the PCR product originating from the Foln genome is visible from 4 dptP. In plants treated with both strains simultaneously at 4, 6 and 14 dptP/N, PCR products amplified from both genomes were visible, but the amount of genomic DNA of the pathogenic strain was much greater. At 2 dptP/N, no PCR products were observed for any of the genomes, while at 4 dptP/N the amount of PCR products was small for Fo47 and Foln, but an increase over time was visible.

**FIGURE 3:**
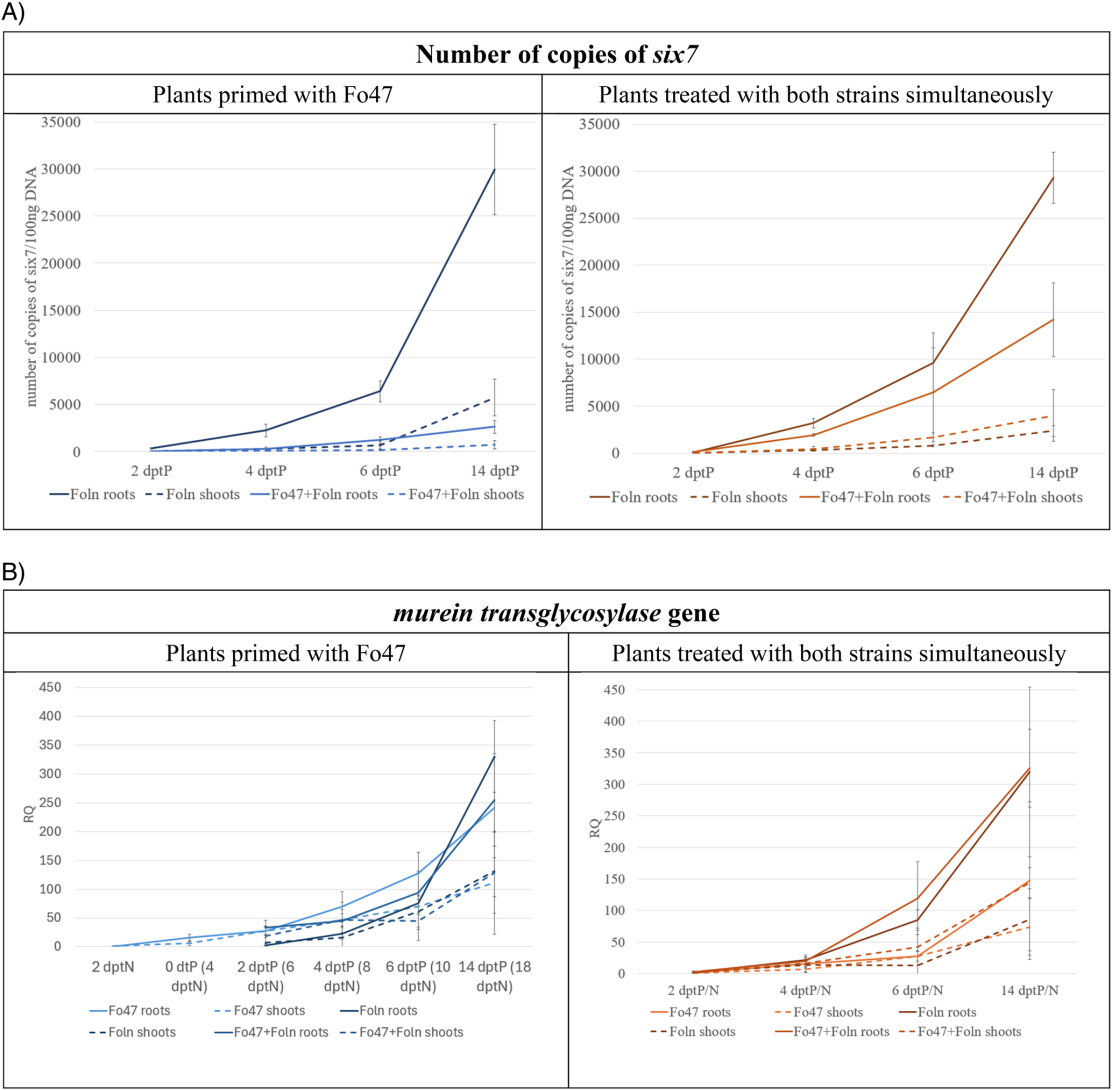
The progression of infection of plants primed with non‐pathogenic strain of *Fusarium oxysporum* and plants treated with both strains of *F. oxysporum* simultaneously. (A) Relative quantity of fungal *murein transglycosylase* gene in roots and shoots of plants primed with non‐pathogenic strain Fo47 and plants treated with both strains simultaneously. The data presented were obtained from real‐time PCR analysis. (B) Number of copies of the *six7* gene of pathogenic *Fusarium oxysporum* strain in the root and shoot of flax plants primed with the non‐pathogenic strain Fo47 and plants treated with both strains of the fungus simultaneously after 2, 4, 6 and 14 days of Foln treatment.

Amplification of the gene encoding murein transglycosylase (present in both *F. oxysporum* strains) was also performed using qPCR to more precisely determine the relative amount of the non‐pathogenic Fo47 strain in the plants (Figures 3A and S1B). The RQ values on the graph are presented in relation to the values obtained for samples treated with Foln on day 2. Over time, an increase in the content of fungal DNA in flax roots and shoots is visible, which confirms the multiplication of both strains in plant tissues. In the roots of plants treated with Fo47 and Foln and in primed plants, the amount of *murein transglycosylase* gene increases over time and on day 14 for both groups is over 500 times higher compared to the amount of fungal DNA in the roots treated with Foln at 2 dptP. It is worth noting that at 4 and 6 dptP in the roots of primed plants, the amount of *murein transglycosylase* gene was higher than in the roots treated with Foln, but lower than in the plants treated with Fo47, which results from the presence of both fungi in the plant. In the roots of the second research group, an increase in the content of fungal DNA was also observed over time. The fastest rate of increase in fungal DNA content was recorded in the roots of plants simultaneously treated with Foln and Fo47. In the shoots of plants from both treatments, we notice an increase in the content of fungal DNA over time for all types of plants tested. In the primed plants, at 2 and 4 dptP, a similar content of fungus is observed in plants treated with Fo47 and primed plants, and significantly less fungus in plants treated with Foln. In the second group of plants, at 4 dptP the content of fungus is similar in plants treated with Foln and plants treated with both strains. However, in the following days, a greater amount of *murein transglycosylase* gene is noticeable in plants treated with both strains than in those treated only with Foln.

In order to determine the amount of the pathogenic *F. oxysporum* strain in the treated plants, the number of copies of the fungal *six7* (*secreted in xylem 7*) gene was determined (Figures 3B and S1C). The roots of primed plants showed a lower number of *six*7 gene copies compared to plants treated only with the pathogenic strain (by 93% at 2 dptP, 87% at 4 dptP, 81% at 6 dptP and 91% at 14 dptP). However, the roots of plants treated simultaneously with the pathogenic and non‐pathogenic strain showed a lower (by 39% at 4 dptP and 51% at 14 dptP) and a similar (at 2 and 6 dptP) number of *six7* copies than the plants treated with the pathogenic strain. The copy number of the *six7* gene is much lower in shoots than in roots. In the shoots of primed plants, similarly to the roots, a decrease in the amount of the *six7* gene was observed (by 56% at 4 dptP, 74% at 6 dptP and 88% at 14 dptP). The situation is different in the shoots of plants treated simultaneously with a pathogenic and non‐pathogenic strain, where at 4 and 14 dptP there were no differences in the number of copies of the *six7* gene, while at 2 dptP they contained a 2‐fold lower number of copies of the *six7* gene compared to plants treated with Foln, and at 6 dptP, they contained 2‐fold the number of *six7* copies.

### 3.4 Transcript Levels of *PR Genes* and Genes Encoding Antioxidant Enzymes in Flax Treatment With a Various Strains of *Fusarium oxysporum*

**FIGURE 4:**
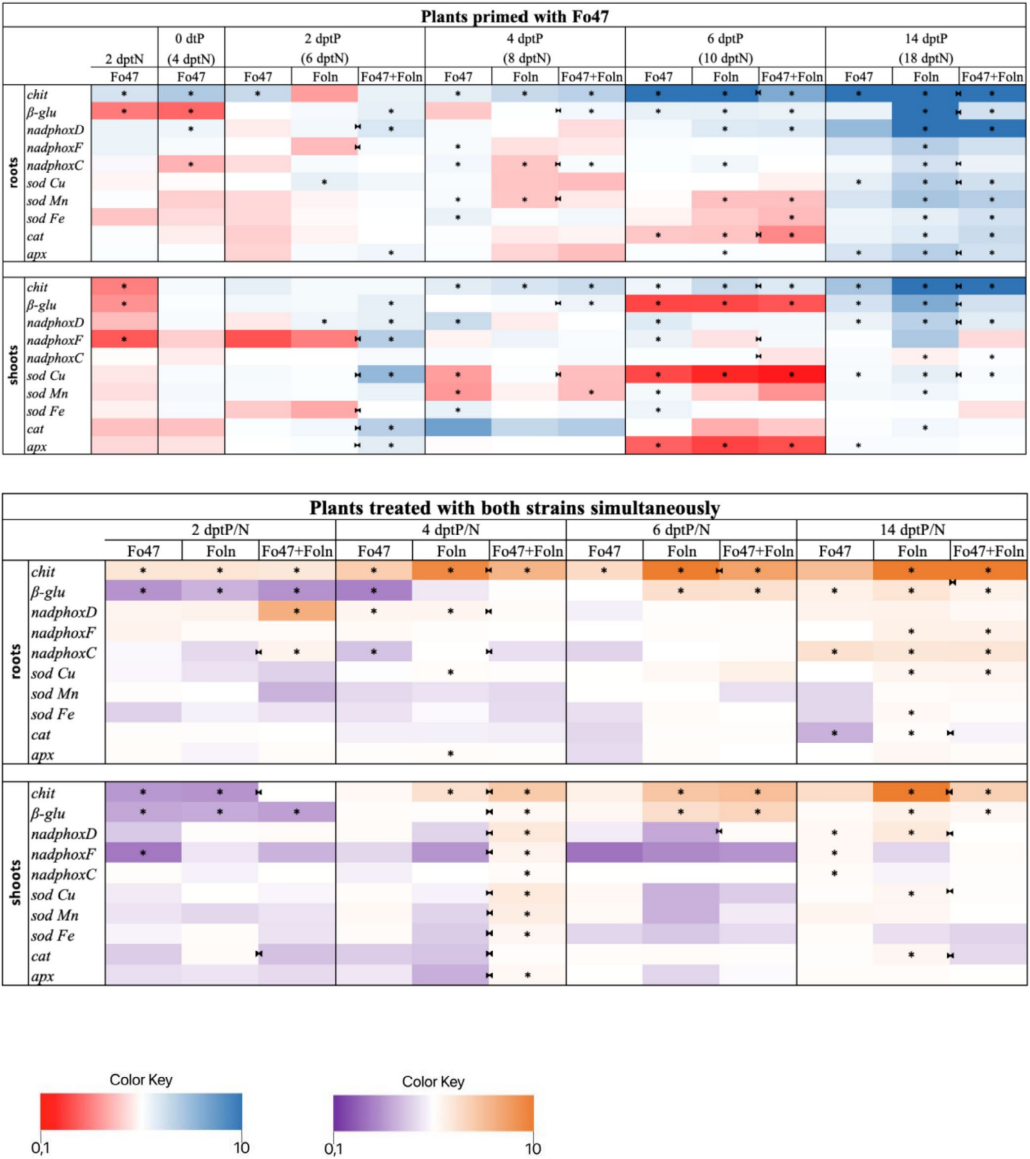
Transcript levels of *PR genes* and genes involved in ROS metabolism in roots and shoots of plants primed with non‐pathogenic strain of *Fusarium oxysporum* and plants treated with both strains of *F. oxysporum* simultaneously. Changes in transcript levels of *PR genes* (*chitinase* and *β‐glucanase*) and ROS metabolism genes (*NADPH oxidase D*, *NADPH oxidase F*, *and NADPH oxidase C*, three isoforms of *superoxide dismutase* (*sod*): *sod Cu/Zn, sod Mn, and sod Fe*, *catalase* and *ascorbate peroxidase*) are shown as relative quantity (RQ) to the reference gene (*actin*) for control. Results were obtained by real‐time PCR on a cDNA template. The significance of differences between groups was determined using a one‐way ANOVA, followed by Fisher's post hoc test. Differences were considered statistically significant when *p* < 0.05 (* for comparison to control, non‐treated plants from the same time point as the sample; ►◄ for comparison of plants primed with non‐pathogenic strain Fo47 or plants treated with both strains simultaneously with Foln treated plants from the same time point as sample).

In order to confirm the response of plants to treatment with fungal strains, the transcript levels of selected *PR genes*: *chitinase* (GenBank: AY847514.1) and *β‐1,3‐glucanase* (GenBank: JQ670874.1) were examined. Then, the mRNA levels of genes involved in ROS metabolism were checked. Analyzes were carried out at subsequent time points: 2, 4, 6, 14 dptP, as well as at points 2 and 4 dptN (0 dtP), before its addition. The results are presented as heatmaps in Figure 4 and in graphs with marked statistically significant changes in Figure S2.

In the roots of primed plants, the mRNA level of the gene encoding chitinase increased 4‐fold at 4 dptP, 6.6‐fold at 6 dptP, and 22.4‐fold at 14 dptP compared to untreated plants. However, it decreased by 60.9% and 64.5% at 6 and 14 dptP, respectively, compared to plants treated with Foln. In the roots of plants treated with Fo47, the *chitinase* transcript level significantly increased by over 15.6‐fold at 14 dptP, 11.2‐fold at 6 dptP, 2.13‐fold at 4 dptP, and 3.5‐fold at 2 dptP. Moreover, at the initial time point, the *chitinase* transcript level in flax roots was elevated by 4.4‐fold. In plants treated with Foln, transcript levels rose at 4, 6, and 14 dptP by 3.4‐fold, 16.8‐fold, and 63‐fold, respectively. In the shoots of plants treated with Fo47, an increase in mRNA levels of the gene encoding chitinase was observed at 4, 6, and 14 dptP by 2.3‐fold, 1.7‐fold, and 4.9‐fold, respectively. Similarly, in the shoots of plants treated with Foln, the *chitinase* transcript level increased (2.8‐fold at 2 dptP, 3.4‐fold at 4 dptP, and 35‐fold at 14 dptP). The shoots of primed plants exhibited an increase in mRNA levels of the *chitinase* gene at 4, 6, and 14 dptP by 3.3‐fold, 2.5‐fold, and 10.13‐fold, respectively, compared to untreated plants. However, a decrease of 25.5% and 71.1% in *chitinase* mRNA levels was noted at 6 and 14 dptP, respectively, compared to plants treated with Foln. In the experiment involving simultaneous treatment with both *F. oxysporum* strains, plants treated with the pathogenic strain showed a significant increase in *chitinase* mRNA levels in the roots at 2, 4, 6, and 14 dptP by 2.9‐fold, 9.2‐fold, 11.3‐fold, and 19‐fold, respectively. Flax treated with Fo47 exhibited an increase in *chitinase* transcript levels by 2.8‐fold at 2 dptP, 4.5‐fold at 4 dptP, and 3.5‐fold at 6 dptP compared to untreated flax. In plants treated simultaneously with both *F. oxysporum* strains, an increase in *chitinase* transcript levels was observed across all time points (2.7‐fold at 2 dptP, 6.3‐fold at 4 dptP, 7.5‐fold at 6 dptP, and 22‐fold at 14 dptP). Comparing plants treated with Foln and those treated with both Fo47 and Foln, a decrease in *chitinase* mRNA levels was noted at 4 and 6 dptP by 32% and 34%, respectively. In the shoots of plants treated with Fo47, a twofold decrease in *chitinase* mRNA levels was evident at 2 dptP. A similar decrease was observed in plants treated with Foln. At 4, 6, and 14 dptP, an increase in *chitinase* mRNA levels was noted in the shoots of plants treated with Foln by 3.1‐fold, 5‐fold, and 16.7‐fold, respectively, compared to untreated plants. On these days, an increase in the transcript levels of the gene encoding chitinase was also observed in flax treated simultaneously with both Fo47 and Foln (4.7‐fold at 4 dptP, 5.4‐fold at 6 dptP, and 4.3‐fold at 14 dptP). Comparing plants treated with Foln and those treated with both strains simultaneously, an increase in *chitinase* transcript levels was noted, being twofold at 2 dptP and 50% at 4 dptP, whereas a 74% decrease in *chitinase* mRNA levels was observed at 14 dptP.

The roots of plants treated with Fo47 exhibited a 54% decrease followed by a twofold increase in the mRNA level of the *β‐glucanase* gene at 0 and 6 dptP, respectively. The roots of plants treated with Foln displayed higher levels of *β‐glucanase* mRNA, with a twofold increase at 6 dptP and an 11‐fold increase at 14 dptP. Primed plants showed an increase in the mRNA levels of the *β‐glucanase* gene in the roots: twofold at 2 and 6 dptP, 1.6‐fold at 4 dptP, and threefold at 14 dptP. Comparing primed plants with those treated with Foln, the roots of primed plants showed a 3.7‐fold decrease in *β‐glucanase* mRNA levels at 14 dptP but a 46% increase at 4 dptP. Similarly, in the shoots of primed plants, a 53% decrease in *β‐glucanase* mRNA levels was observed at 14 dptP, while a 38% increase occurred at 4 dptP compared to plants treated with Foln. Plants treated solely with the pathogenic strain exhibited a 6.5‐fold increase in the mRNA levels of the *β‐glucanase* gene in shoots compared to the control, but only at 14 dptP. In the shoots of plants treated with Fo47, no significant changes in *β‐glucanase* transcript levels were observed. In the second group of plants, treated with both Fo47 and Foln simultaneously, a 3.1‐fold increase in *β‐glucanase* mRNA levels was observed in the roots at 6 dptP and a twofold increase at 14 dptP, while a 47% decrease was noted at 2 dptP. In the shoots of plants treated with Fo47, *β‐glucanase* transcript levels were approximately 50% lower at 2 and 4 dptP but twofold higher at 14 dptP. In contrast, in the shoots of plants treated with Foln, a 33% decrease in *β‐glucanase* transcript levels was observed at 2 dptP, followed by a 3.2‐fold and 2.9‐fold increase at 6 and 14 dptP, respectively. Comparing plants treated with both strains of *F. oxysporum* to those treated with Foln, a 34% decrease in *β‐glucanase* mRNA levels at 14 dptP was observed in the roots of the former group. In the shoots of plants treated with Foln, a 3.3‐fold and twofold increase in *β‐glucanase* mRNA levels was observed at 6 and 14 dptP, respectively, along with a 37% decrease at 2 dptP. A similar decrease was observed in plants treated with Fo47. In the shoots of plants treated with both Fo47 and Foln simultaneously, approximately a 1.5‐fold increase in *β‐glucanase* transcript levels was observed at 4 and 14 dptP, a fourfold increase at 6 dptP, and a 1.7‐fold decrease at 2 dptP. Furthermore, in the shoots of these plants, a 33% increase in *β‐glucanase* transcript levels was observed at 4 dptP compared to plants treated with Foln.

The analysis focused on the mRNA levels of selected *NADPH oxidase* genes: *NADPH oxidase D, NADPH oxidase F, and NADPH oxidase C*. In the roots of plants treated with Fo47, only a 70% increase in the transcript level of the gene encoding NADPH oxidase D was observed at time 0 dptP (dptN). In contrast, roots of plants treated with Foln exhibited a 2.4‐fold and 12.5‐fold increase at 6 and 14 dptP, respectively. Similarly, in the roots of primed plants, the mRNA level of *NADPH oxidase D* increased 2.7‐fold at 2 dptP, 2.6‐fold at 6 dptP, and 11.5‐fold at 14 dptP compared to control plants, with a 1.7‐fold increase at 2 dptP compared to plants treated with Foln. In the shoots of primed plants, a 2.1‐fold and 2.3‐fold increase in *NADPH oxidase D* mRNA level was observed at 2 and 14 dptP, respectively, compared to control plants, while a 1.7‐fold decrease was noted at 14 dptP compared to Foln‐treated plants. Shoots of Foln‐treated plants showed a fourfold increase in *NADPH oxidase D* mRNA levels at 14 dptP and a 1.8‐fold increase at 2 dptP. In Fo47‐treated plants, the shoots exhibited a 1.9‐fold increase in transcript levels at 14 dptP, 2.3‐fold at 6 dptP, and 3.3‐fold at 4 dptP. In roots of plants treated with both Fo47 and Foln, a 6.5‐fold increase in *NADPH oxidase D* mRNA levels was observed at 2 dptP compared to untreated plants, while a 25% decrease was noted at 4 dptP compared to Foln‐treated plants. In the shoots of these plants, a 2.6‐fold increase in mRNA levels was recorded at 4 dptP compared to untreated plants, and a 3.2‐fold and twofold increase in mRNA levels was observed at 4 and 6 dptP, respectively, with a 2.3‐fold decrease at 14 dptP compared to Foln‐treated plants. Furthermore, at 4 dptP, a 70% increase in *NADPH oxidase D* mRNA levels was recorded in the roots of plants treated with both Fo47 and Foln, while in the shoots, a 2.6‐fold and 1.5‐fold increase was observed at 14 dptP in plants treated with the non‐pathogenic and pathogenic strains, respectively.

No differences in *NADPH oxidase F* mRNA levels were observed in the roots of Fo47‐primed plants compared to untreated plants, except for a 79.5% increase at 4 dptP relative to Foln‐treated plants. In the shoots of primed plants, a 4.2‐fold increase in *NADPH oxidase F* mRNA levels was observed at 2 dptP compared to untreated plants, with a 7.6‐fold and 1.7‐fold increase at 2 and 6 dptP, respectively, compared to Foln‐treated plants. In roots, a 1.4‐fold and 4.2‐fold increase in mRNA levels was observed in plants treated with Fo47 at 4 dptP and Foln at 14 dptP, respectively. In the shoots of Fo47‐treated plants, only a 1.9‐fold increase in *NADPH oxidase F* mRNA levels was noted at 6 dptP compared to control plants. In roots of plants treated with both Fo47 and Foln, approximately a twofold increase in *NADPH oxidase F* mRNA levels was observed at 14 dptP compared to untreated plants, with a similar increase in plants treated with the pathogenic strain. In the shoots of plants treated with both strains, only a 1.8‐fold increase in *NADPH oxidase F* mRNA levels was noted at 4 dptP compared to untreated plants and a 3.4‐fold increase compared to Foln‐treated plants. In shoots of plants treated with the non‐pathogenic strain, a 1.5‐fold increase in *NADPH oxidase F* mRNA levels was observed at 14 dptP, while a 2.4‐fold decrease was recorded at 2 dptP.

The analysis of *NADPH oxidase C* mRNA levels revealed a 27% increase in the roots of primed plants at 4 dptP compared to untreated plants, with a 1.6‐fold increase on the same day and a 38% decrease at 14 dptP compared to Foln‐treated plants. In the shoots of these plants, only a 26% decrease in *NADPH oxidase C* transcript levels was observed at 6 dptP compared to Foln‐treated plants, along with a slight increase at 14 dptP compared to untreated plants. Roots of Foln‐treated plants exhibited a 62% and threefold increase in transcript levels at 6 and 14 dptP, respectively, with a 20% decrease at 4 dptP. Roots of Fo47‐treated plants showed a 59% increase in *NADPH oxidase C* mRNA levels at 4 dptP. In roots of plants treated with both strains, mRNA levels of *NADPH oxidase C* were elevated 1.86‐fold at 2 dptP and 3.08‐fold at 14 dptP compared to untreated plants, with a 2.2‐fold increase at 2 dptP and a 19% decrease at 4 dptP compared to Foln‐treated plants. In the shoots of these plants, a 30% increase was observed only at 4 dptP compared to untreated plants. A 3.1‐fold and 2.8‐fold increase in transcript levels was observed at 14 dptP in the roots of plants treated with Fo47 and Foln, respectively. Additionally, the shoots of Fo47‐treated plants contained 34% more mRNA of the gene at 14 dptP.

The mRNA levels of the gene encoding three isoforms of superoxide dismutase (SOD): SOD Cu/Zn, SOD Mn, and SOD Fe were analysed. In the roots of primed plants at 14 dptP, a threefold increase in *sodCu* mRNA was observed compared to untreated plants, along with a 27.2% decrease compared to plants treated with Foln. Additionally, a 1.26‐fold and 2.1‐fold increase in *sodCu* transcript levels was observed at 4 and 14 dptP in the roots of plants treated with Fo47, and a 2.1‐fold and 4.2‐fold increase at 2 and 14 dptP in plants treated with Foln. In the shoots of primed plants, a 5.7‐fold and 1.35‐fold increase in *sodCu* mRNA levels was observed at 2 and 14 dptP, respectively, and an 81% decrease at 6 dptP compared to untreated plants. Furthermore, a 4.8‐fold increase and approximately 37% decrease in *sodCu* transcript levels were noted at 4 and 14 dptP in comparison to plants treated with Foln. Shoots of Fo47‐treated plants exhibited a 34% and 64% decrease and a 1.4‐fold increase in *sod Cu* transcripts at 4, 6, and 14 dptP, respectively. Foln treatment resulted in a 76% decrease and a 2.2‐fold increase in *sodCu* transcript levels at 6 and 14 dptP, respectively. In roots treated with both Fo47 and Foln simultaneously, a 1.8‐fold increase in *sodCu* mRNA was observed at 14 dptP compared to untreated plants. Foln treatment caused the same transcript changes at 14 dptP and an additional 1.27‐fold increase at 4 dptP. Shoots treated with both *F. oxysporum* strains showed a 2.5‐fold increase in *sodCu* mRNA at 4 dptP compared to untreated controls and a 2.6‐fold increase compared to Foln‐treated shoots. At 14 dptP, shoots treated with both Fo47 and Foln exhibited a 31.7% lower *sodCu* mRNA level than shoots treated with Foln alone, which in turn showed a 67% increase compared to untreated plants.

In the roots of primed plants, *sodMn* mRNA levels decreased by 22% at 6 dptP and increased 4.2‐fold at 14 dptP compared to untreated plants, with a slight increase at 4 dptP compared to Foln‐treated plants. *sodMn* transcript levels decreased significantly in the roots of Foln‐treated plants at 4 and 6 dptP by 20% and 21.8%, respectively, but increased nearly fivefold at 14 dptP compared to controls. Roots treated with Fo47 displayed only a 40% increase in *sodMn* mRNA at 4 dptP. In the shoots of primed plants, a 24% decrease in *sodMn* mRNA level was recorded at 4 dptP compared to untreated plants. Shoots treated with Foln had 1.7 times higher *sodMn* transcript level at 14 dptP, while those treated with Fo47 showed a 36% decrease and a 1.5‐fold increase at 4 and 6 dptP, respectively. In plants treated with both strains, only a 2.2‐fold increase in *sodMn* transcripts was observed in shoots at 4 dptP compared to untreated plants and a 2.8‐fold increase compared to Foln‐treated plants.

In primed plant roots, *sodFe* transcript levels showed a 24.4% decrease at 6 dptP and a nearly threefold increase at 14 dptP compared to untreated plants. A twofold increase in *sodFe* mRNA was also observed at 14 dptP in roots treated with the pathogenic strain, along with a 1.87‐fold increase at 4 dptP in roots treated with Fo47. Analysis of shoots revealed a 1.5‐fold increase in *sodFe* mRNA level at 2 dptP compared to Foln‐treated plants and a twofold and 1.75‐fold increase in transcripts at 4 and 6 dptP in shoots treated with Fo47. In the second group of plants (treated with both Fo47 and Foln), significant changes in *sodFe* mRNA levels were noted in roots of Foln‐treated plants (a 1.46‐fold increase at 14 dptP) and in the shoots of primed plants (a 1.6‐fold increase at 4 dptP compared to untreated plants and a twofold increase compared to Foln‐treated plants).

In primed plant roots, *catalase* transcript levels decreased by 42% at 6 dptP and increased 3.4‐fold at 14 dptP compared to untreated plants, with a 27% decrease at 6 dptP compared to Foln‐treated plants. Additionally, a 20% decrease in *catalase* mRNA levels was observed in roots treated with Fo47 or Foln at 6 dptP, and a 2.4‐fold increase at 14 dptP in Foln‐treated roots. In shoots at 2 dptP, a 4.1‐fold increase in *catalase* transcript levels was observed in primed plants compared to controls, and a 2.3‐fold increase compared to Foln‐treated shoots, with a 1.6‐fold increase in transcripts at 14 dptP in Foln‐treated shoots. Roots treated with both strains showed a 25% decrease in *catalase* mRNA level at 14 dptP compared to Foln‐treated roots. Additionally, a 1.26‐fold increase and a 34% decrease in *catalase* transcript levels were observed at 14 dptP in roots treated with Foln and Fo47, respectively. Shoots treated with both *F. oxysporum* strains exhibited a 1.6‐fold increase in *catalase* mRNA at 4 dptP compared to those treated with the pathogenic strain alone, while a 1.8‐fold and twofold decrease was observed at 2 and 14 dptP, respectively. At 14 dptP, a 1.65‐fold increase in *catalase* transcripts was observed in Foln‐treated shoots.

The transcript levels of *ascorbate peroxidase* (*apx*) were also analysed. In the roots of primed plants, a 1.7‐fold and threefold increase in *apx* mRNA levels was observed at 2 and 14 dptP compared to untreated plants, with a 26.7% decrease at 14 dptP compared to Foln‐treated plants. Moreover, in Foln‐treated roots, a 1.2‐fold and 4.1‐fold increase in *apx* transcripts was observed at 6 and 14 dptP, respectively, while a 2.8‐fold increase was noted at 14 dptP in Fo47‐treated roots. In the shoots of primed plants, a 2.2‐fold increase in *apx* mRNA levels was recorded at 2 dptP compared to untreated plants, and a 1.4‐fold increase compared to Foln‐treated plants. Additionally, at 6 dptP, a 63%, 55%, and 66% decrease in *apx* mRNA levels was observed in shoots of primed, Fo47‐treated, and Foln‐treated plants, respectively. At 14 dptP, a 1.3‐fold increase in *apx* transcripts was noted in the shoots of Fo47‐treated plants. In shoots treated with both strains, a 1.55‐fold increase in *apx* mRNA levels was observed at 4 dptP compared to untreated plants, and a 2.38‐fold increase compared to Foln‐treated plants.

### 3.5 Superoxide Dismutase and Catalase Activity in Flax Treatment With a Various Strains of *Fusarium oxysporum*

**FIGURE 5:**
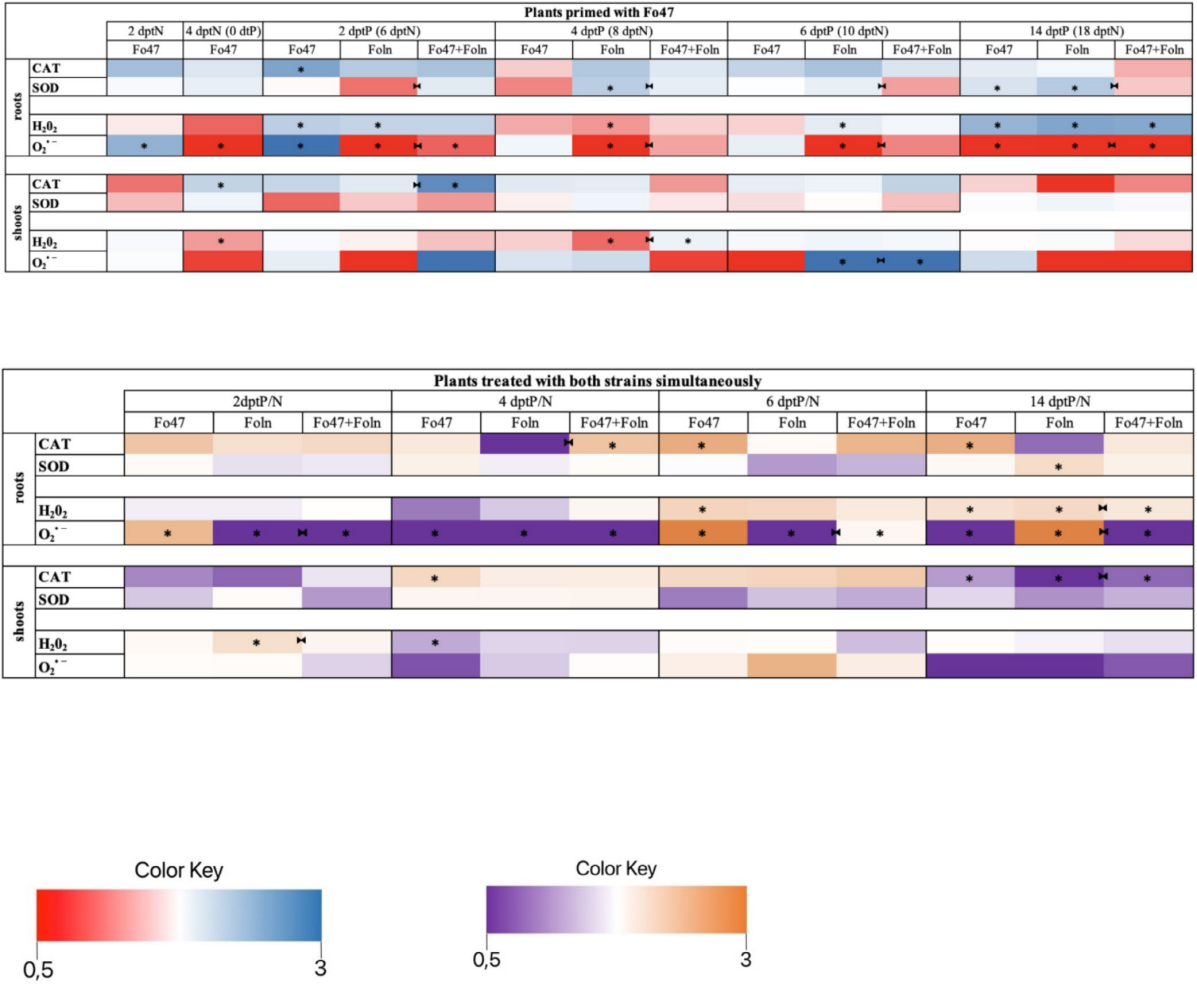
Catalase and superoxide dismutase activity and H_2_O_2_ and O_2_^•–^ content in roots and shoots of plants primed with non‐pathogenic strain of *Fusarium oxysporum* and plants treated with both strains of *F. oxysporum* simultaneously. The significance of differences between groups was determined using a one‐way ANOVA, followed by Fisher's post hoc test. Differences were considered statistically significant when *p* < 0.05 (* for comparison to control, non‐treated plants from the same time point as the sample; ►◄ for comparison of plants primed with non‐pathogenic strain Fo47 or plants treated with both strains simultaneously with Foln treated plants from the same time point as sample).

The activity of superoxide dismutase (SOD) and catalase (CAT) in flax plants primed with the non‐pathogenic strain of *F. oxysporum* and in flax plants treated simultaneously with both non‐pathogenic and pathogenic strains of *F. oxysporum* is presented in Figures 5 and S3. The most significant changes in SOD activity were observed in the roots of primed plants, whereas no changes were detected in the shoots of either sensitised plants or plants treated with both strains simultaneously.

In the roots of primed plants, SOD activity did not differ statistically significantly compared to untreated plants. However, compared to plants treated with Foln, root SOD activity increased 1.8‐fold at 2 dptP and decreased by 27%, 34%, and 49% at 4, 6, and 14 dptP, respectively. Additionally, a 1.4‐fold increase in SOD activity was observed in the roots of plants treated with Fo47 at 14 dptP, as well as a 1.7‐fold increase in the roots of plants treated with Foln at 4 and 14 dptP. In the group of plants treated with both strains simultaneously, only in the roots of plants treated with Foln was a 1.6‐fold increase in SOD activity observed at 14 dptP.

In the group of primed plants, catalase activity in the roots increased 2.3‐fold only at 2 dptP in plants treated with Fo47. In the shoots, at 2 dptP, CAT activity increased 2.65‐fold and 2‐fold in primed plants compared to untreated plants and plants treated with Foln, respectively. In the group of plants treated with both strains simultaneously, a 2.3‐fold increase in CAT activity was observed in the roots of plants treated with Fo47 at 6 and 14 dptP. Additionally, at 4 dptP, CAT activity in the roots of plants treated with both strains simultaneously increased 1.95‐fold and 4.6‐fold compared to untreated plants and plants treated with Foln, respectively. At 14 dptP, CAT activity in the shoots decreased by 24%, 50%, and 36% in plants treated with Fo47, plants treated with Foln, and plants treated with both strains simultaneously, respectively, compared to untreated plants. However, at 14 dptP, a 1.3‐fold increase in CAT activity was observed in plants treated with both strains simultaneously compared to plants treated with Foln. Furthermore, at 4 dptP, CAT activity in the shoots of plants treated with Fo47 increased 1.6‐fold.

### 3.6 Hydrogen Peroxide and Superoxide Anion Content in Flax Treatment With a Various Strains of *Fusarium oxysporum*

The changes in H_2_O_2_ and O_2_^• –^ content in the studied plants are presented in Figure 5 and Figure S3C. The H_2_O_2_ content was 2.3 times higher at 14 dptP in the roots of primed plants and those treated with Foln, and 2.1 times higher in plants treated with Fo47. Additionally, the roots of plants treated with Fo47 exhibited a 1.7‐fold increase in H_2_O_2_ content at 2 dptP, while the roots of plants treated with Foln showed 1.6‐fold and 1.3‐fold increases at 2 and 6 dptP, respectively, along with a 21% decrease at 4 dptP. In the shoots of primed plants at 4 dptP, a 1.7‐fold increase in H_2_O_2_ content was observed compared to plants treated with Foln and a 1.2‐fold increase compared to untreated plants. Moreover, in the shoots of plants treated with Foln at 4 dptP, a 30% decrease in H_2_O_2_ content was observed. Additionally, a 21% decrease in H_2_O_2_ content was recorded in the shoots of plants treated with Fo47 at 0 dptP.

In the roots of primed and Foln‐treated plants, compared to the roots of untreated plants, the O_2_^•–^ content was 33% and 52% lower at 2 and 14 dptP, respectively, whereas, compared to the roots of Foln‐treated plants, the O_2_^•–^ content was 1.5, 1.9, 2.4, and 3.6 times higher at 2, 4, 6, and 14 dptP, respectively. The roots of Fo47‐treated plants showed a 2.1 and 11.4‐fold increase in O_2_^•–^ content at 2 and 6 dptN and a 64% and 49% decrease at 4 and 18 dptN, and the roots of Foln‐treated plants showed a 55%, 58%, 69%, and 86% decrease at 2, 4, 6, and 14 dptP, respectively. In shoots, only changes in O_2_^•–^ content were observed at 6 dptP: a 106‐fold increase in shoots of primed and Foln‐treated plants compared to untreated plants and a 2.9‐fold increase in shoots of primed and Foln‐treated plants compared to Foln‐treated plants and a 37‐fold increase in shoots of Foln‐treated plants.

The roots of plants treated simultaneously with both fungal strains contained 1.4 times more H_2_O_2_ at 14 dptP compared to untreated plants and 13% less H_2_O_2_ compared to plants treated with Foln. Treatment with Fo47 resulted in a 1.7‐fold and 1.5‐fold increase in H_2_O_2_ content in roots at 6 and 14 dptP, respectively, while treatment with Foln increased H_2_O_2_ content in roots 1.6 times at 14 dptP. In the shoots of plants treated simultaneously with both strains at 2 dptP, the H_2_O_2_ content remained unchanged compared to untreated plants but showed a 23% decrease compared to plants treated with Foln. At 4 dptP, the shoots of plants treated with Fo47 contained 21% less H_2_O_2_.

The roots of plants treated simultaneously with both fungal strains contained 76%, 84%, and 82% less O_2_^•–^ at 2, 4, and 14 dptP and 1.1‐fold more at 6 dptP compared to untreated plants and 52% and 94% less O_2_^•–^ at 2 and 14 dptP, respectively, and 14.6‐fold more at 6 dptP compared to plants treated with Foln. Fo47 treatment caused a 2.1‐fold and 11.4‐fold increase in O_2_^•–^ content at 2 and 6 dptP, respectively, and a 64% and 69% decrease at 4 and 14 dptP, respectively, while Foln treatment caused a 50%, 84%, and 92% decrease at 2, 4, and 6 dptP, respectively, and a 3‐fold increase in O_2_^•–^ content at 14 dptP. However, no changes were observed in the shoots.

### 3.7 Phenolic Compounds Content in Flax Treatment With a Various Strains of *Fusarium oxysporum*

**FIGURE 6:**
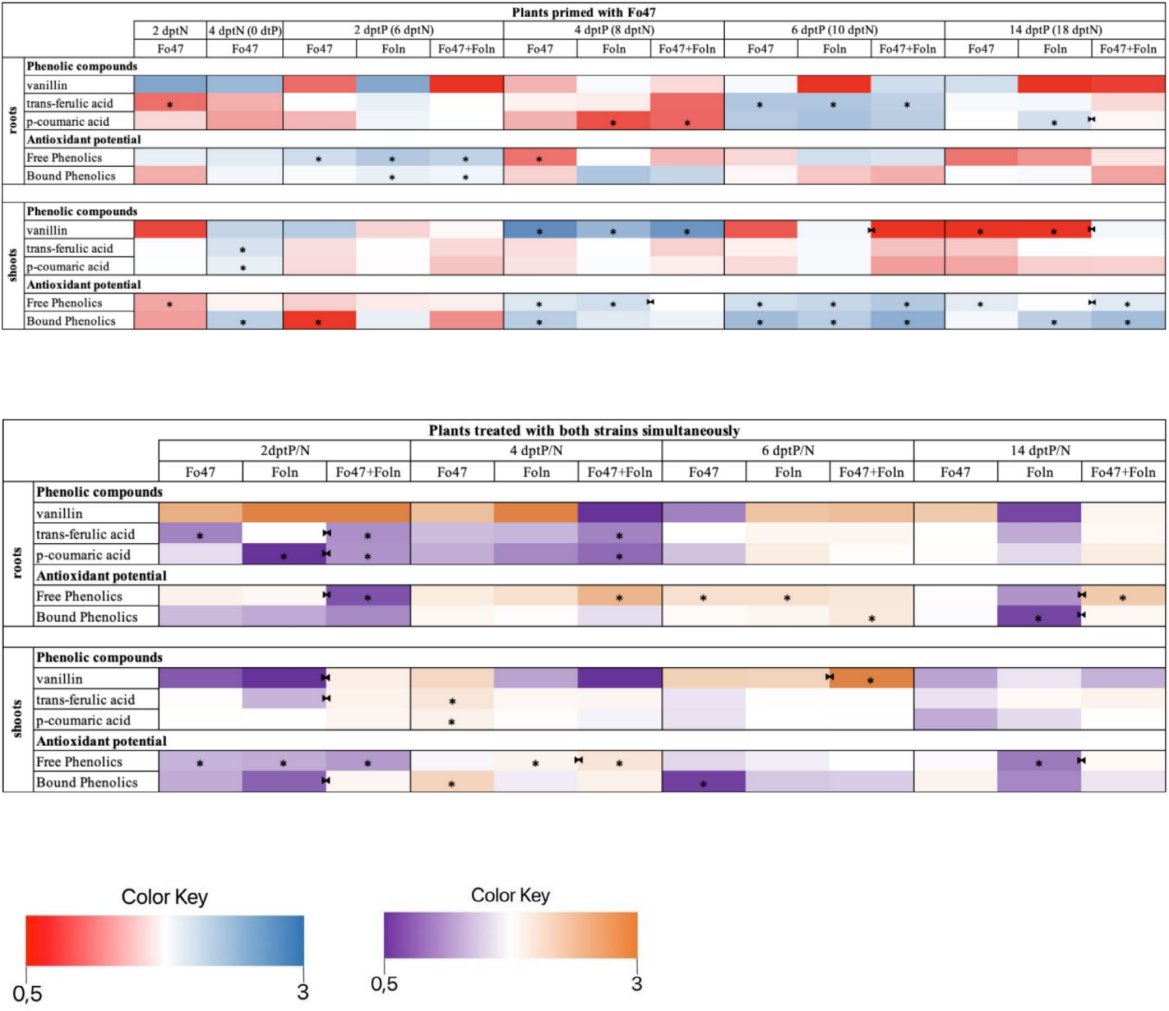
Phenolic compound content (vanillin, *trans*‐ferulic acid and *p*‐coumaric acid) and antioxidant potential (in extract including free phenolic compounds and in extract bound phenolic compounds) from roots and shoots of plants primed with non‐pathogenic strain of *Fusarium oxysporum* and plants treated with both strains of *Fusarium oxysporum* simultaneously. The significance of differences between groups was determined using a one‐way ANOVA, followed by Fisher's post hoc test. Differences were considered statistically significant when *p* < 0.05 (* for comparison to control, non‐treated plants from the same time point as the sample; ►◄ for comparison of plants primed with non‐pathogenic strain Fo47 or plants treated with both strains simultaneously with Foln treated plants from the same time point as sample).

The contents of phenolic compounds (vanillin, trans‐ferulic acid, and *p*‐coumaric acid) in the roots and shoots of the analysed plants are presented in Figure 6 and Table S2. In the roots of primed plants and treated with Foln, a 1.7‐fold increase in ferulic acid content was observed at 6 dptP and a 32% decrease in *p*‐coumaric acid content at 4 dptP compared to untreated plants, and a 33% decrease in *p*‐coumaric acid content at 14 dptP compared to Foln‐treated plants. Furthermore, in the roots of plants treated with Fo47, ferulic acid content was 30% lower at 2 dptN and 1.8‐fold higher at 6 dptP. In the roots of plants treated with Foln, ferulic acid content increased 18‐fold at 6 dptP, and *p*‐coumaric acid content decreased by 38% at 4 dptP and increased 1.4‐fold at 14 dptP compared to untreated plants. However, in the shoots of primed plants and treated with Foln, a 2.5‐fold increase in vanillin content was observed at 4 dptP compared to untreated plants, and a 64% decrease in vanillin content at 6 dptP, and a 2.2‐fold increase in vanillin content at 14 dptP compared to Foln‐treated plants. In the shoots of plants treated with Fo47, there was 1.4‐fold more ferulic acid and 1.2‐fold more *p*‐coumaric acid at 4 dptN, 2.7‐fold more vanillin at 4 dptP, and 58% less vanillin at 14 dptP, while in the shoots of plants treated with Foln, there was 2‐fold more vanillin at 4 dptP and 50% less vanillin at 14 dptP.

In the roots of plants treated simultaneously with both fungal strains, there was 37% less ferulic acid and 26% less *p*‐coumaric acid at 2 dptP and 30% less ferulic acid and 37% less *p*‐coumaric acid at 4 dptP compared to untreated plants, and 28% less ferulic acid and 10‐fold more *p*‐coumaric acid at 2 dptP compared to plants treated with Foln. Furthermore, in the roots of plants treated with Fo47, ferulic acid was 30% less at 2 dptN, and in the roots of plants treated with Foln, the content of *p*‐coumaric acid decreased by 99% at 4 dptP compared to untreated plants. However, in shoots of plants treated simultaneously with both fungal strains, a 3.4‐fold increase in vanillin content was observed at 6 dptP compared to untreated plants, a 3.2‐fold increase in vanillin content and a 1.5‐fold increase in ferulic acid content at 2 dptP, and a 2‐fold increase in vanillin at 6 dptP compared to plants treated with Foln. In shoots of plants treated with Fo47, there was 1.4‐fold more ferulic acid and 1.2‐fold more *p*‐coumaric acid at 4 dptN compared to untreated plants.

### 3.8 Antioxidant Potential in Flax Treatment With a Various Strains of *Fusarium oxysporum*

Antioxidant potential was determined for extracts containing free phenolic compounds and bound phenolic compounds. The results are presented in Figures 6 and S4. The antioxidant potential of extracts containing free phenolic compounds was 1.5‐, 1.8‐, and 1.6‐fold higher at 2 dptP from roots of Fo47‐treated, Foln‐treated, and primed and Foln‐treated plants, respectively, compared to untreated plants and 29% lower at 4 dptP from roots of Fo47‐treated plants. Similarly, the antioxidant potential of extracts containing bound phenolic compounds was 1.2‐fold higher at 2 dptP from roots of Foln‐treated and primed and Foln‐treated plants. The antioxidant potential of extracts containing free phenolic compounds from shoots of primed and Foln‐treated plants was 1.8 and 1.3‐fold higher at 6 and 14 dptP compared to untreated plants and 32% lower at 4 dptP and 1.3‐fold higher at 14 dptP compared to Foln‐treated plants; 18% lower at 2 dptN and 1.4, 1.5 and 1.3‐fold higher at 4, 6 and 14 dptP, respectively, from shoots of Fo47‐treated plants; 1.5 and 1.6‐fold higher at 4 and 6 dptP, respectively, from shoots of Foln‐treated plants. In the case of the antioxidant potential of shoot extracts containing bound phenolic compounds, it was 2.2 and 1.9‐fold higher at 6 and 14 dptP from primed and Foln‐treated plants, respectively, compared to untreated plants; 47% lower at 2 dptP and 1.7, 1.7 and 2‐fold higher at 0, 4 and 6 dptP from shoots of Fo47‐treated plants, respectively; 1.7‐fold higher at 6 and 14 dptP from shoots of Foln‐treated plants.

The antioxidant potential of extracts containing free phenolic compounds from roots of plants treated simultaneously with both fungal strains was 42% lower at 2 dptP and 2.2‐ and 1.8‐fold higher at 4 and 14 dptP, respectively, compared to untreated plants, and 48% lower at 2 dptP and 2.48‐fold higher at 14 dptP compared to plants treated with Foln. Additionally, a 1.5‐fold higher antioxidant potential was observed at 6 dptN from shoots of plants treated with both Fo47 and Foln. In contrast, the antioxidant potential of extracts containing bound phenolic compounds was 1.4‐fold higher at 6 dptP from roots of plants treated simultaneously with both fungal strains compared to untreated plants, twice as high at 14 dptP compared to plants treated with Foln, and 45% lower from roots of plants treated with Foln. The antioxidant potential of extracts containing free phenolic compounds from shoots of plants treated simultaneously with both fungal strains was 25% lower at 2 dptP and 1.4‐fold higher at 4 dptP compared to untreated plants, and 1.2‐ and 1.6‐fold higher at 4 and 14 dptP, respectively, compared to plants treated with Foln. Additionally, the antioxidant potential was 18% lower at 2 dptN from shoots of plants treated with Fo47, and 20% and 32% lower at 2 and 14 dptP, respectively, and 1.2‐fold higher at 4 dptP from shoots of plants treated with Foln. In the case of shoot extracts containing bound phenolic compounds, the antioxidant potential was: 1.9‐fold higher at 2 dptP shoots of plants treated simultaneously with both fungal strains and 1.7‐fold higher at 4 dptP and 47% lower at 6 dptP shoots of plants treated with Fo47.

## 4 Discussion

Plants in their natural environment must activate specialised adaptive mechanisms to enhance resistance against stress factors. One such mechanism is priming, a process in which short‐term exposure to mild stress triggers the activation of defence mechanisms, enabling a faster and stronger response to subsequent stress (Lämke and Bäurle 2017). Among various factors that prepare plants for defence, non‐pathogenic microbial strains can enhance plant immunity by acting on seeds or plants.

The objective of this study was to confirm that the non‐pathogenic strain *F. oxysporum* Fo47, functioning as a priming agent through root colonisation, significantly restricts the development of flax disease caused by the pathogenic strain *F. oxysporum* Foln. Furthermore, we aimed to determine whether Fo47 induces resistance through the activation of the antioxidant system.

Two independent plant treatments were conducted. In the first treatment, to assess whether *F. oxysporum* Fo47 acts as a priming agent, flax plants were treated with Fo47, followed by the addition of the pathogenic strain Foln after 4 days. In the second treatment, to confirm this effect, flax plants were simultaneously inoculated with both strains (non‐primed plants). Phenotypic analysis of infected plants, as well as calculated disease index, at 28 and 42 dptP revealed that the non‐pathogenic *F. oxysporum* significantly suppressed the progression of flax fusariosis. Notably, in primed plants, disease symptom suppression was considerably greater than in plants where Fo47 was introduced into the substrate simultaneously with Foln. These findings suggest that Fo47 functions as a priming agent, substantially enhancing flax resistance to Fusarium wilt, while also exerting a moderate suppressive effect when co‐applied with Foln. The disease‐suppressing effects of Fo47, confirmed phenotypically, have also been demonstrated in *Capsicum annuum* (pepper) infected with *Verticillium dahliae* (Veloso and Díaz 2021), sugar beet (*Beta vulgaris L*.) infected with *Beet Necrotic Yellow Vein Virus* (Nouayti et al. 2018), and tomato infected with *F. oxysporum* f. sp. *lycopersici*. Additionally, similar effects of non‐pathogenic *F. oxysporum* strains have been observed in cotton and olive ( *Olea europaea* subsp. *europaea*) infected with *Verticillium dahliae* (Zhang et al. 2015; Mulero‐Aparicio et al. 2019) as well as in strawberry roots infected with *Gnomonia fragariae* (Moročko‐Bičevska et al. 2014).

To elucidate the priming mechanism of the non‐pathogenic Fo47, we quantified the levels of both pathogenic and non‐pathogenic *F. oxysporum* strains, analysed transcript levels of *PR genes* and antioxidant system‐related genes, measured catalase and superoxide dismutase activity, and assessed hydrogen peroxide and superoxide anion levels and phenolic compound content and antioxidant potential in primed plants and those simultaneously treated with both strains.

Microscopic analysis revealed that individual Foln hyphae appeared in the roots as early as 4 dptP, whereas Fo47 hyphae were detectable only at 8 dptN. Moreover, at 14 dptP, a significantly higher presence of the pathogenic *F. oxysporum* was observed compared to the non‐pathogenic Fo47. This suggests a faster penetration rate and more extensive root colonisation by Foln. Consistent results were obtained by Bolwerk et al., demonstrating faster and more intense colonisation of tomato root cells by *F. oxysporum* f. sp. *radicis‐lycopersici* compared to non‐pathogenic *F. oxysporum* Fo47 (Bolwerk et al. 2005). However, a different pattern was observed in *Arabidopsis thaliana* and *Solanum lycopersicum* infected with pathogenic and non‐pathogenic *F. oxysporum*, where the rates of colonisation and penetration of root cells were similar for both strains (Nahalkova et al. 2008; Martínez‐Soto et al. 2023). However, PCR analysis targeting Fo47‐ and Foln‐specific DNA fragments and the relative expression of *mureine transglycosylase* indicated the presence of both strains in flax roots as early as 4 dptP/N. Despite this, the abundance of Fo47 in the roots was lower than that of Foln, further supporting the notion that Foln infiltrates plant tissues more rapidly. Additionally, Foln exhibited a faster colonisation rate, as evidenced by higher levels of *mureine transglycosylase* in both roots and shoots at 6 and 14 dptP in Foln‐treated plants compared to those treated with Fo47. A similar trend was observed in tomato plants, where tissue colonisation by the pathogenic *F. oxysporum* (*Fox* f. sp. *radicis‐lycopersici* ZUM2407) was tenfold higher than that of the non‐pathogenic *F. oxysporum* (Fo47) at 2 and 3 weeks post‐inoculation (Validov et al. 2011). On the contrary, Aime et al. showed that the kinetics of tomato root colonisation by Fo47 and Fol8 were the same (Aimé et al. 2013).

In addition to differences in fungal biomass, the root colonisation pattern varies between *F. oxysporum* pathogens and endophytic strains. Our results indicate the presence of both strains in flax shoots, which differs from the literature data, which indicate that pathogenic strains typically exhibit the ability to invade xylem vessels, enabling systemic colonisation of above‐ground tissues (de Lamo and Takken 2020), while Fo47 colonisation in pea is restricted to the root surface and the outermost cortical cell layers; the pathogenic *F. oxysporum* f. sp. *pisi* aggressively penetrates deeper root tissues, including the vascular system (Benhamou and Garand 2001). Similarly, in tomato, both Fo47 and *F. oxysporum* f. sp. *lycopersici* initially colonise the root surface, subsequently progressing toward the elongation zone and root tip. However, only the pathogenic strain extensively invades deeper root tissues and ultimately reaches the vascular system, whereas Fo47 remains confined to the epidermis and cortex (Nahalkova et al. 2008). Importantly, penetration of root tissues can be limited by activating the plant antioxidant system in living perivascular cells, limiting oxidative damage within them, thus preventing the spread of infection into the xylem (Darino et al. 2022). Contrarily, Fo47 has been reported to colonise the xylem vessels of eucalyptus (Salerno et al. 2000), while the endophytic strain Fo CS‐20 has been detected in the xylem of cucumber (Pu et al. 2014). Hyphae of Fo47 have also been observed within the vasculature of *Arabidopsis thaliana* and tomato roots, though their presence was restricted to the vasculature of lateral roots and the elongation zone of primary roots. In contrast, only pathogenic strains successfully colonised the xylem above the primary root maturation zone (Martínez‐Soto et al. 2023). Extensive colonisation of the root cortex and vascular system is a hallmark of pathogenic strains, and this capacity is associated with increased secretion of cell wall‐degrading enzymes (Jonkers et al. 2009).

Plant priming was conducted by first introducing *F. oxysporum* Fo47 spores into the substrate, followed by the addition of *F. oxysporum* Foln spores 4 days later. At the time of Foln inoculation, Fo47 hyphae were already present in the plant roots, which reduced Foln penetration and colonisation. In both roots and shoots of primed plants, Foln abundance was significantly lower compared to plants treated solely with Foln. Interestingly, the simultaneous inoculation of both fungal strains also led to a reduction in Foln levels compared to plants treated only with Foln. However, Foln abundance remained higher than in primed plants. This suggests competitive host colonisation, where in primed plants, the earlier establishment of Fo47 may have restricted Foln invasion (Zhu et al. 2022). Competition for space or nutrients at the root surface is also hypothesised to be a primary mechanism of action for the non‐pathogenic *F. oxysporum* strain F2 against *Verticillium dahliae* in eggplants, as F2 application was found to reduce *V. dahliae* DNA levels in plants (Pantelides et al. 2009).

A similar relationship has been observed in rice, where pre‐inoculation with *P. liquidambaris* B3 facilitates endophyte colonisation while significantly reducing the colonisation of the pathogen *Fusarium proliferatum*. However, in the case of co‐inoculation with both the endophyte and the pathogen, a significant increase in pathogen growth and a reduction in endophyte abundance were observed (Sun et al. 2021; Zhu et al. 2022). The colonisation dynamics of endophytic and pathogenic fungi also depend on plant resistance. Studies have shown that less resistant plants are more susceptible to colonisation by pathogenic strains. However, priming with non‐pathogenic strains can limit pathogen spread. This effect was observed in *Ulmus minor* infected with *Ophiostoma novo‐ulmi* , where prior sensitization with various fungal endophytes restricted pathogen proliferation (Martínez‐Arias et al. 2021).

On the other hand, the local suppression effects against *F. oxysporum* by *P. liquidambaris* B3 were stronger than the systemic effects (local: 0.56‐fold compared to systemic: 0.69‐fold), likely reflecting that B3 hyphae colonising peanut roots can restrict further pathogen colonisation through competition for intercellular niches and/or the formation of a physicochemical barrier (Sun et al. 2021). Our study presents similar findings, demonstrating that in primed plants, Fo47 locally suppresses Foln colonisation in roots by 92%, while systemically reducing its presence in shoots by 87%. Moreover, when plants were treated with both strains simultaneously, Fo47 reduced Foln colonisation locally by 50% but did not inhibit its systemic spread.

In plants treated separately with Fo47 and Foln, the mRNA levels of *chitinase* increase over time, beginning at 2 dptN/P. However, from 4 dptN/P onward, plants treated with Foln exhibit higher transcript levels than those treated with Fo47 or co‐inoculated with both strains. These results align with transcriptomic studies in tomato plants inoculated with the virulent *F. oxysporum* f. sp. *lycopersici* and the biocontrol strain *F. oxysporum* Fo47, where the mRNA levels of two *chitinase* isoforms and two *β‐glucanase* isoforms were lower in the initial hours but significantly higher in the later hours in plants infected with the pathogenic strain compared to those treated with the non‐pathogenic strain (Aimé et al. 2008; Aimé et al. 2013). This suggests that in primed plants, at the time of Foln inoculation, *chitinase* transcript levels were already significantly elevated compared to control plants. These differences are particularly evident at 2 dptP and 4 dptP, while from 6 dptP onward, *chitinase* transcript levels become significantly higher in Foln‐treated plants compared to primed plants. In conclusion, the priming effect of Fo47 is most pronounced between 2 and 4 dptP. A similar trend to that observed with *chitinase* transcripts was also observed for *β‐glucanase* transcripts. Literature data also demonstrate a similar relationship for plants primed with Fo47 (Aimé et al. 2013).

ROS analysis showed that the initial increase in H_2_O_2_ occurs around 2 dptP in Foln‐treated plants and around 6 dptN in Fo47‐treated plants, followed by a second increase approximately 14 days after treatment with either Foln or Fo47. Similar early H_2_O_2_ fluctuations were reported in flax cell suspensions, where both Foln3 and Fo47 triggered transient H_2_O_2_ production within the first few minutes of interaction, but the non‐pathogenic strain also induced a second oxidative burst 3 h post‐inoculation resulted in a higher number of dead cells in the Fo47 treatment (Olivain et al. 2003). Interestingly, in primed plants, two H_2_O_2_ peaks were detected in roots at 2 and 14 dptP, similar to Foln‐treated roots. This pattern suggests a typical two‐phase oxidative burst, where the first peak likely serves as a signalling or priming event preparing the cells for defence, however further investigation is needed. The second, stronger peak may correspond to the induction of cell death through intensified ROS production in organelles such as mitochondria and chloroplasts (Van Aken and Van Breusegem 2015). A comparable pattern of early H_2_O_2_ accumulation at the initial stage of colonisation was confirmed in peanuts, where both shoots and roots of B3‐preinoculated plants exhibited increased H_2_O_2_ production upon Fo infection, similar to plants treated solely with Fo (Sun et al. 2021). In rice, an increase in H_2_O_2_ was also detected at 2 dptP, though it was significantly lower in roots and shoots of B3‐preinoculated plants than in co‐inoculated (B3 + *F. proliferatum*) or *F. proliferatum*‐treated plants (Zhu et al. 2022). A similar trend was observed in chickpea, where *F. oxysporum* f. sp. *ciceris* (Foc) treatment alone caused a higher increase in H_2_O_2_ content than in plants pre‐primed with *Streptomyces araujoniae* (TN). In these plants, no significant differences were observed in SOD and CAT activity between Foc‐treated and Foc + TN‐treated plants, despite a marked increase in SOD activity compared to untreated plants (Zeyad et al. 2022). An increase in SOD activity was also reported in eggplant, with a more pronounced effect in plants primed with *Aspergillus terreus* prior to *Alternaria solani* infection than in plants treated solely with the pathogen. However, CAT activity was comparable between infected and primed plants (Attia et al. 2022). In *Solanum lycopersicum* infected with *F. oxysporum* f. sp. *lycopersici*, the initial activation of the antioxidant system (increased phenolic compound content, increased H_2_O_2_ concentration, as well as increased activity of antioxidant enzymes: SOD, CAT, GPX, and APX) weakened over time (in particular, a decrease in CAT activity), indicating an insufficiently strong antioxidant system to hinder the pathogen's penetration and spread into the host (Mandal et al. 2008). However, our research indicates that treatment of flax with Fo47 induces an antioxidant system in these plants, which, following infection with the pathogenic Foln, acts faster and more efficiently not only locally but also systemically than in plants infected only with Foln.

The increase in H_2_O_2_ levels in the roots of Fo47‐treated plants correlates with elevated catalase activity at 6 and 14 dptN and increased superoxide dismutase activity at 18 dptN. The rise in H_2_O_2_ at 6 dptN may result from an upregulation of *NADPH oxidase D* transcripts at 4 dptN, while subsequent increases in *NADPHox F*, *NADPHox C*, *sodMn*, and *sodFe* transcripts at 8 dptN, along with a decrease in *cat* mRNA levels at 10 dptN, support maintain ROS homeostasis during these time points. Additionally, the upregulation of *NADPHox C* and the downregulation of *cat* at 14 dptN, followed by increased *sodCu* and *apx* mRNA levels at 18 dptN, likely contribute to counteracting oxidative stress and balancing H_2_O_2_ levels. In the shoots of Fo47‐treated plants, no significant changes in antioxidant enzyme activities were observed. However, variations were detected in the transcript levels of these genes. A marked decrease in *NADPHox F* transcript levels was evident as early as 2 dptN. From 8 to 18 dptN, an increase in *NADPHox D* transcript levels was observed, alongside an initial decline followed by a subsequent rise in *sod* and *apx* transcript levels.

The correlations observed in the roots of Fo47‐treated plants were not present in Foln‐treated roots, where only SOD activity increased at 4 and 14 dptP. Transcript analysis of ROS metabolism‐related genes revealed temporal fluctuations, with a pronounced upregulation of all analysed transcripts at 14 dptP. In the shoots of Foln‐treated plants, no significant changes in antioxidant enzyme activities were detected. However, variations in transcript levels were observed, including an increase in *NADPHox D* at 2 dptP, a decrease in *sodCu* and *apx* at 6 dptP, and an overall upregulation of most analysed transcripts at 14 dptP.

Previous studies examining changes in the transcript levels of genes encoding NADPH oxidases indicate that the NADPH oxidases: RBOHD and RBOHF play the most important role in plant defence processes during colonisation by pathogenic and non‐pathogenic fungi. Their mRNA levels are increased in *Arabidopsis* plants infected with *F. oxysporum* (Zhu et al. 2013; Lyons et al. 2015). Additionally, analyses of the *rbohD* and *rbohF* mutants showed significantly increased and decreased disease resistance in *Arabidopsis* to *F. oxysporum*, respectively, suggesting that these two NADPH oxidase‐encoding genes have opposing effects on disease development (Zhu et al. 2013). Furthermore, Guo et al. demonstrated that non‐pathogenic Fo47 inhibited RBOHD expression in *Arabidopsis*, allowing it to efficiently colonise the root, confirming that RBOHD normally functions to limit colonisation (Guo et al. 2021).

Interestingly, the activities of the analysed enzymes do not change in the roots of primed plants, except for a catalase activity increase at 4 dptP in roots of plants treated simultaneously with both strains. In these roots, upregulation of ROS metabolism‐related gene transcripts was observed at 2, 4, and 14 dptP, whereas downregulation occurred at 6 dptP. In the shoots of primed plants, the observed increase in H_2_O_2_ levels at 4 dptP was preceded by a rise in catalase activity at 2 dptP, along with increased transcript levels of ROS metabolism‐related genes (*NADPHox D*, *NADPHox F*, *sodCu*, *cat*, and *apx*) compared to untreated plants and those treated with either Fo47 or Foln alone. This suggests a systemic response (observed in the shoots) to Foln infection following Fo47 priming.

A similar pattern is observed in the shoots of plants treated with both strains simultaneously, with the difference occurring at 4 dptP/N. In this case, a systemic plant response to Foln infection concurrent with Fo47 colonisation can also be inferred. Moreover, these changes are absent in plants treated solely with Foln, suggesting that this systemic response likely contributes to enhanced plant resistance and a reduction in Foln infection (i.e., decreased fungal biomass in the plant), ultimately reflected in the plant phenotype. In Fo47‐primed plants, a more rapid systemic response to Foln infection is observed compared to plants co‐treated with Foln and Fo47, which correlates with faster colonisation by Fo47. It can be speculated that plant resistance enhancement through systemic response may involve strengthening the cell wall structure via lignification and callose deposition. However, further research is required to confirm this hypothesis.

During the early stages of plant colonisation by pathogens and endophytes, changes in H_2_O_2_ levels serve as a rapid signal for the activation of antioxidant defence responses. However, at later stages (14 dptP), a significant increase in H_2_O_2_ content may be associated with disease symptoms and programmed cell death (PCD) (Sahu et al. 2022). Nevertheless, this hypothesis requires further validation and additional studies.

The enzymatic antioxidant system appears to be more effective and important during pathogen infections, but the non‐enzymatic antioxidant system is necessary for support and supplementation. Analysis of phenolic compounds revealed a greater contribution of the non‐enzymatic antioxidant system in shoots than in roots, through increased ferulic acid and *p*‐coumaric acid content in plants treated with Fo47 at early stages of colonisation, and increased vanillin content in plants treated with Fo47, Foln, and plants primed and then treated with Foln at later stages of colonisation. Increased ferulic acid content and other phenolic acids (caffeic acid and vanillic acid) were also demonstrated in tomato roots and shoots treated with pathogenic and non‐pathogenic strains of *F. oxysporum* (Panina et al. 2007; Khallal 2007). An additional parameter confirming the importance of phenolic compounds as non‐enzymatic antioxidants was antioxidant potential, which was higher in roots during the initial days of fungal colonisation and later in shoots, with the highest levels in plants treated solely with Foln in most cases. Our results are consistent with those for *Asparagus officinalis* L. primed with non‐pathogenic *F. oxysporum* (NPFO) and then infected with *F. oxysporum* f. sp. *asparagi*, where treatment with the non‐pathogen induced an increase in total polyphenols and antioxidant potential, and additional infection of the primed plant with pathogenic *F. oxysporum* further increased these parameters (Liu and Matsubara 2016).

**FIGURE 7:**
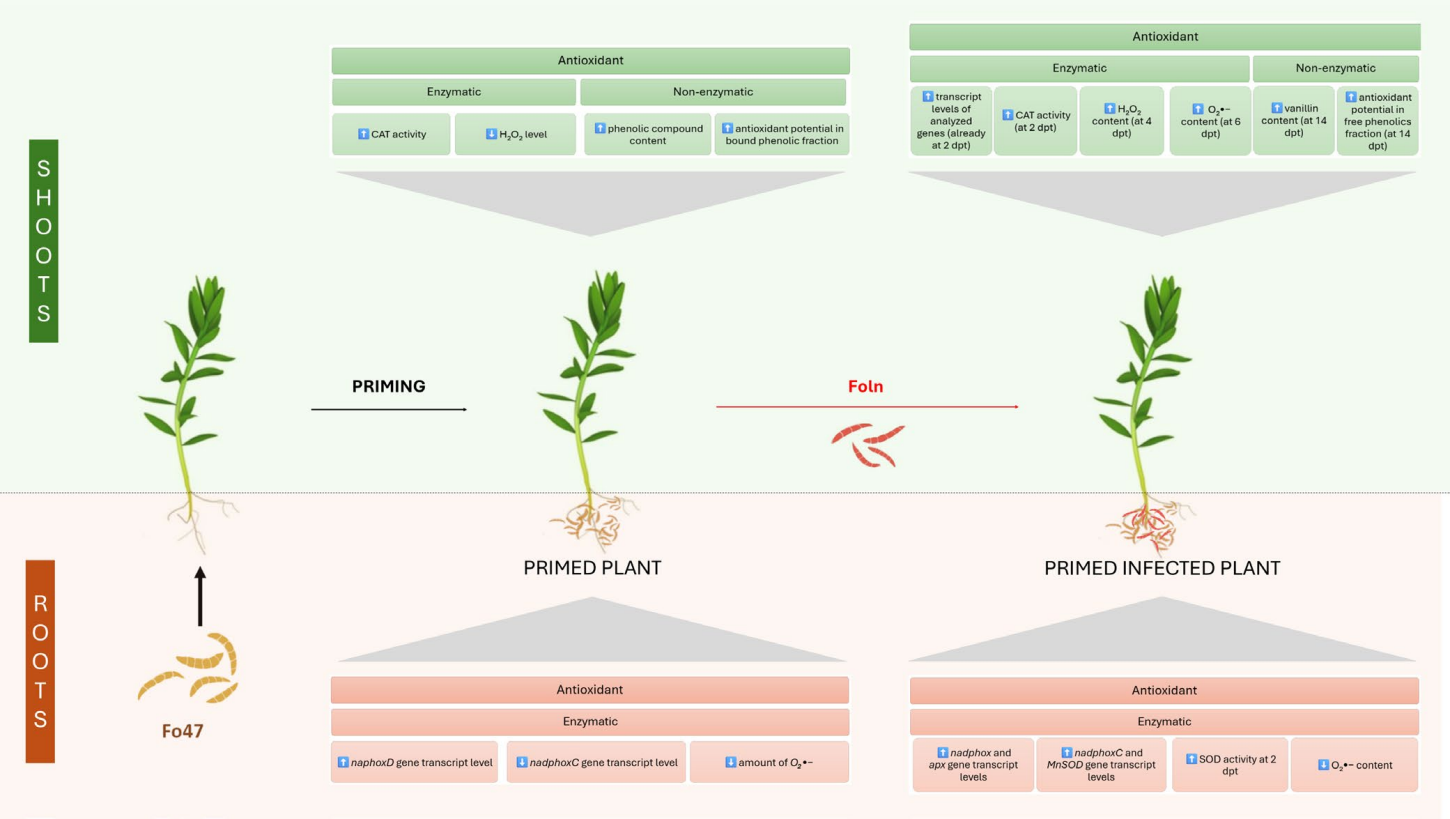
Plant priming based on enzymatic and non‐enzymatic antioxidant system. Changes in the enzymatic (ROS metabolism‐related genes expression, catalase and superoxide dismutase activity, and hydrogen peroxide and superoxide anion levels) and non‐enzymatic (phenolic compound content and antioxidant potential) antioxidant systems occurring in the roots and shoots of plants primed with Fo47 and plants primed with Fo47 and infected with Foln.

In summary, flax plants treated with the non‐pathogenic Fo47 strain are primed for infection by *F. oxysporum* f. sp. *lini* by increasing their immune system. Non‐pathogenic Fo47 colonises host roots and activates the antioxidant system: enzymatic locally in the roots and enzymatic and non‐enzymatic systemically in the shoots (Figure 7). The consequence of this is that in plants primed by Fo47 and then infected with Foln, the colonisation of tissues by the pathogen is limited by: the occupation of the niche by Fo47 and a prepared/active antioxidant system that can inhibit the penetration of the pathogen into the xylem.

This study demonstrated that the non‐pathogenic *F. oxysporum* strain can be an effective agricultural strategy for controlling or mitigating flax disease caused by the pathogenic *F. oxysporum* strain. Furthermore, it suggests that the appropriate application of beneficial microorganisms could become a key factor in the management of crop diseases.

## Author Contributions

**Marta Burgberger:** investigation, visualization, writing – review and editing, writing – original draft, formal analysis. **Justyna Mierziak:** investigation, methodology, writing – review and editing, writing – original draft, formal analysis. **Wioleta Wojtasik:** conceptualization, funding acquisition, writing – original draft, writing – review and editing, supervision, visualization, project administration, methodology.

## Funding

This work was supported by Narodowe Centrum Nauki (2019/33/B/NZ9/00408).

## Conflicts of Interest

The authors declare no conflicts of interest.

## Supporting information


**Figure S1:** (A) The fragment of the genome of Fo47 or Foln sequence obtained after the PCR reaction in roots and shoots of plants primed with non‐pathogenic strain Fo47 and plants treated with both strains simultaneously after 0, 2, 4, 6 and 14 days of Foln treatment. The amplified Fo47 genome fragment was 68 bp shorter than the Foln genome fragment. PC—positive control; NC—negative control. (B) Relative quantity of fungal *murein transglycosylase* gene in roots and shoots of plants primed with non‐pathogenic strain Fo47 and plants treated with both strains simultaneously. The data presented were obtained from real‐time PCR analysis. Flax *actin* served as the reference gene. The data represent the mean ± standard deviations from three independent experiments The significance of differences between groups was determined using a one‐way ANOVA, followed by Fisher's post hoc test. Differences were considered statistically significant when *p* < 0.05 (* for comparison to control, non‐treated plants from the same time point as the sample; ▲for comparison of plants primed with non‐pathogenic strain Fo47 or plants treated with both strains simultaneously with Foln treated plants from the same time point as sample). (C) Number of copies of the six7 gene of pathogenic *Fusarium oxysporum* strain in the root and shoot of flax plants primed with the non‐pathogenic strain Fo47 and plants treated with both strains of the fungus simultaneously after 2, 4, 6 and 14 days of Foln treatment. The data represent the mean ± standard deviations from three independent experiments The significance of differences between groups was determined using a one‐way ANOVA, followed by Fisher's post hoc test. Differences were considered statistically significant when *p* < 0.05 (* for comparison to control, non‐treated plants from the same time point as the sample; ▲for comparison of plants primed with non‐pathogenic strain Fo47 or plants treated with both strains simultaneously with Foln treated plants from the same time point as sample).

**Figure S2:** Transcript levels of *PR genes* and genes involved in ROS metabolism in roots and shoots of plants primed with non‐pathogenic strain Fo47 and plants treated with both strains simultaneously. Changes in transcript levels of *PR genes* (*chitinase* and *β‐glucanase*) and ROS metabolism genes (*NADPH oxidase D, NADPH oxidase F*, and *NADPH oxidase C*, three isoforms of *superoxide dismutase* (*sod*): *sodCu/Zn*, *sodMn*, and *sodFe*, *catalase* and *ascorbate peroxidase*) are shown as relative quantity (RQ) to the reference gene (*actin*) for control. Results were obtained by real‐time PCR on a cDNA template and are presented as mean ± SD (*n* = 3). The significance of differences between groups was determined using a one‐way ANOVA, followed by Fisher's post hoc test. Differences were considered statistically significant when *p* < 0.05 (* for comparison to control, non‐treated plants from the same time point as the sample; ▲for comparison of plants primed with non‐pathogenic strain Fo47 or plants treated with both strains simultaneously with Foln treated plants from the same time point as sample).

**Figure S3:** Catalase and superoxide dismutase activity and H_2_O_2_ and O_2_^−^ content in roots and shoots of plants primed with non‐pathogenic strain Fo47 and plants treated with both strains simultaneously. Bars represent the mean ± SD from three replicates. The significance of differences between groups was determined using a one‐way ANOVA, followed by Fisher's post hoc test. Differences were considered statistically significant when *p* < 0.05 (* for comparison to control, non‐treated plants from the same time point as the sample; ▲for comparison of plants primed with non‐pathogenic strain Fo47 or plants treated with both strains simultaneously with Foln treated plants from the same time point as sample).

**Figure S4:** Antioxidant potential in extract including free phenolic compounds and in extract bound phenolic compounds from roots and shoots of plants primed with non‐pathogenic strain of *Fusarium oxysporum* and plants treated with both strains of *Fusarium oxysporum* simultaneously. The significance of differences between groups was determined using a one‐way ANOVA, followed by Fisher's post hoc test. Differences were considered statistically significant when *p* < 0.05 (* for comparison to control, non‐treated plants from the same time point as the sample; ►◄ for comparison of plants primed with non‐pathogenic strain Fo47 or plants treated with both strains simultaneously with Foln treated plants from the same time point as sample).

**Table S1:** Sequences of primers used for the PCR reaction.

**Table S2:** Phenolic compound content (vanillin, *trans*‐ferulic acid and *p*‐coumaric acid) and antioxidant potential (in extract including free phenolic compounds and in extract bound phenolic compounds) from roots and shoots of plants primed with non‐pathogenic strain of *Fusarium oxysporum* and plants treated with both strains of *Fusarium oxysporum* simultaneously. The significance of differences between groups was determined using a one‐way ANOVA, followed by Fisher's post hoc test. Differences were considered statistically significant when *p* < 0.05 (* for comparison to control, non‐treated plants from the same time point as the sample; ►◄ for comparison of plants primed with non‐pathogenic strain Fo47 or plants treated with both strains simultaneously with Foln treated plants from the same time point as sample).

## Data Availability

All study data that support the findings of this study are included in the article and Supporting Information. All raw data are available from the corresponding author upon reasonable request.

## References

[emi470263-bib-0001] Aebi, H. 1984. “Catalase In Vitro.” In Methods in Enzymology, 121–126. Academic Press. 10.1016/s0076-6879(84)05016-3 6727660

[emi470263-bib-0002] Aimé, S. , C. Alabouvette , C. Steinberg , and C. Olivain . 2013. “The Endophytic Strain *Fusarium oxysporum* Fo47: A Good Candidate for Priming the Defense Responses in Tomato Roots.” Molecular Plant‐Microbe Interactions 26: 918–926. 23617416 10.1094/MPMI-12-12-0290-R

[emi470263-bib-0003] Aimé, S. , C. Cordier , C. Alabouvette , and C. Olivain . 2008. “Comparative Analysis of PR Gene Expression in Tomato Inoculated With Virulent *Fusarium oxysporum* f. sp. Lycopersici and the Biocontrol Strain *F. oxysporum* Fo47.” Physiological and Molecular Plant Pathology 73: 9–15.

[emi470263-bib-0004] Alabouvette, C. , D. la De broise , P. Lemanceau , Y. Couteaudier , and J. Louvet . 1987. “Utilisation de souches non pathogènes de Fusarium pour lutter contre les fusarioses: situation actuelle dans la pratique.” EPPO Bulletin 17: 665–674.

[emi470263-bib-0005] Apel, K. , and H. Hirt . 2004. “Reactive Oxygen Species: Metabolism, Oxidative Stress, and Signal Transduction.” Annual Review of Plant Biology 55: 373–399. 10.1146/annurev.arplant.55.031903.141701 15377225

[emi470263-bib-0006] Attia, M. S. , A. H. Hashem , A. A. Badawy , and A. M. Abdelaziz . 2022. “Biocontrol of Early Blight Disease of Eggplant Using Endophytic Aspergillus Terreus: Improving Plant Immunological, Physiological and Antifungal Activities.” Botanical Studies 63: 26. 36030517 10.1186/s40529-022-00357-6 PMC9420682

[emi470263-bib-0007] Baker, A. , C.‐C. Lin , C. Lett , B. Karpinska , M. H. Wright , and C. H. Foyer . 2023. “Catalase: A Critical Node in the Regulation of Cell Fate.” Free Radical Biology and Medicine 199: 56–66. 36775107 10.1016/j.freeradbiomed.2023.02.009

[emi470263-bib-0008] Benhamou, N. , and C. Garand . 2001. “Cytological Analysis of Defense‐Related Mechanisms Induced in Pea Root Tissues in Response to Colonization by Nonpathogenic *Fusarium oxysporum* Fo47.” Phytopathology 91: 730–740. 18944029 10.1094/PHYTO.2001.91.8.730

[emi470263-bib-0009] Blokhina, O. , E. Virolainen , and K.v. Fagerstedt . 2003. “Antioxidants, Oxidative Damage and Oxygen Deprivation Stress: A Review.” Annals of Botany 91: 179–194. 12509339 10.1093/aob/mcf118 PMC4244988

[emi470263-bib-0010] Bolwerk, A. , A. L. Lagopodi , B. J. Lugtenberg , and G. V. Bloemberg . 2005. “Visualization of Interactions Between a Pathogenic and a Beneficial Fusarium Strain During Biocontrol of Tomato Foot and Root Rot.” Molecular Plant‐Microbe Interactions 18: 710–721. 16042017 10.1094/MPMI-18-0710

[emi470263-bib-0011] Camejo, D. , Á. Guzmán‐Cedeño , and A. Moreno . 2016. “Reactive Oxygen Species, Essential Molecules, During Plant‐Pathogen Interactions.” Plant Physiology and Biochemistry 103: 10–23. 26950921 10.1016/j.plaphy.2016.02.035

[emi470263-bib-0012] Constantin, M. E. , F. J. de Lamo , B. V. Vlieger , M. Rep , and F. L. W. Takken . 2019. “Endophyte‐Mediated Resistance in Tomato to *Fusarium oxysporum* Is Independent of ET, JA, and SA.” Frontiers in Plant Science 10: 979. 31417594 10.3389/fpls.2019.00979 PMC6685397

[emi470263-bib-0013] Darino, M. , K. Kanyuka , and K. E. Hammond‐Kosack . 2022. “Apoplastic and Vascular Defences.” Essays in Biochemistry 66: 595–605. 36062526 10.1042/EBC20220159

[emi470263-bib-0014] de Lamo, F. J. , and F. L. W. Takken . 2020. “Biocontrol by *Fusarium oxysporum* Using Endophyte‐Mediated Resistance.” Frontiers in Plant Science 11: 37. 32117376 10.3389/fpls.2020.00037 PMC7015898

[emi470263-bib-0015] Dean, R. , J. A. Van Kan , Z. A. Pretorius , et al. 2012. “The Top 10 Fungal Pathogens in Molecular Plant Pathology.” Molecular Plant Pathology 13: 414–430. 22471698 10.1111/j.1364-3703.2011.00783.x PMC6638784

[emi470263-bib-0016] Duijff, B. J. , G. Recorbet , P. A. Bakker , J. E. Loper , and P. Lemanceau . 1999. “Microbial Antagonism at the Root Level Is Involved in the Suppression of Fusarium Wilt by the Combination of Nonpathogenic *Fusarium oxysporum* Fo47 and *Pseudomonas putida* WCS358.” Phytopathology 89: 1073–1079. 18944664 10.1094/PHYTO.1999.89.11.1073

[emi470263-bib-0017] Gordon, T. R. 2017. “ *Fusarium oxysporum* and the Fusarium Wilt Syndrome.” Annual Review of Phytopathology 55: 23–39. 10.1146/annurev-phyto-080615-095919 28489498

[emi470263-bib-0018] Guo, L. , H. Yu , B. Wang , et al. 2021. “Metatranscriptomic Comparison of Endophytic and Pathogenic Fusarium–Arabidopsis Interactions Reveals Plant Transcriptional Plasticity.” Molecular Plant‐Microbe Interactions 34: 1071–1083. 33856230 10.1094/MPMI-03-21-0063-R PMC9048145

[emi470263-bib-0019] He, C. Y. , and D. J. Wolyn . 2005. “Potential Role for Salicylic Acid in Induced Resistance of Asparagus Roots to *Fusarium oxysporum* f.sp. Asparagi.” Plant Pathology 54: 227–232.

[emi470263-bib-0020] Hu, C.‐H. , P.‐Q. Wang , P.‐P. Zhang , et al. 2020. “NADPH Oxidases: The Vital Performers and Center Hubs During Plant Growth and Signaling.” Cells 9: 437. 32069961 10.3390/cells9020437 PMC7072856

[emi470263-bib-0021] Jonkers, W. , C. D. Rodrigues , and M. Rep . 2009. “Impaired Colonization and Infection of Tomato Roots by the Deltafrp1 Mutant of *Fusarium oxysporum* Correlates With Reduced CWDE Gene Expression.” Molecular Plant‐Microbe Interactions 22: 507–518. 19348569 10.1094/MPMI-22-5-0507

[emi470263-bib-0022] Kaur, R. , and R. S. Singh . 2007. “Study of Induced Systemic Resistance in *Cicer arietinum* L. due to Nonpathogenic *Fusarium oxysporum* Using a Modified Split Root Technique.” 155: 694–698.

[emi470263-bib-0023] Khallal, S. M. E. 2007. Induction and modulation of resistance in tomato plants against Fusarium wilt disease by bioagent fungi (arbuscular mycorrhiza) and/or hormonal elicitors (Jasmonic acid & Salicylic acid): 2‐Changes in the antioxidant enzymes, phenolic compounds and pathogen related‐ proteins.

[emi470263-bib-0024] Kunos, V. , M. Cséplő , D. Seress , et al. 2022. “The Stimulation of Superoxide Dismutase Enzyme Activity and Its Relation With the Pyrenophora teres f. teres Infection in Different Barley Genotypes.” Sustainability 14: 2597.

[emi470263-bib-0025] Lämke, J. , and I. Bäurle . 2017. “Epigenetic and Chromatin‐Based Mechanisms in Environmental Stress Adaptation and Stress Memory in Plants.” Genome Biology 18: 124. 28655328 10.1186/s13059-017-1263-6 PMC5488299

[emi470263-bib-0026] Li, C. , C. Zuo , G. Deng , et al. 2013. “Contamination of Bananas With Beauvericin and Fusaric Acid Produced by *Fusarium oxysporum* f. sp. Cubense.” PLoS One 8: e70226. 23922960 10.1371/journal.pone.0070226 PMC3724834

[emi470263-bib-0027] Li, S. 2023. “Novel Insight Into Functions of Ascorbate Peroxidase in Higher Plants: More Than a Simple Antioxidant Enzyme.” Redox Biology 64: 102789. 37352686 10.1016/j.redox.2023.102789 PMC10333675

[emi470263-bib-0028] Liu, J. , and Y.‐i. Matsubara . 2016. “Induced Systemic Resistance to Fusarium Root Rot and Changes in Antioxidative Ability by Arbuscular Mycorrhizal Fungus and Non‐Pathogenic *Fusarium oxysporum* in Asparagus Plants.” Journal of the Japanese Society of Agricultural Technology Management 23: 21–29.

[emi470263-bib-0029] Lyons, R. , J. Stiller , J. Powell , A. Rusu , J. M. Manners , and K. Kazan . 2015. “ *Fusarium oxysporum* Triggers Tissue‐Specific Transcriptional Reprogramming in *Arabidopsis thaliana* .” PLoS One 10: e0121902. 25849296 10.1371/journal.pone.0121902 PMC4388846

[emi470263-bib-0030] Magan, N. 2007. “Fusarium Mycotoxins: Chemistry, Genetics and Biology, by A. E. Desjardins. 260 Pp. St Paul, MN, USA: American Phytopathology Society (2006). US$89 (Hardback). ISBN 0‐89054‐335‐6.” Journal of Agricultural Science 145: 539.

[emi470263-bib-0031] Mandal, S. , A. Mitra , and N. Mallick . 2008. “Biochemical Characterization of Oxidative Burst During Interaction Between *Solanum lycopersicum* and *Fusarium oxysporum* f. sp. Lycopersici.” Physiological and Molecular Plant Pathology 72: 56–61.

[emi470263-bib-0032] Martínez‐Arias, C. , J. Sobrino‐Plata , L. Gil , J. Rodríguez‐Calcerrada , and J. A. Martín . 2021. “Priming of Plant Defenses Against *Ophiostoma Novo‐ulmi* by Elm (*Ulmus minor* Mill.) Fungal Endophytes.” Journal of Fungi 7: 687. 34575725 10.3390/jof7090687 PMC8469682

[emi470263-bib-0033] Martínez‐Soto, D. , H. Yu , K. S. Allen , and L. J. Ma . 2023. “Differential Colonization of the Plant Vasculature Between Endophytic Versus Pathogenic *Fusarium oxysporum* Strains.” Molecular Plant‐Microbe Interactions 36: 4–13. 36279112 10.1094/MPMI-08-22-0166-SC PMC10052776

[emi470263-bib-0034] Mirocha, C. J. , H. K. Abbas , T. Kommedahl , and B. B. Jarvis . 1989. “Mycotoxin Production by *Fusarium oxysporum* and Fusarium Sporotrichioides Isolated From Baccharis spp. From Brazil.” Applied and Environmental Microbiology 55: 254–255. 2705770 10.1128/aem.55.1.254-255.1989 PMC184088

[emi470263-bib-0035] Moročko‐Bičevska, I. , J. Fatehi , and B. Gerhardson . 2014. “Biocontrol of Strawberry Root Rot and Petiole Blight by Use of Non‐Pathogenic Fusarium sp. Strains.” Acta Horticulturae 1049: 599–605.

[emi470263-bib-0036] Mulero‐Aparicio, A. , C. Agustí‐Brisach , Á. Varo , F. J. López‐Escudero , and A. Trapero . 2019. “A Non‐Pathogenic Strain of *Fusarium oxysporum* as a Potential Biocontrol Agent Against Verticillium Wilt of Olive.” Biological Control 139: 104045.

[emi470263-bib-0037] Nahalkova, J. , J. Fatehi , C. Olivain , and C. Alabouvette . 2008. “Tomato Root Colonization by Fluorescent‐Tagged Pathogenic and Protective Strains of *Fusarium oxysporum* in Hydroponic Culture Differs From Root Colonization in Soil.” FEMS Microbiology Letters 286: 152–157. 18657114 10.1111/j.1574-6968.2008.01241.x

[emi470263-bib-0038] Nouayti, F. , I. Madani , T. Abdessalem , B. Abdelali , and R. Lahlali . 2018. “Ability of Non‐Pathogenic *Fusarium oxysporum* Strain Fo47 to Suppress Rhizomania Disease of Sugar Beets in Morocco.” Notulae Scientia Biologicae 10: 137–142.

[emi470263-bib-0039] O'Brien, J. A. , A. Daudi , V. S. Butt , and G. P. Bolwell . 2012. “Reactive Oxygen Species and Their Role in Plant Defence and Cell Wall Metabolism.” Planta 236: 765–779. 22767200 10.1007/s00425-012-1696-9

[emi470263-bib-0040] Olivain, C. , S. Trouvelot , M. N. Binet , C. Cordier , A. Pugin , and C. Alabouvette . 2003. “Colonization of Flax Roots and Early Physiological Responses of Flax Cells Inoculated With Pathogenic and Nonpathogenic Strains of *Fusarium oxysporum* .” Applied and Environmental Microbiology 69: 5453–5462. 12957934 10.1128/AEM.69.9.5453-5462.2003 PMC194917

[emi470263-bib-0041] Palma, J. M. , R. M. Mateos , J. López‐Jaramillo , et al. 2020. “Plant Catalases as NO and H(2)S Targets.” Redox Biology 34: 101525. 32505768 10.1016/j.redox.2020.101525 PMC7276441

[emi470263-bib-0042] Panina, Y. , D. R. Fravel , C. J. Baker , and L. A. Shcherbakova . 2007. “Biocontrol and Plant Pathogenic *Fusarium oxysporum*‐Induced Changes in Phenolic Compounds in Tomato Leaves and Roots.” Journal of Phytopathology 155: 475–481.

[emi470263-bib-0043] Pantelides, I. S. , S. E. Tjamos , I. A. Striglis , I. Chatzipavlidis , and E. J. Paplomatas . 2009. “Mode of Action of a Non‐Pathogenic *Fusarium oxysporum* Strain Against *Verticillium dahliae* Using Real Time QPCR Analysis and Biomarker Transformation.” Biological Control 50: 30–36.

[emi470263-bib-0044] Planchon, A. , G. Durambur , J. B. Besnier , et al. 2021. “Effect of a *Bacillus subtilis* Strain on Flax Protection Against *Fusarium oxysporum* and Its Impact on the Root and Stem Cell Walls.” Plant, Cell & Environment 44: 304–322. 10.1111/pce.13882 32890441

[emi470263-bib-0045] Pu, X. , B. Xie , P. Li , et al. 2014. “Analysis of the Defence‐Related Mechanism in Cucumber Seedlings in Relation to Root Colonization by Nonpathogenic *Fusarium oxysporum* CS‐20.” FEMS Microbiology Letters 355: 142–151. 24810367 10.1111/1574-6968.12461

[emi470263-bib-0046] Racchi, M. L. 2013. “Antioxidant Defenses in Plants With Attention to Prunus and Citrus spp.” Antioxidants 2: 340–369. 26784469 10.3390/antiox2040340 PMC4665512

[emi470263-bib-0047] Rudenko, N. N. , D. V. Vetoshkina , T. V. Marenkova , and M. M. Borisova‐Mubarakshina . 2023. “Antioxidants of Non‐Enzymatic Nature: Their Function in Higher Plant Cells and the Ways of Boosting Their Biosynthesis.” Antioxidants (Basel) 12: 2014. 38001867 10.3390/antiox12112014 PMC10669185

[emi470263-bib-0048] Sahu, P. K. , K. Jayalakshmi , J. Tilgam , et al. 2022. “ROS Generated From Biotic Stress: Effects on Plants and Alleviation by Endophytic Microbes.” Frontiers in Plant Science 13: 1042936. 36352882 10.3389/fpls.2022.1042936 PMC9638130

[emi470263-bib-0049] Salerno, M. I. , S. Gianinazzi , and V. Gianinazzi‐Pearson . 2000. “Effects on Growth and Comparison of Root Tissue Colonization Patterns of *Eucalyptus viminalis* by Pathogenic and Nonpathogenic Strains of Fusarium Oxysporum.” New Phytologist 146: 317–324. 33862965 10.1046/j.1469-8137.2000.00629.x

[emi470263-bib-0050] Sood, M. 2025. “Reactive Oxygen Species (ROS): Plant Perspectives on Oxidative Signalling and Biotic Stress Response.” Discover Plants 2: 187.

[emi470263-bib-0051] Sun, K. , X.‐G. Xie , F. Lu , et al. 2021. “Peanut Preinoculation With a Root Endophyte Induces Plant Resistance to Soil‐Borne Pathogen *Fusarium oxysporum* via Activation of Salicylic Acid‐Dependent Signaling.” Plant and Soil 460: 297–312.

[emi470263-bib-0052] Tian, S. , R. Torres , A. R. Ballester , B. Li , L. Vilanova , and L. González‐Candelas . 2016. “Molecular Aspects in Pathogen‐Fruit Interactions: Virulence and Resistance.” Postharvest Biology and Technology 122: 11–21.

[emi470263-bib-0053] Trouvelot, S. , C. Olivain , G. Recorbet , Q. Migheli , and C. Alabouvette . 2002. “Recovery of *Fusarium oxysporum* Fo47 Mutants Affected in Their Biocontrol Activity After Transposition of the Fot1 Element.” Phytopathology 92: 936–945. 18944018 10.1094/PHYTO.2002.92.9.936

[emi470263-bib-0054] Validov, S. Z. , F. D. Kamilova , and B. J. Lugtenberg . 2011. “Monitoring of Pathogenic and Non‐Pathogenic *Fusarium oxysporum* Strains During Tomato Plant Infection.” Microbial Biotechnology 4: 82–88. 21255375 10.1111/j.1751-7915.2010.00214.x PMC3815798

[emi470263-bib-0055] Van Aken, O. , and F. Van Breusegem . 2015. “Licensed to Kill: Mitochondria, Chloroplasts, and Cell Death.” Trends in Plant Science 20: 754–766. 26442680 10.1016/j.tplants.2015.08.002

[emi470263-bib-0056] Veloso, J. , and J. Díaz . 2021. “The Non‐Pathogenic *Fusarium oxysporum* Fo47 Induces Distinct Responses in Two Closely Related Solanaceae Plants Against the Pathogen *Verticillium dahliae* .” Journal of Fungi 7: 344. 33925134 10.3390/jof7050344 PMC8146752

[emi470263-bib-0057] Wang, Y. , D. Ji , T. Chen , et al. 2019. “Production, Signaling, and Scavenging Mechanisms of Reactive Oxygen Species in Fruit–Pathogen Interactions.” International Journal of Molecular Sciences 20: 2994. 31248143 10.3390/ijms20122994 PMC6627859

[emi470263-bib-0058] Zeyad, M. T. , P. Tiwari , W. A. Ansari , et al. 2022. “Bio‐Priming With a Consortium of *Streptomyces araujoniae* Strains Modulates Defense Response in Chickpea Against Fusarium Wilt.” Frontiers in Microbiology 13: 998546. 36160196 10.3389/fmicb.2022.998546 PMC9493686

[emi470263-bib-0059] Zhang, J. , J. Chen , R. Jia , Q. Ma , Z. Zong , and Y. Wang . 2018. “Suppression of Plant Wilt Diseases by Nonpathogenic *Fusarium oxysporum* Fo47 Combined With Actinomycete Strains.” Biocontrol Science and Technology 28: 562–573.

[emi470263-bib-0060] Zhang, Q. , L. Yang , J. Zhang , et al. 2015. “Production of Anti‐Fungal Volatiles by Non‐Pathogenic *Fusarium oxysporum* and Its Efficacy in Suppression of Verticillium Wilt of Cotton.” Plant and Soil 392: 101–114.

[emi470263-bib-0061] Zhu, Q. , Y. B. Wu , M. Chen , et al. 2022. “Preinoculation With Endophytic Fungus *Phomopsis liquidambaris* Reduced Rice Bakanae Disease Caused by *Fusarium proliferatum* via Enhanced Plant Resistance.” Journal of Applied Microbiology 133: 1566–1580. 35686661 10.1111/jam.15656

[emi470263-bib-0062] Zhu, Q.‐H. , S. Stephen , K. Kazan , et al. 2013. “Characterization of the Defense Transcriptome Responsive to *Fusarium oxysporum*‐Infection in Arabidopsis Using RNA‐Seq.” Gene 512: 259–266. 23107761 10.1016/j.gene.2012.10.036

[emi470263-bib-0063] Zhu, Y. , H. Su , X.‐X. Liu , et al. 2024. “Identification of NADPH Oxidase Genes Crucial for Rice Multiple Disease Resistance and Yield Traits.” Rice 17: 1. 38170415 10.1186/s12284-023-00678-5 PMC10764683

