# Supplementary figures and images for: Systemic Activation of the Antioxidant System by Root Priming With Non‐Pathogenic Fusarium oxysporum in Flax Infected With Pathogenic Fusarium oxysporum
Source: Environ Microbiol Rep. 2026 Jan 8;18(1):e70263. doi: 10.1111/1758-2229.70263 (PMC12784107; doi:10.1111/1758-2229.70263)

A)


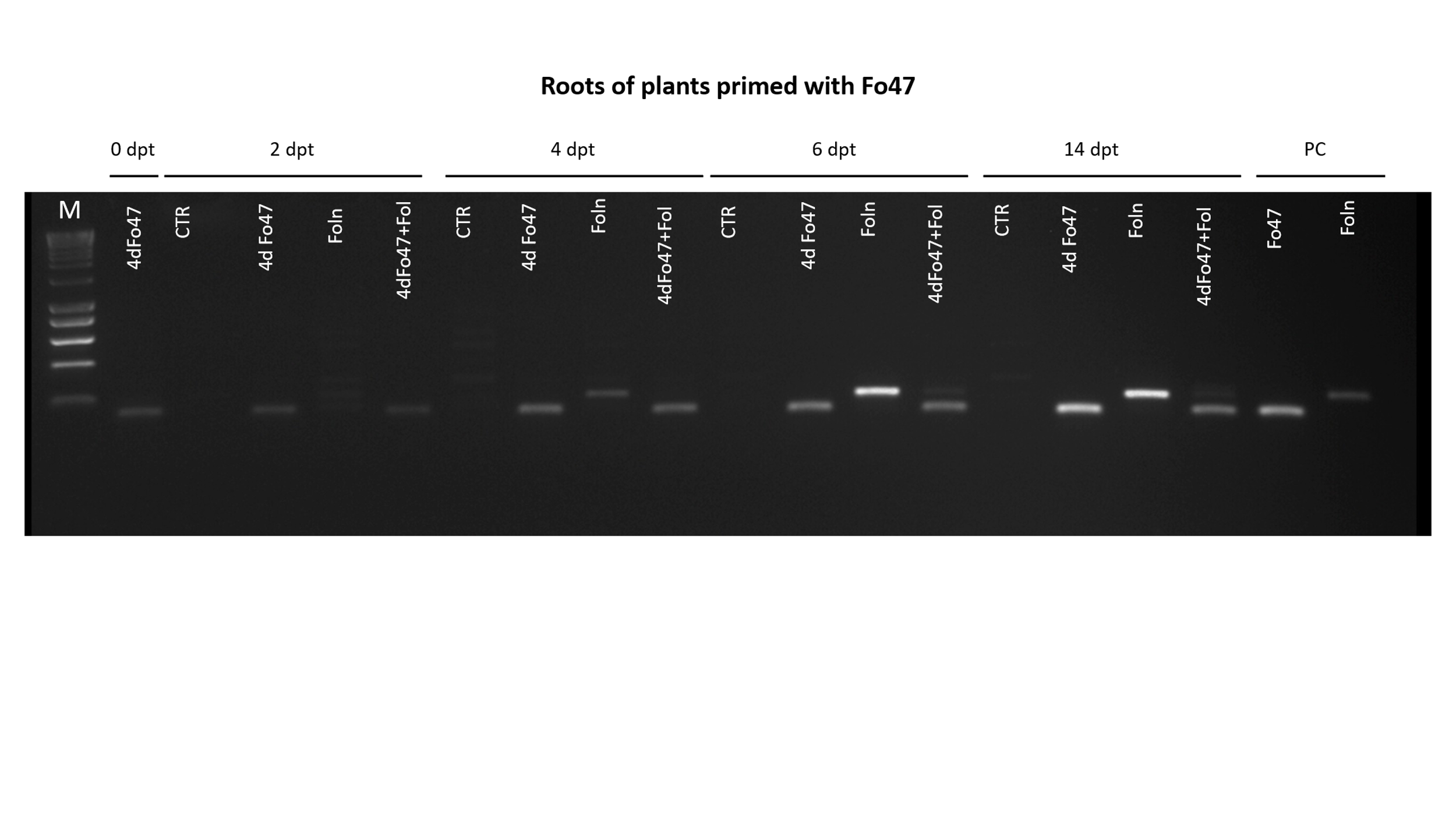

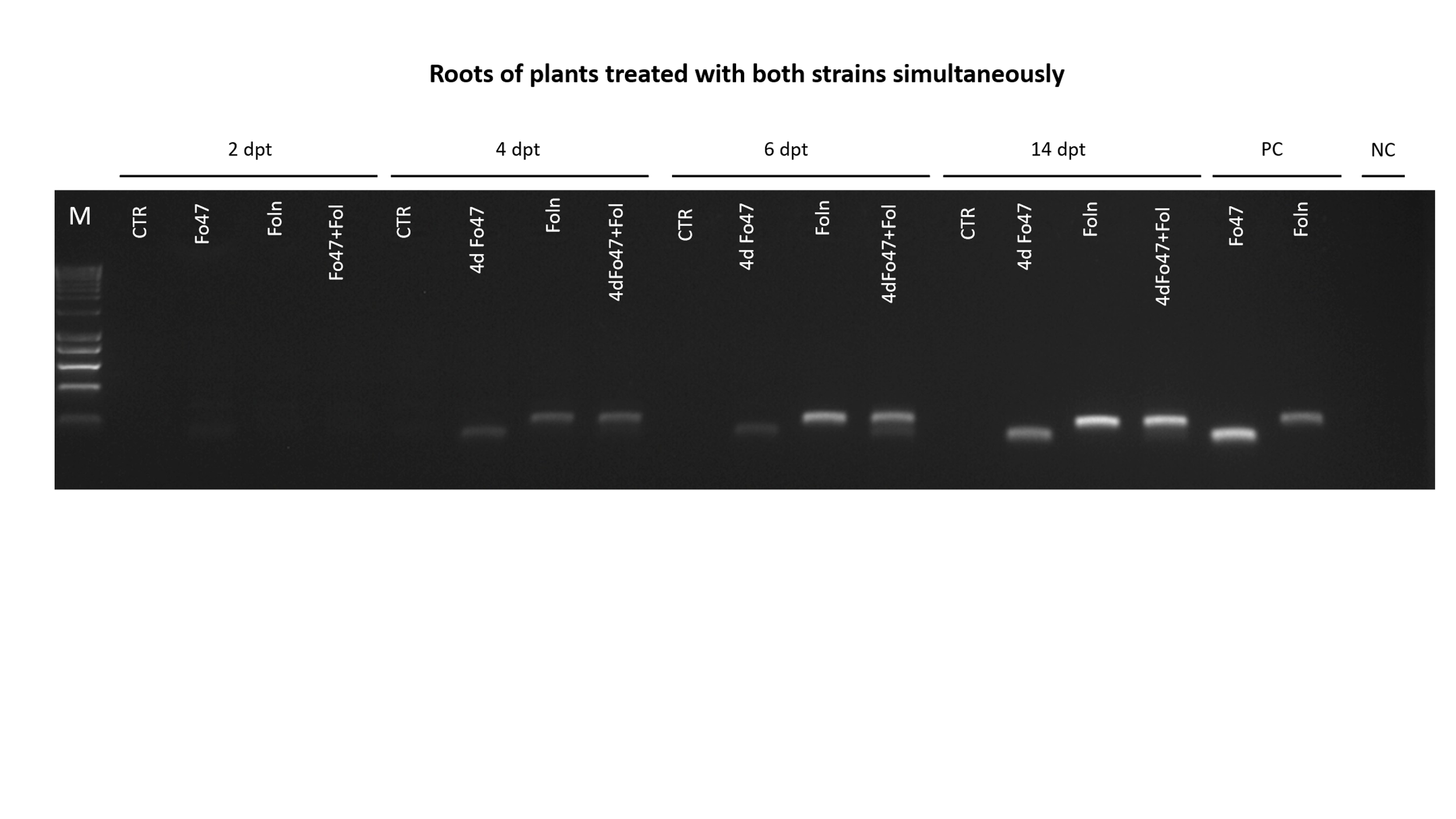


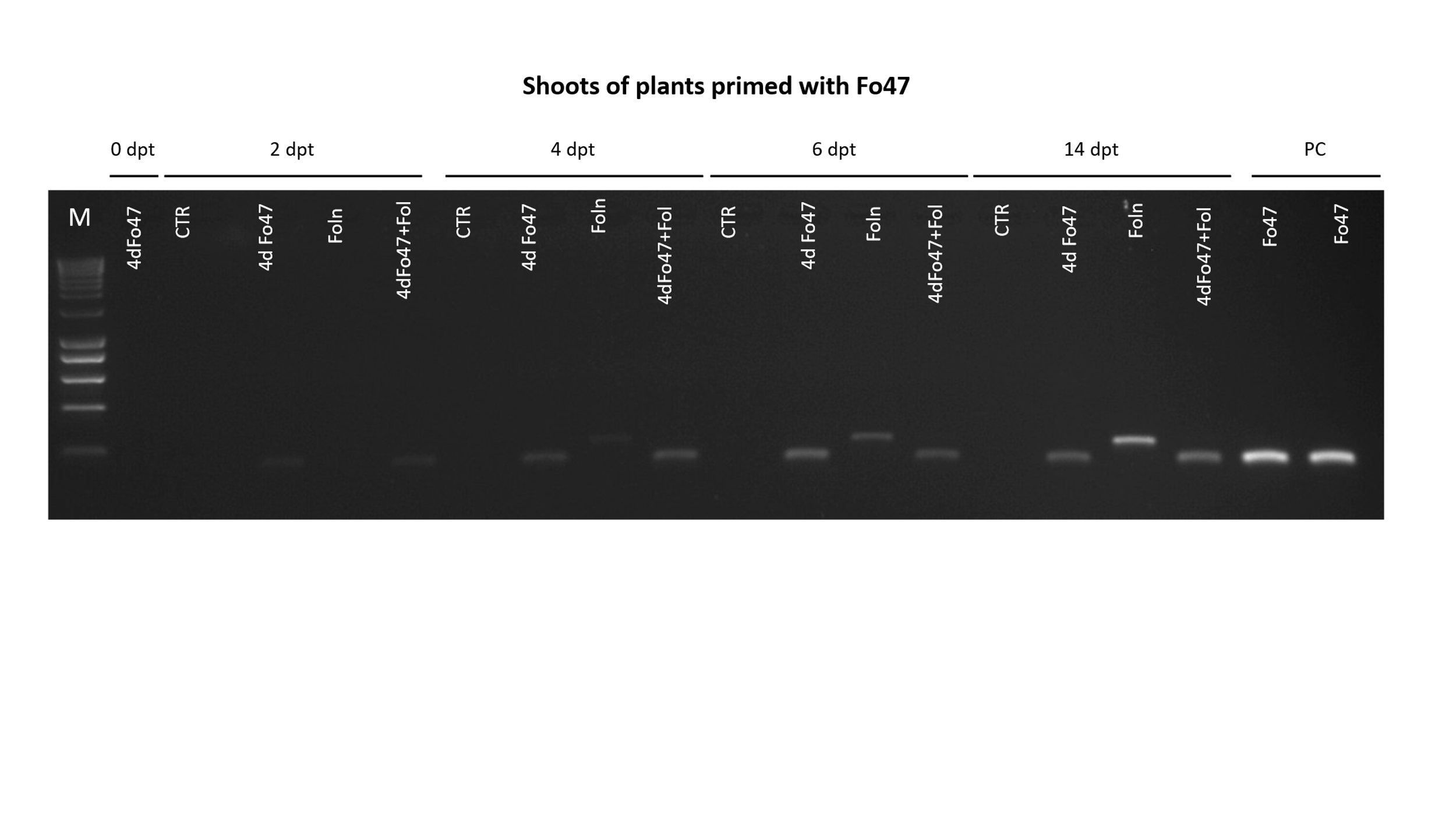

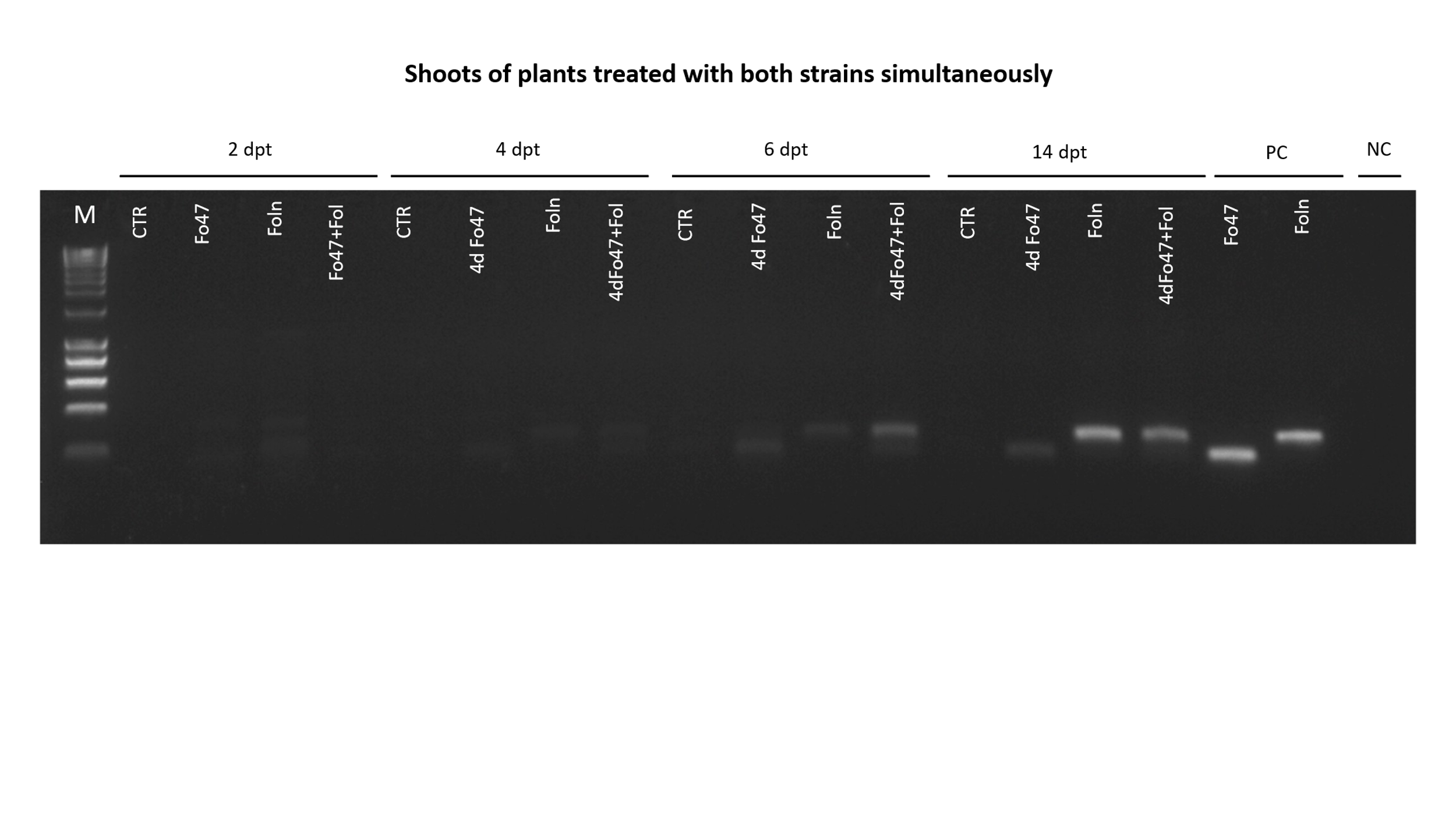


B)


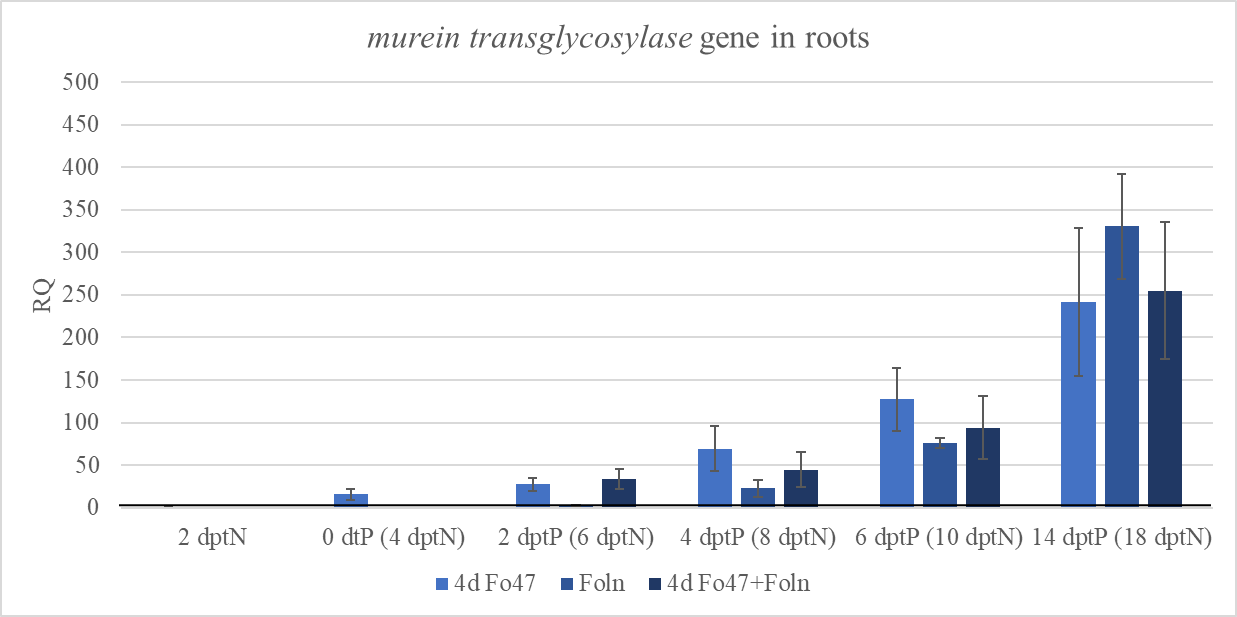

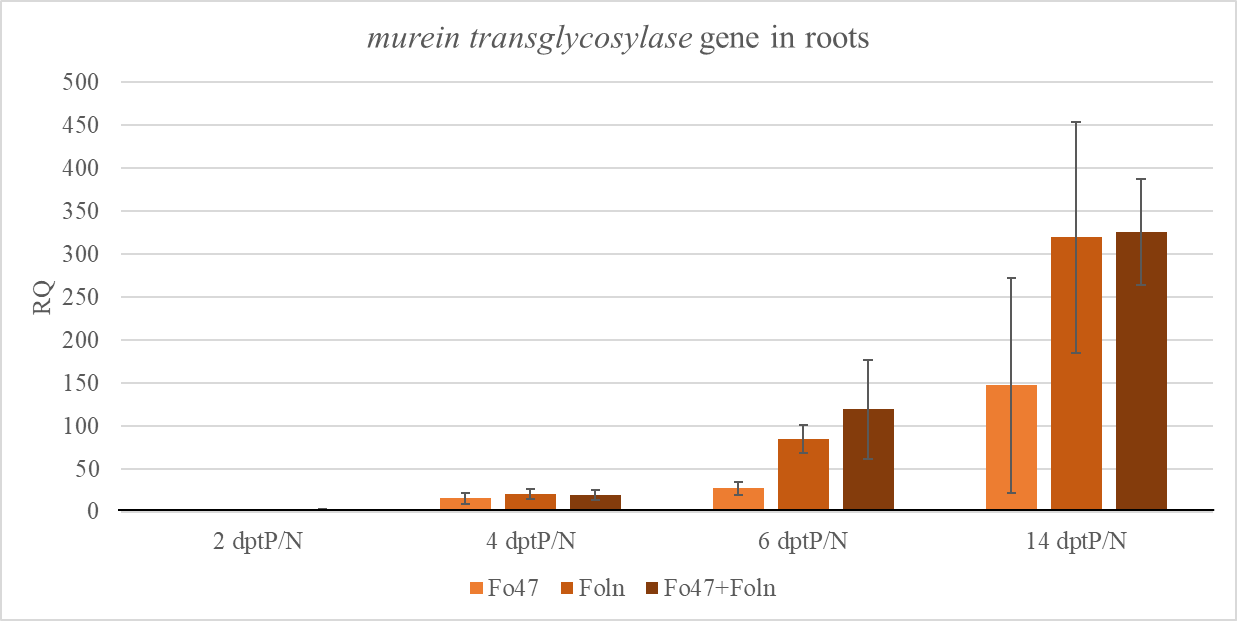


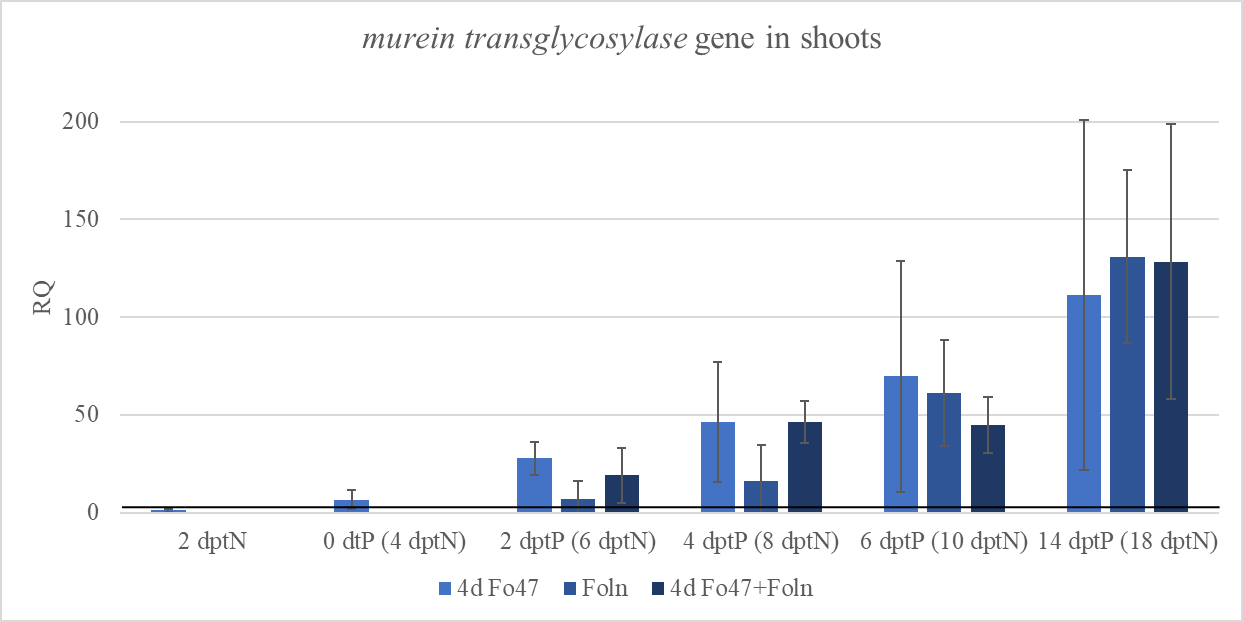

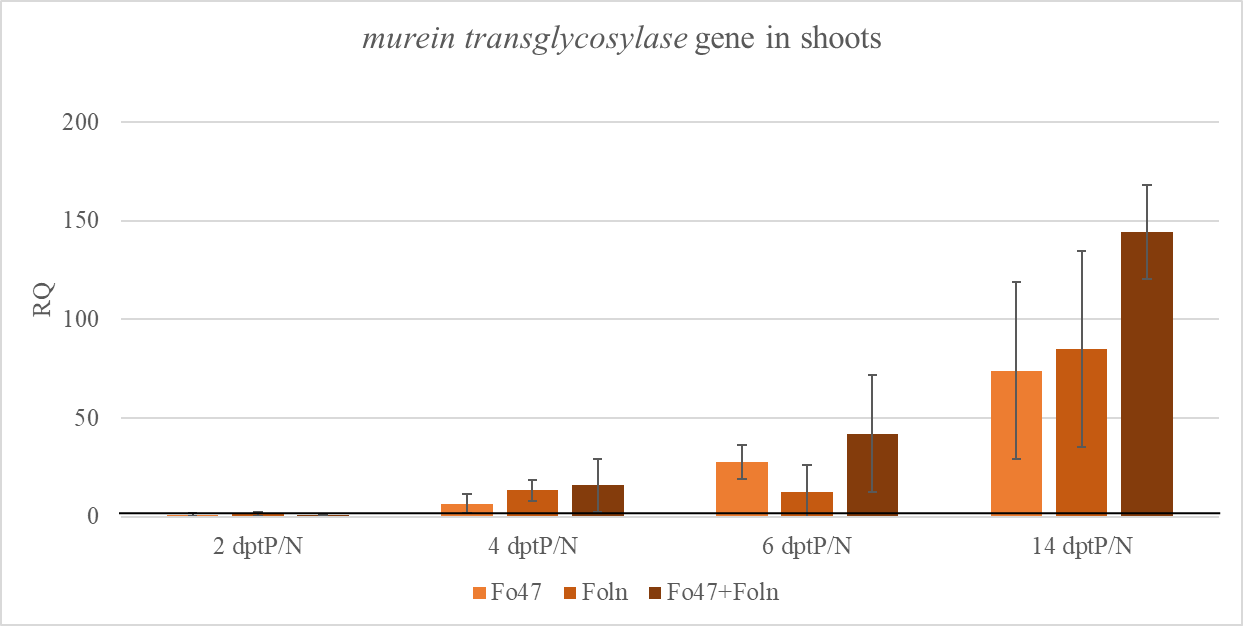


C)


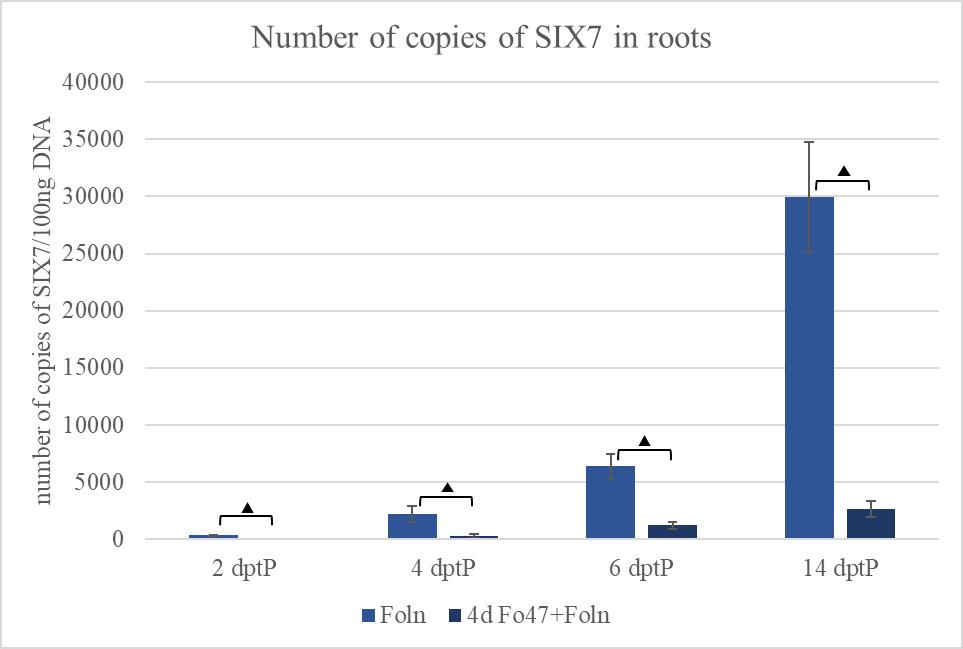

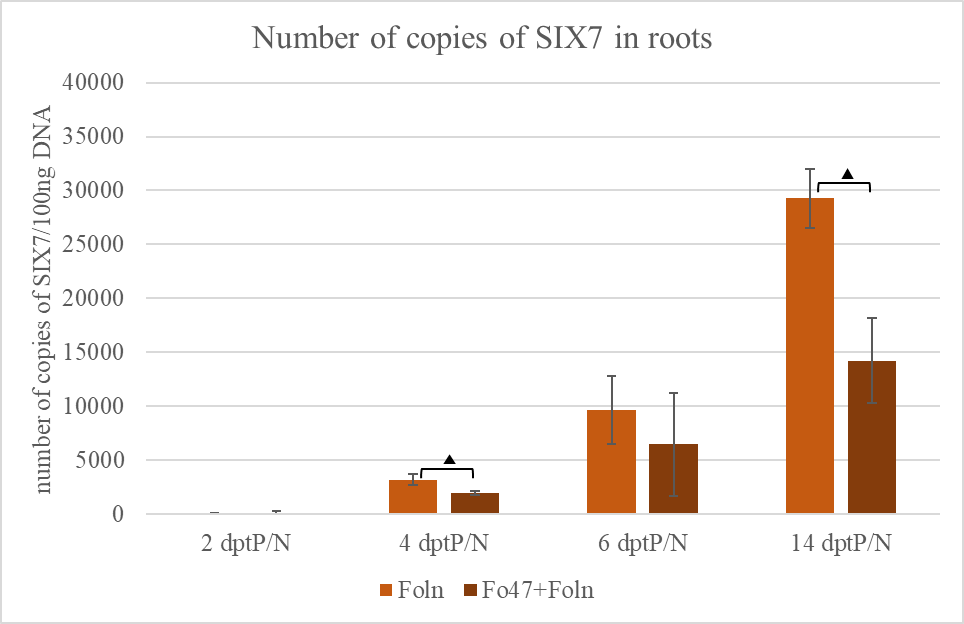


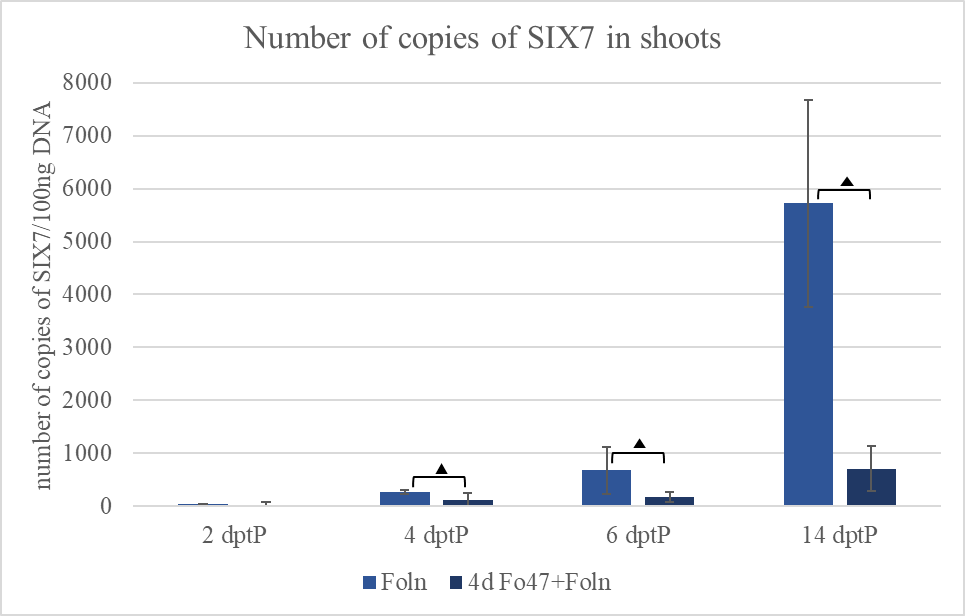

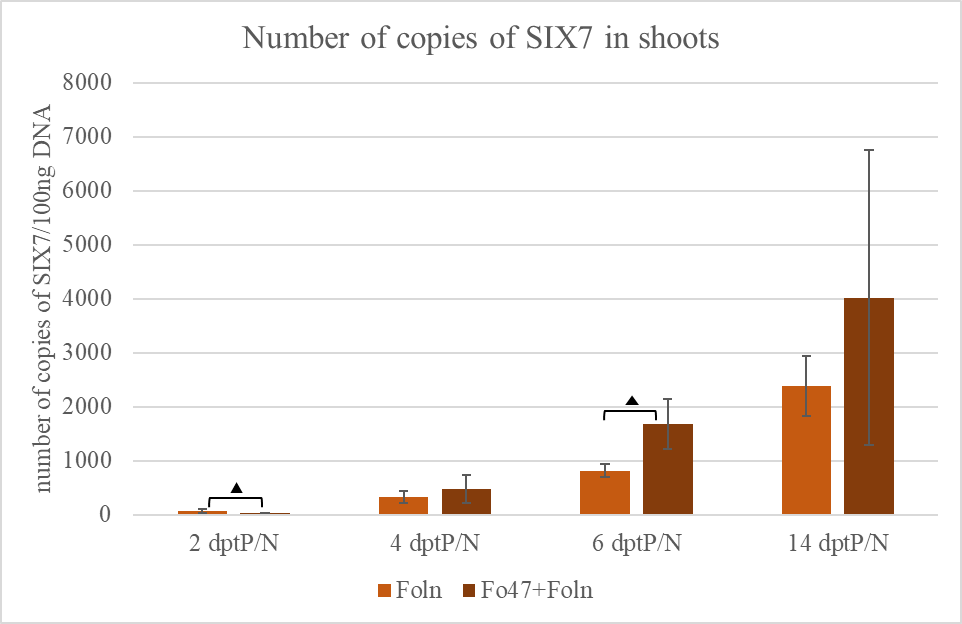

Supplement: Supplementary file 1 — Figure S1: (A) The fragment of the genome of Fo47 or Foln sequence obtained after the PCR reaction in roots and shoots of plants primed with non‐pathogenic strain Fo47 and plants treated with both strains simultaneously after 0, 2, 4, 6 and 14 days of Foln treatment. The amplified Fo47 genome fragment was 68 bp shorter than the Foln genome fragment. PC—positive control; NC—negative control. (B) Relative quantity of fungal murein transglycosylase gene in roots and shoots of plants primed with non‐pathogenic strain Fo47 and plants treated with both strains simultaneously. The data presented were obtained from real‐time PCR analysis. Flax actin served as the reference gene. The data represent the mean ± standard deviations from three independent experiments The significance of differences between groups was determined using a one‐way ANOVA, followed by Fisher's post hoc test. Differences were considered statistically significant when p < 0.05 (* for comparison to control, non‐treated plants from the same time point as the sample; ▲for comparison of plants primed with non‐pathogenic strain Fo47 or plants treated with both strains simultaneously with Foln treated plants from the same time point as sample). (C) Number of copies of the six7 gene of pathogenic Fusarium oxysporum strain in the root and shoot of flax plants primed with the non‐pathogenic strain Fo47 and plants treated with both strains of the fungus simultaneously after 2, 4, 6 and 14 days of Foln treatment. The data represent the mean ± standard deviations from three independent experiments The significance of differences between groups was determined using a one‐way ANOVA, followed by Fisher's post hoc test. Differences were considered statistically significant when p < 0.05 (* for comparison to control, non‐treated plants from the same time point as the sample; ▲for comparison of plants primed with non‐pathogenic strain Fo47 or plants treated with both strains simultaneously with Foln treated plants from t [file EMI4-18-e70263-s003.docx]

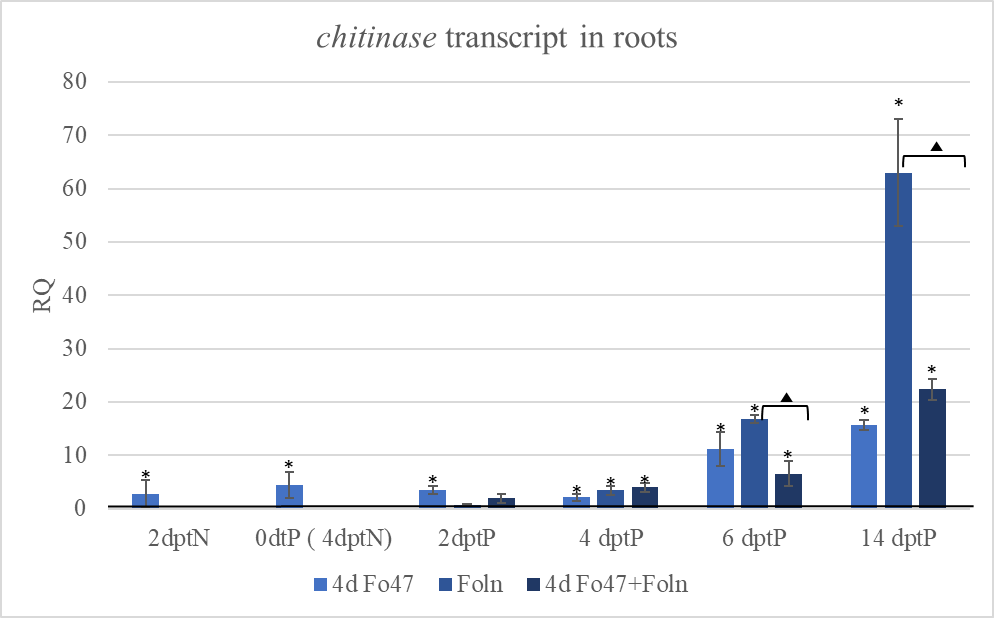

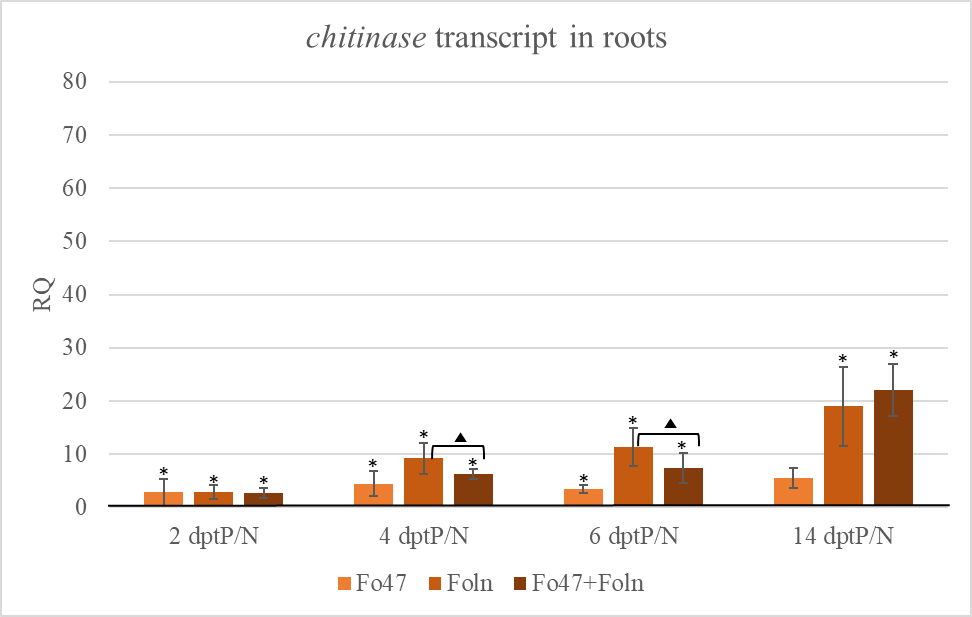

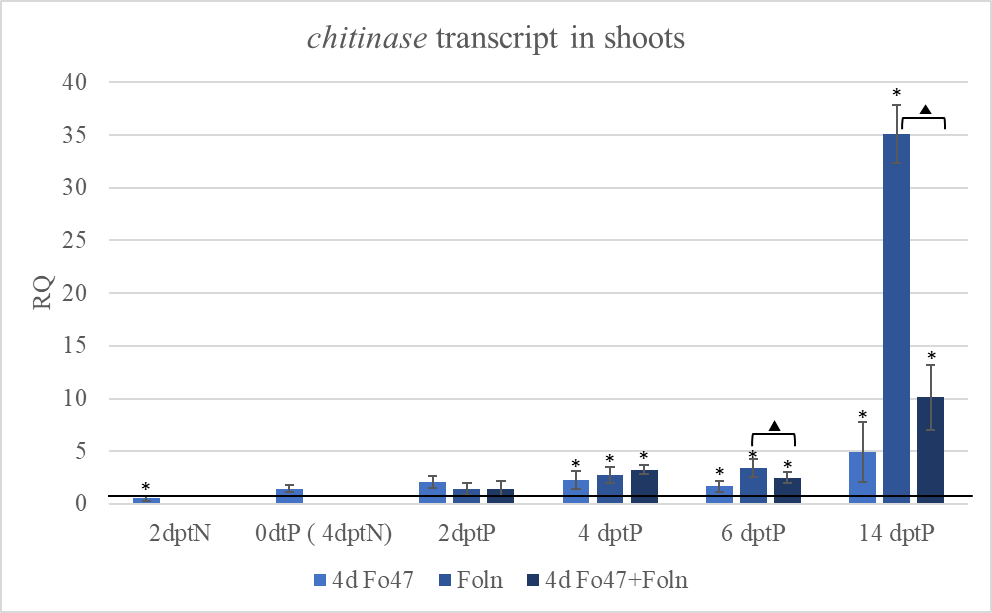

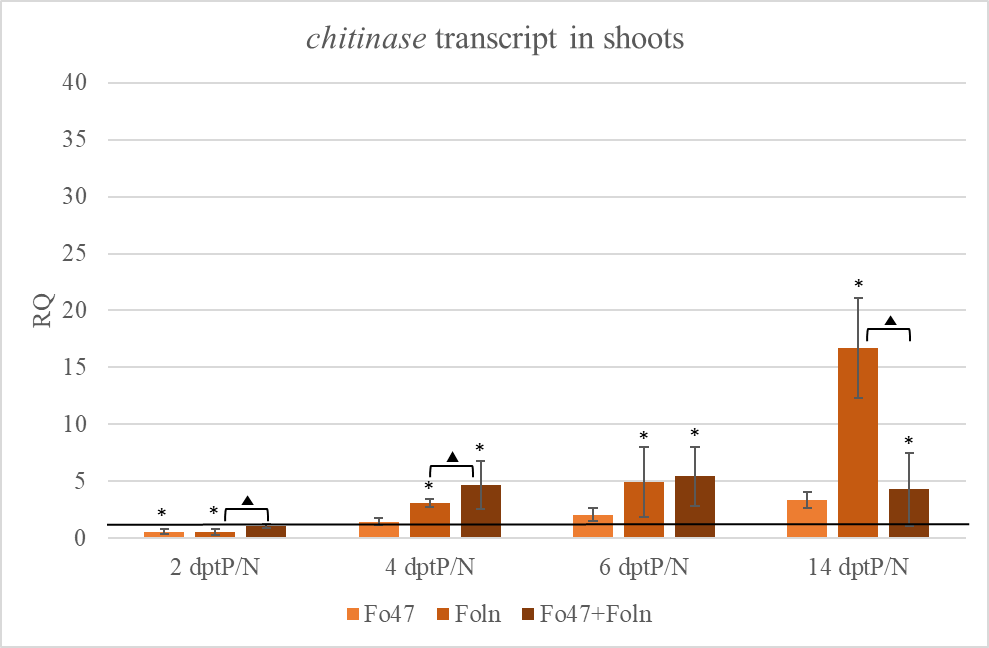


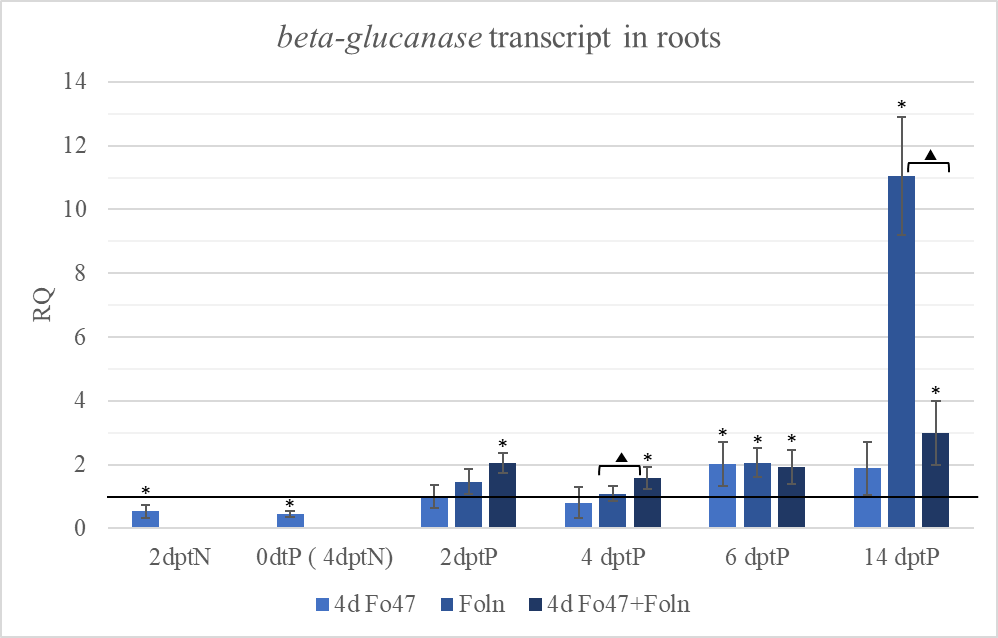

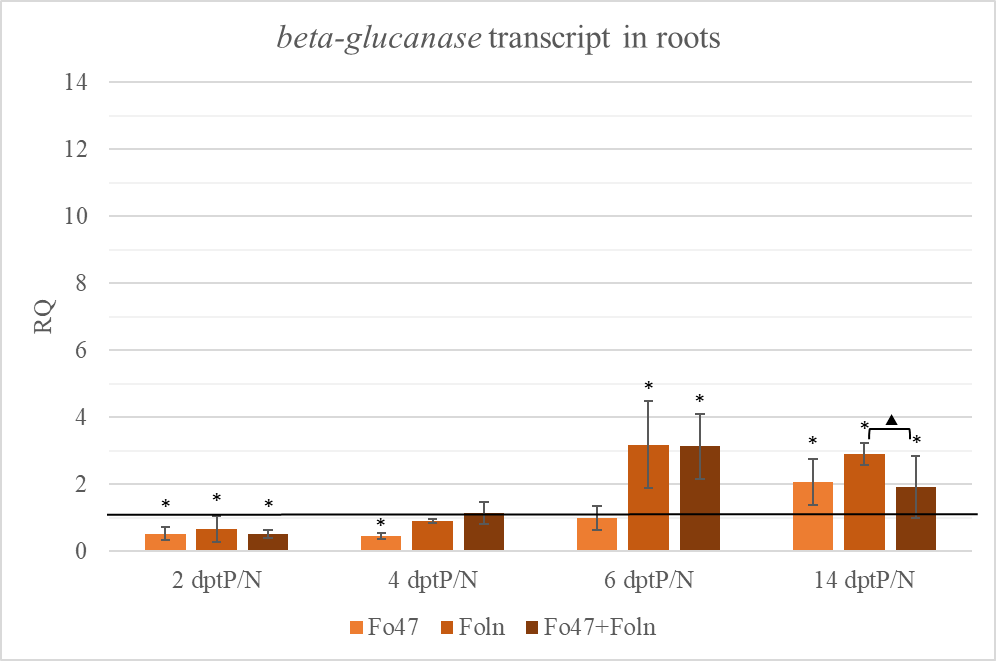

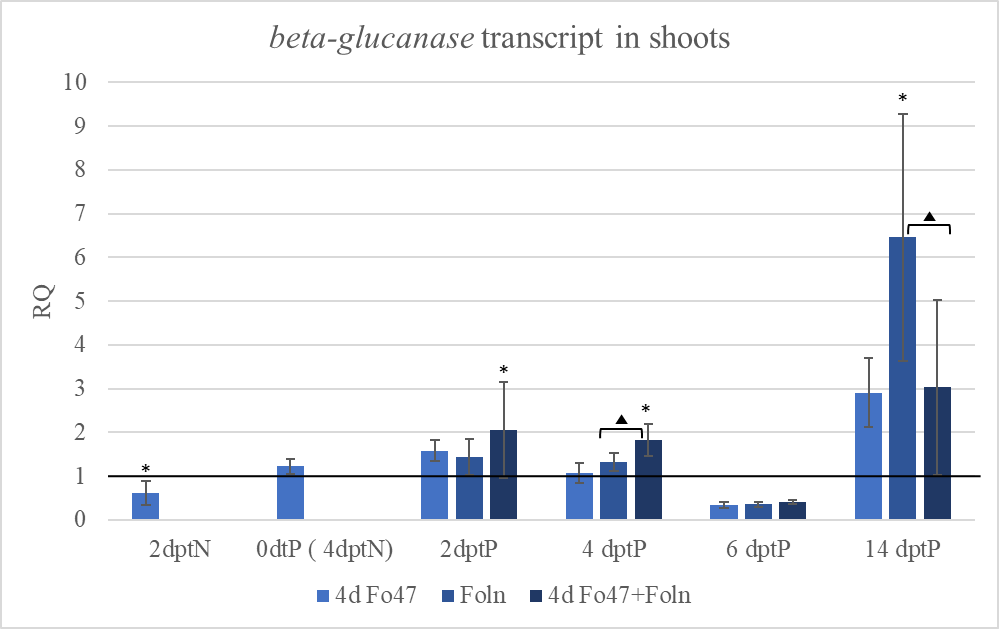

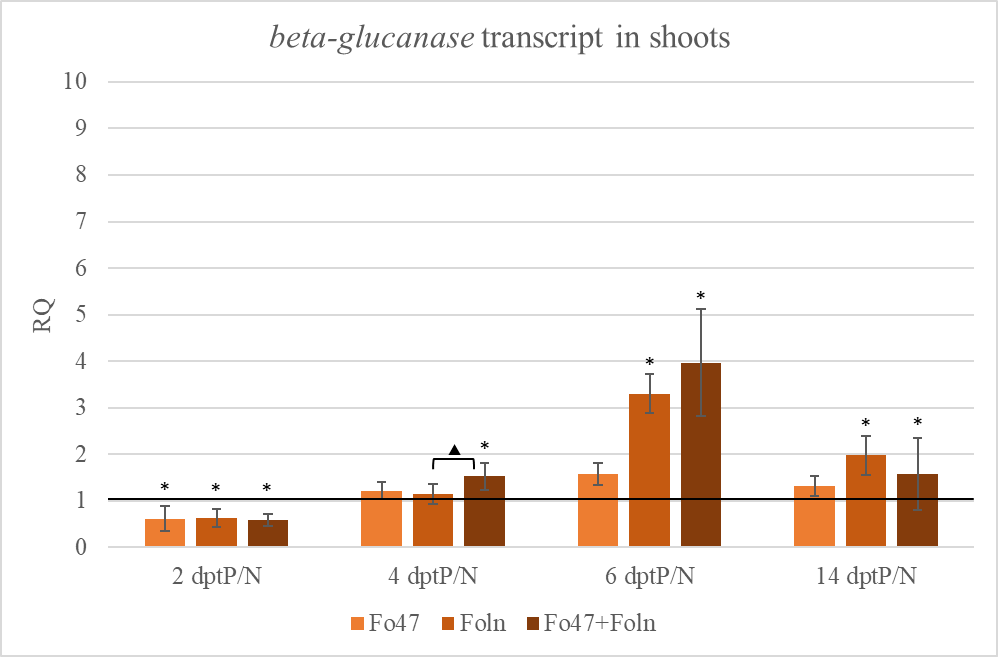


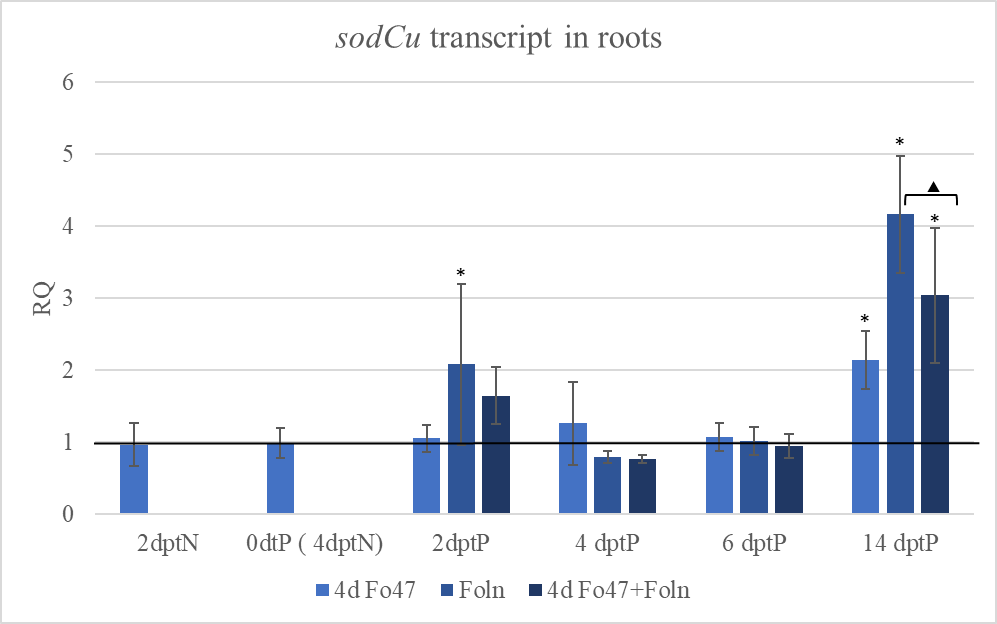

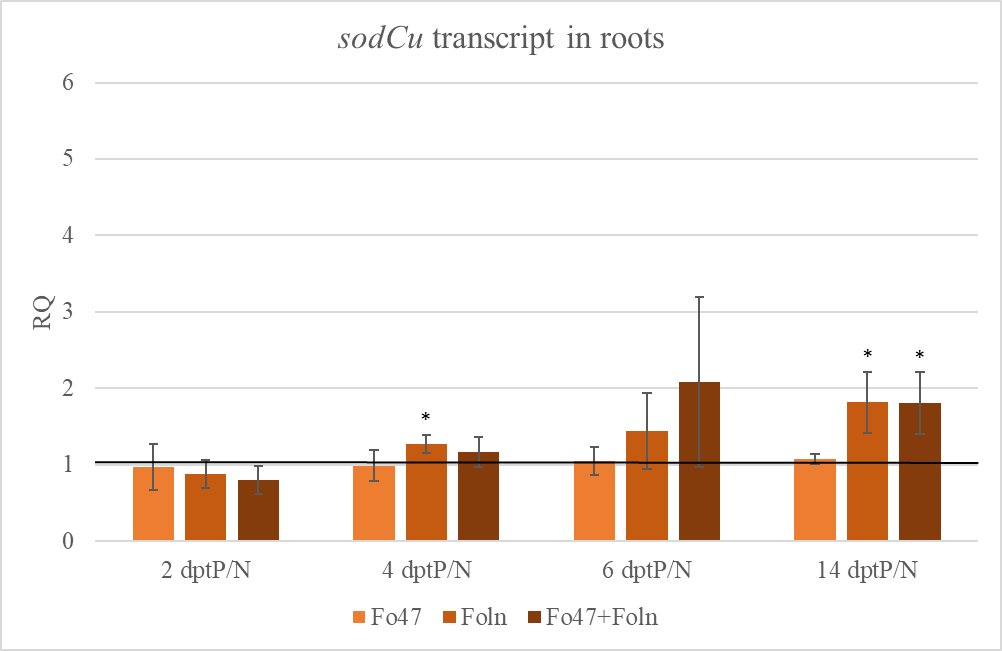

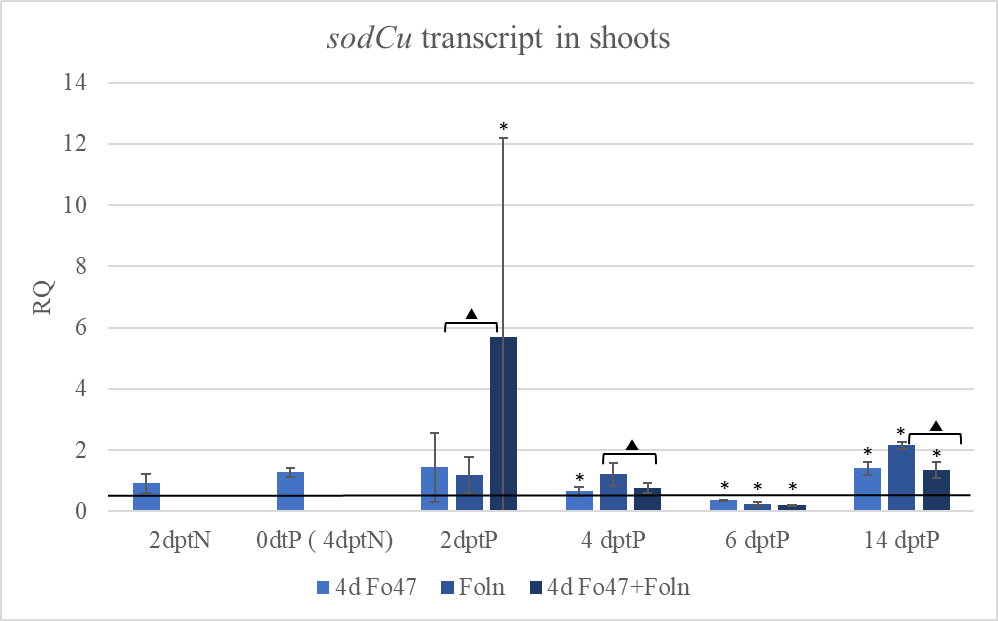

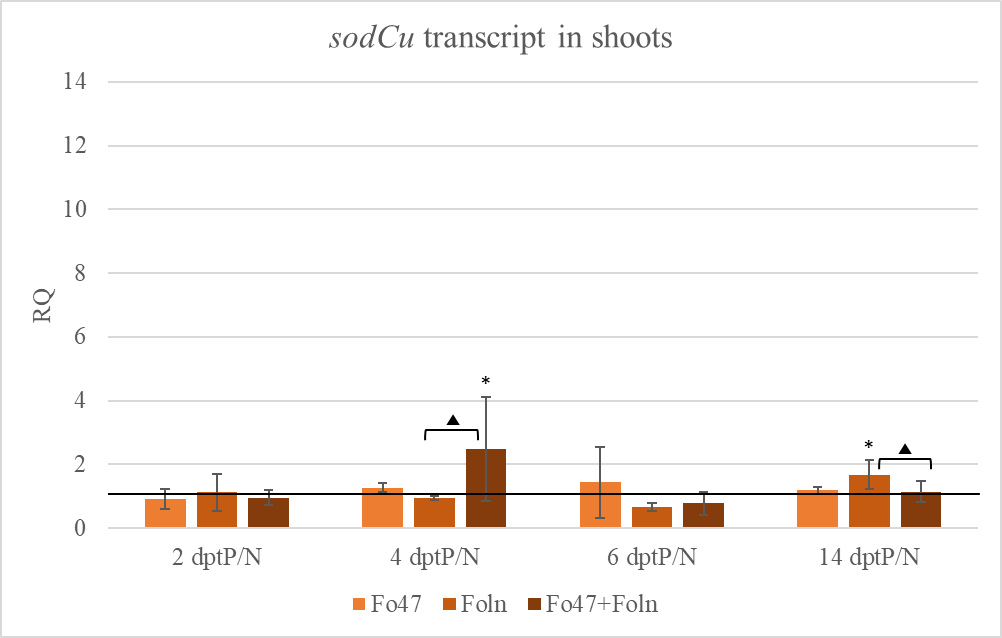


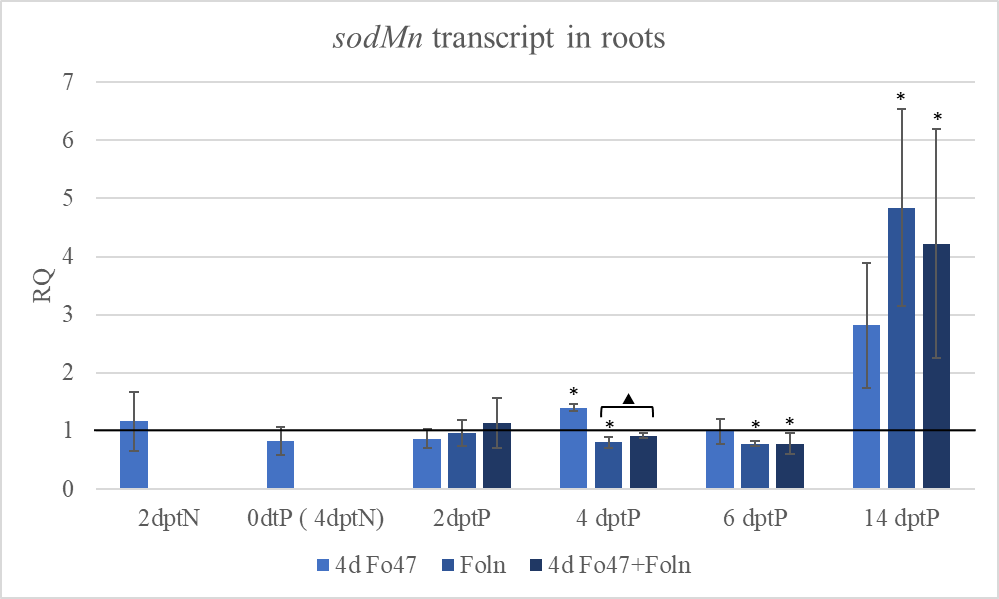

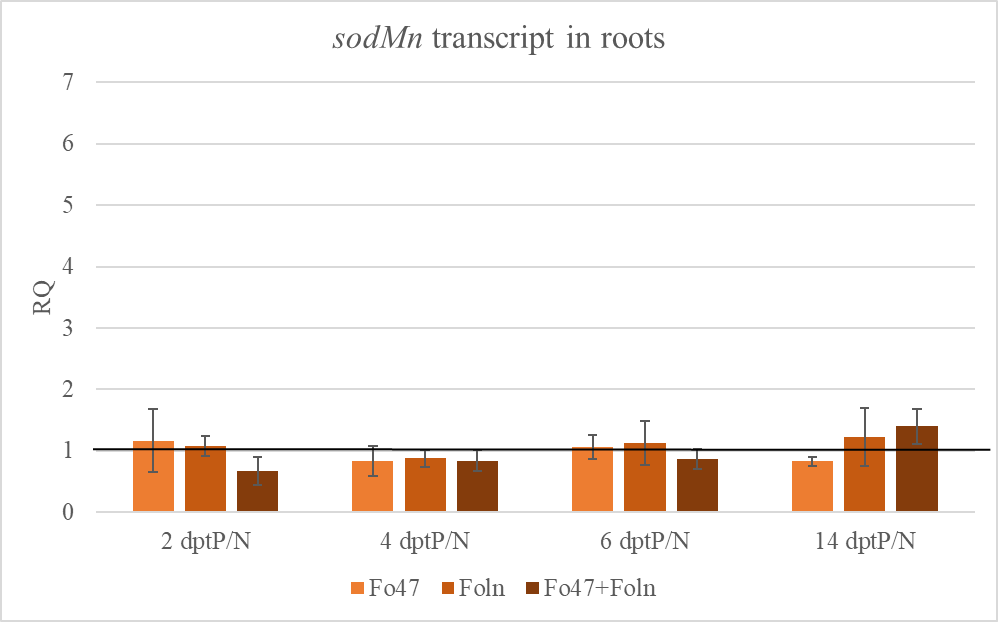

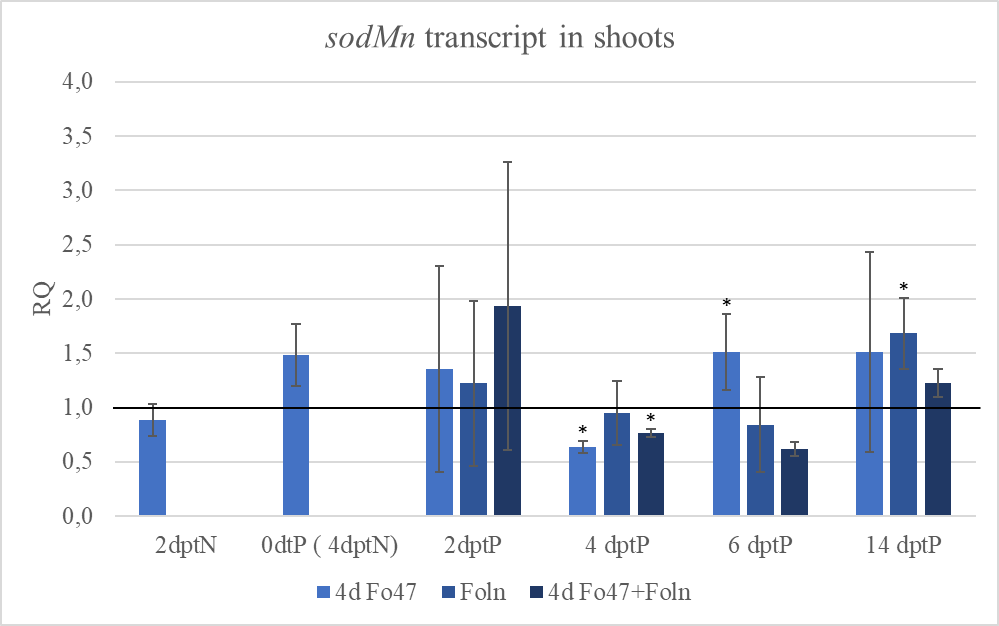

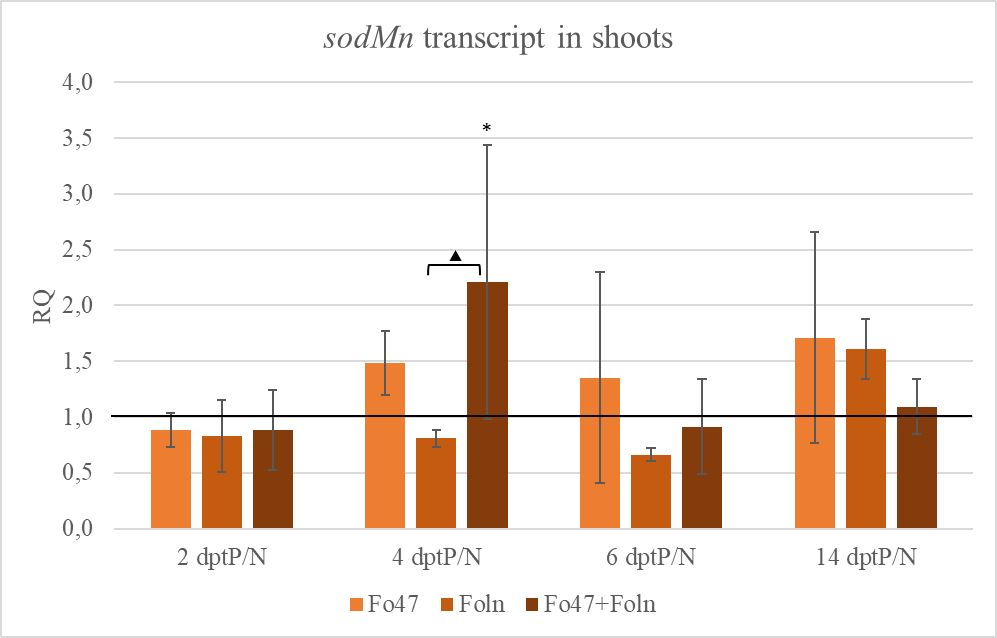


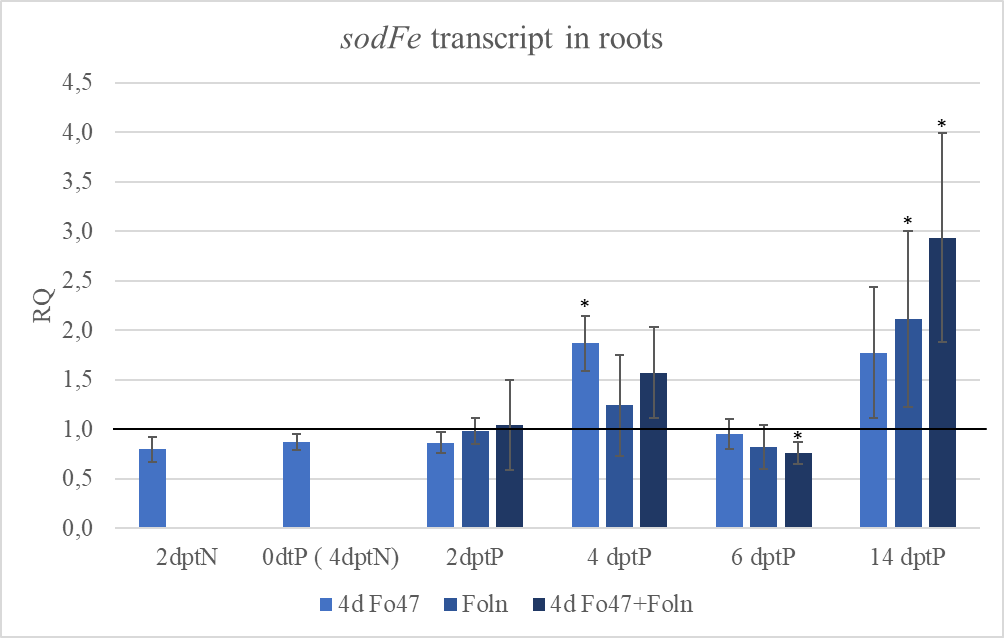

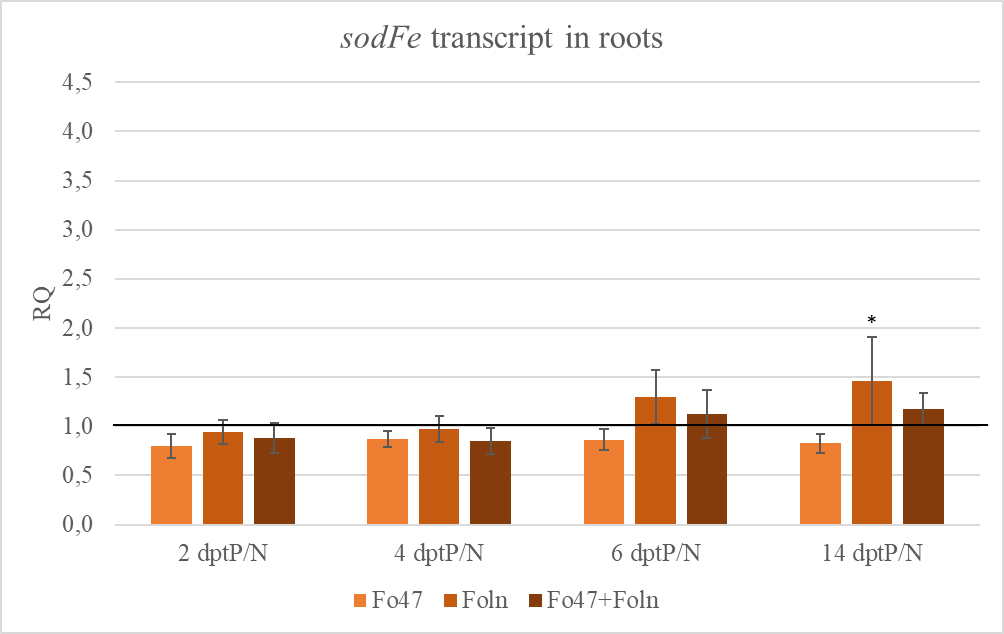

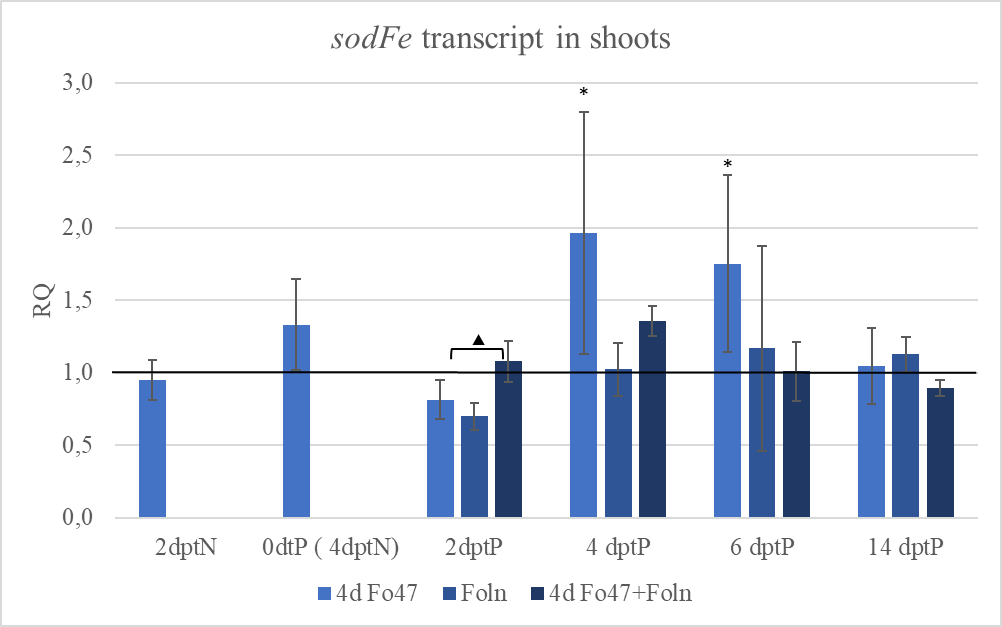

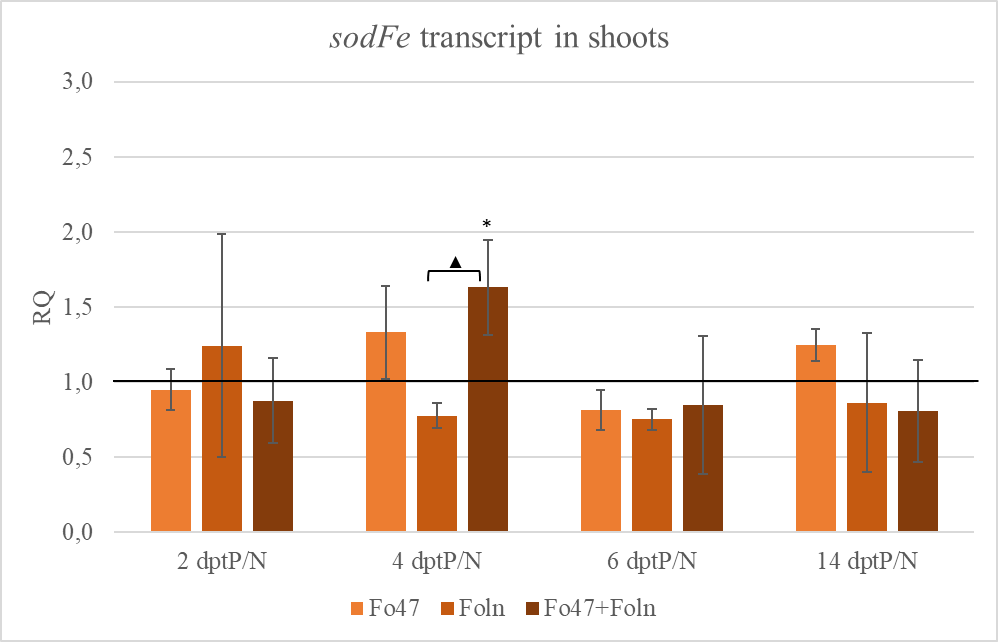


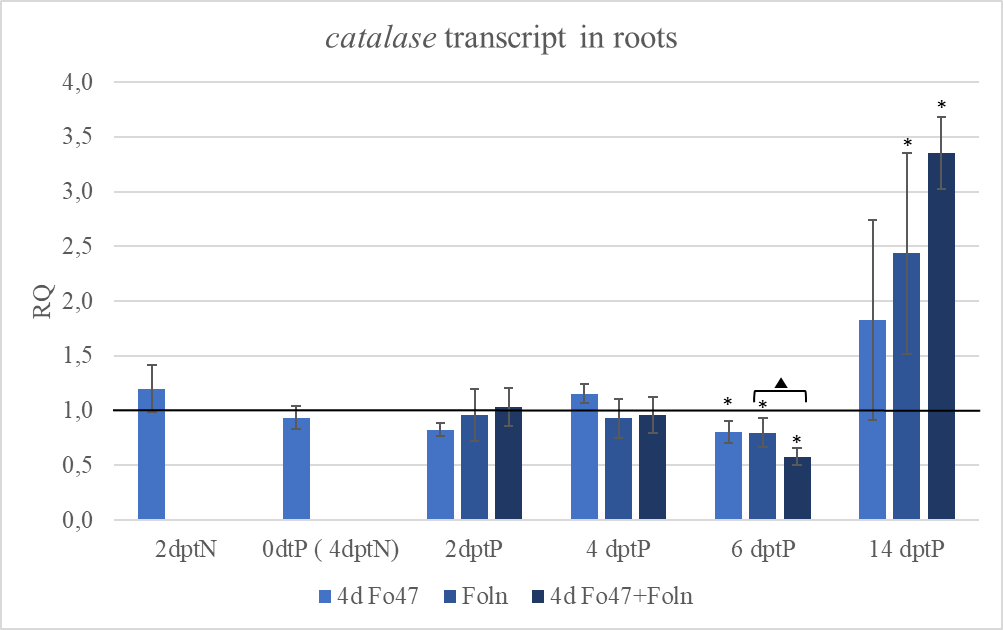

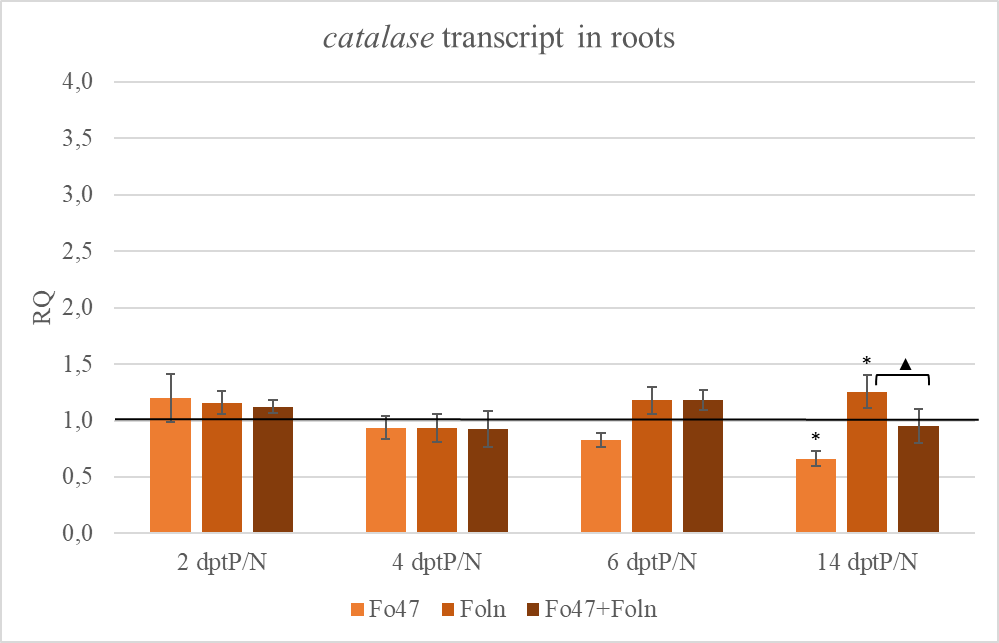

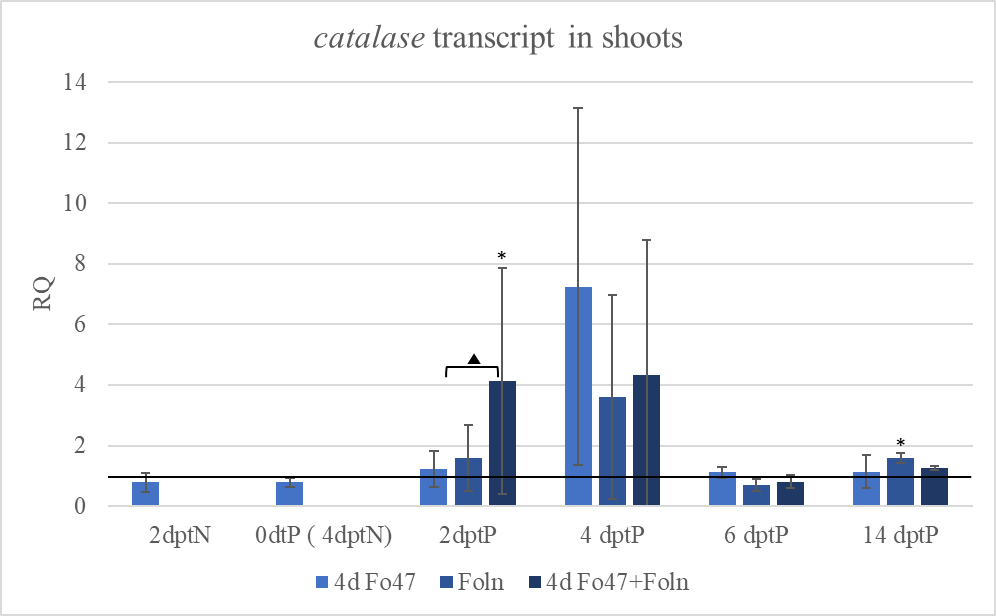

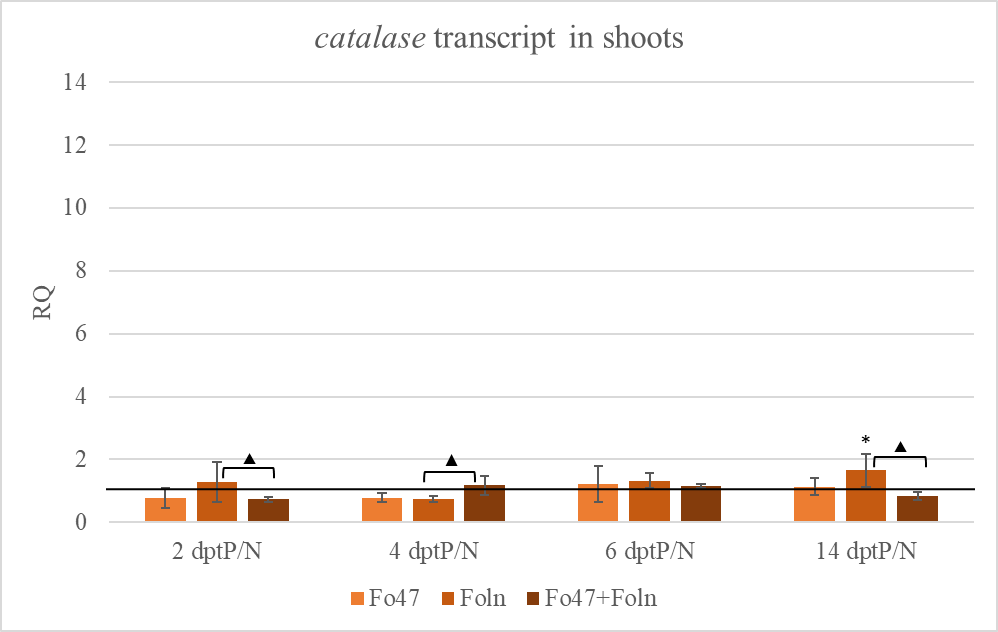


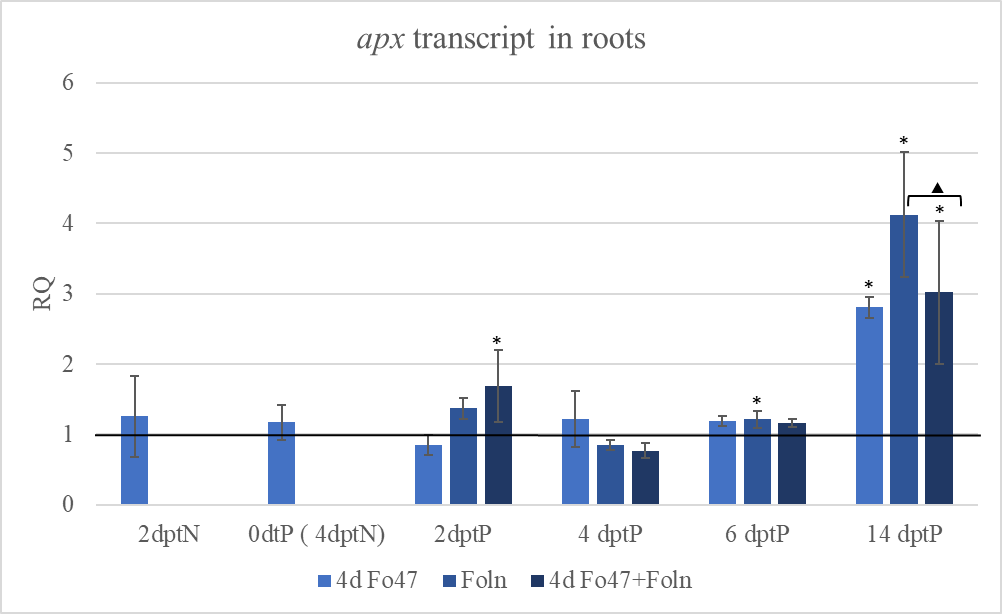

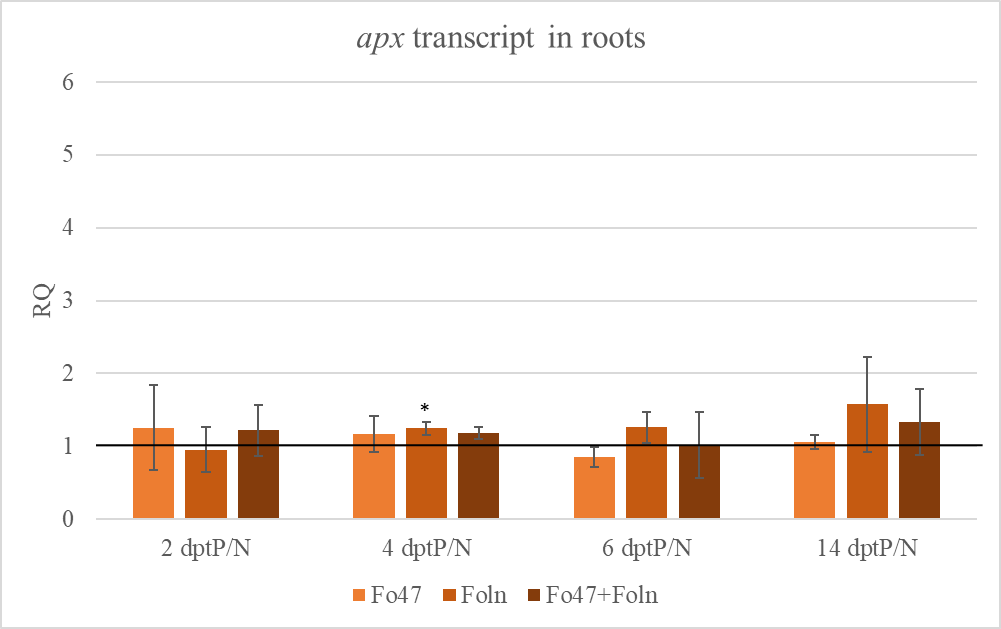

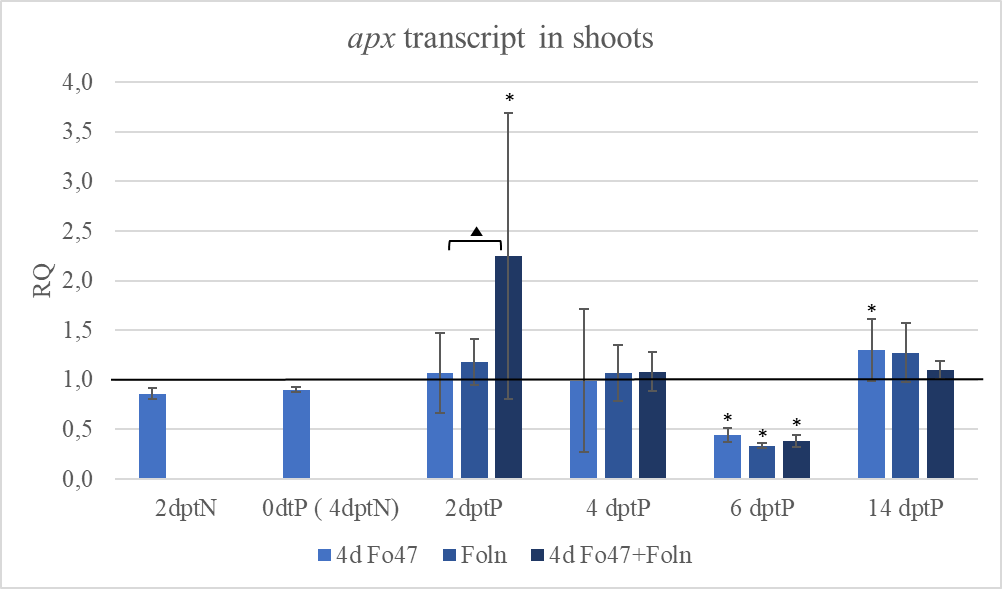

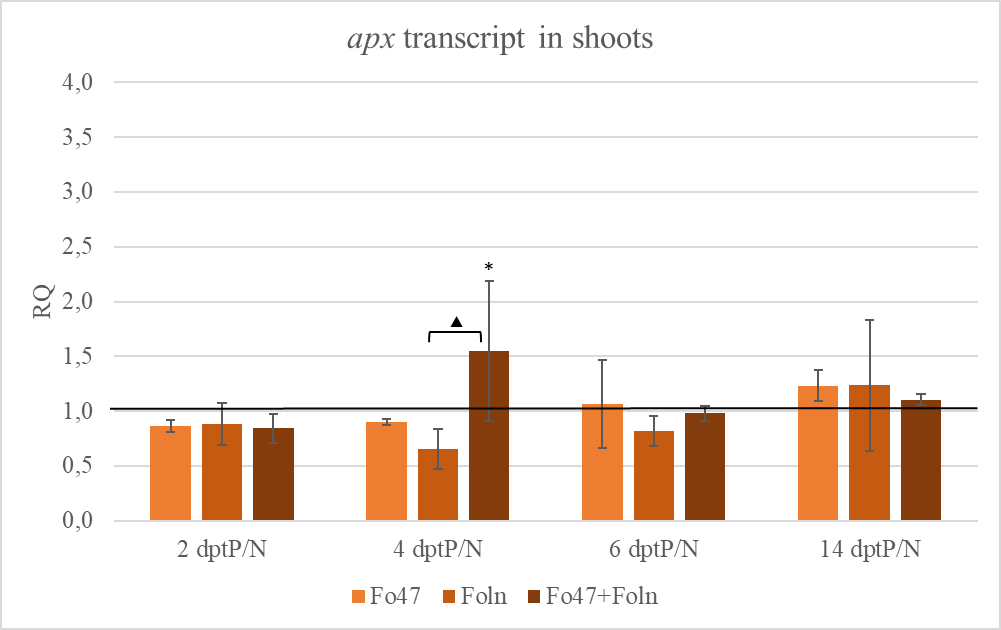


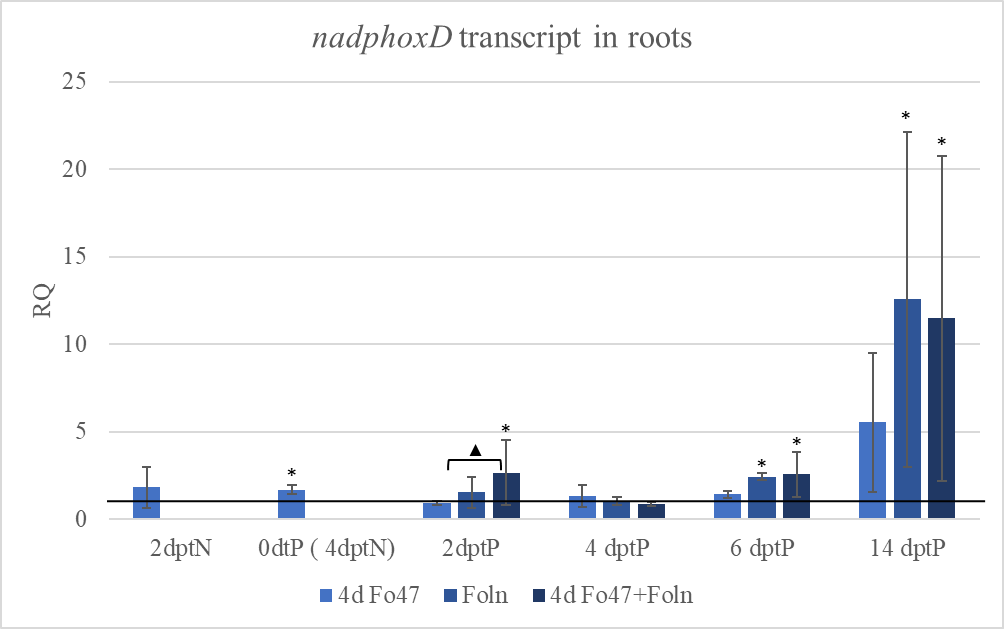

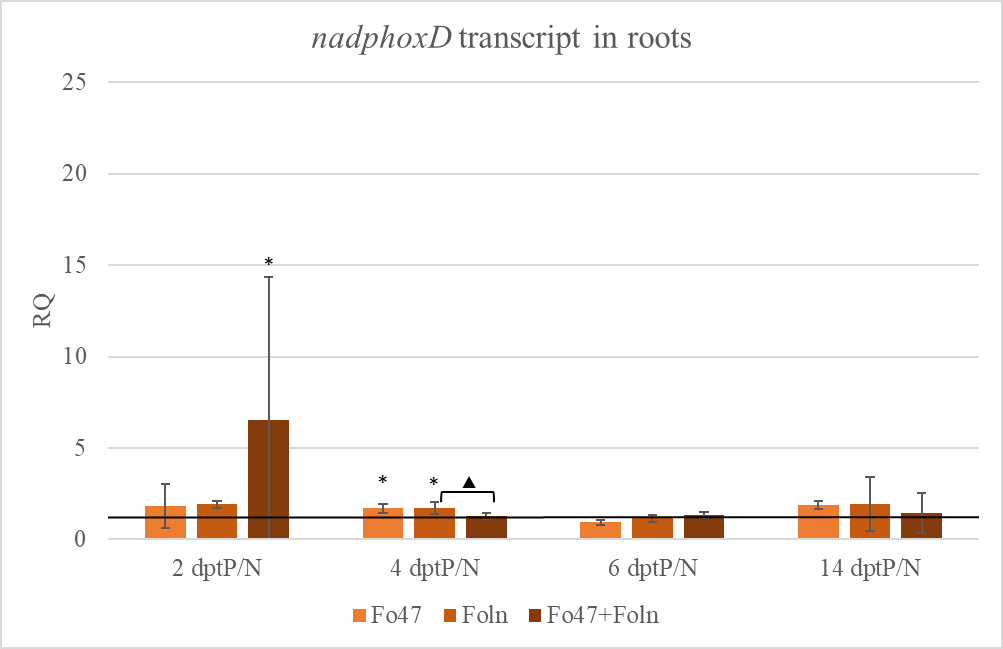

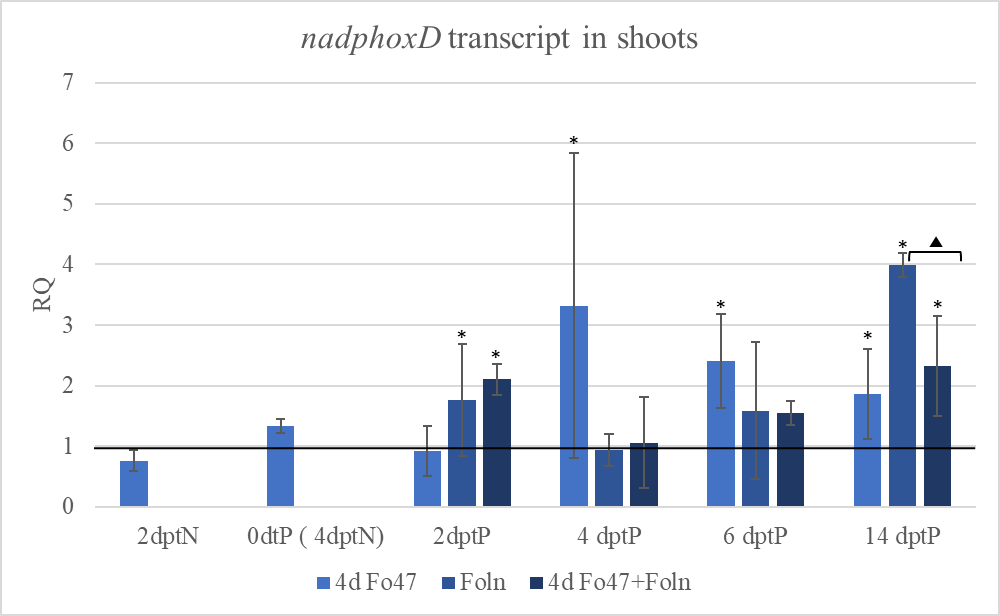

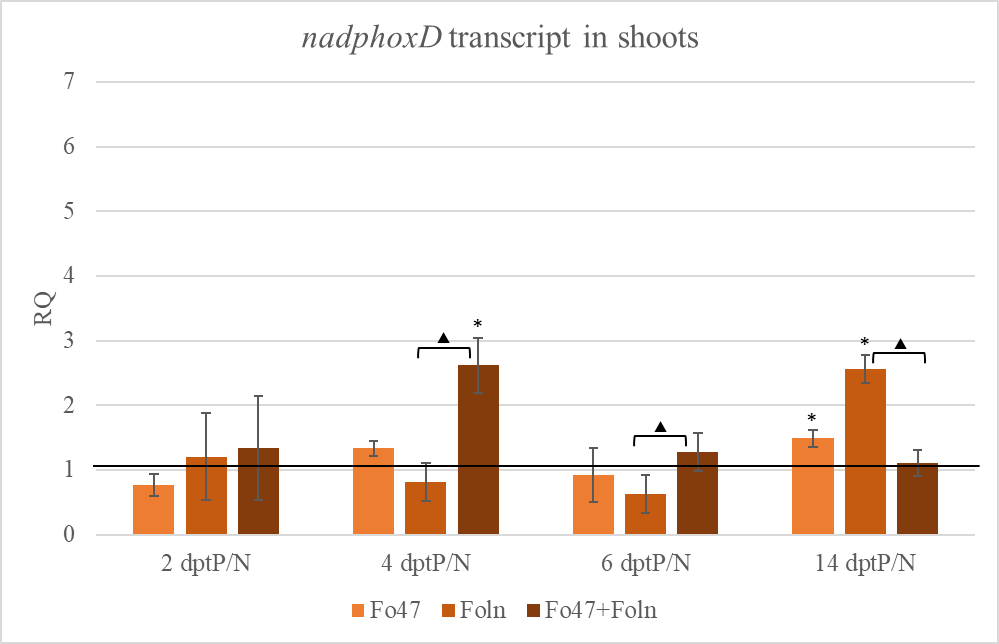


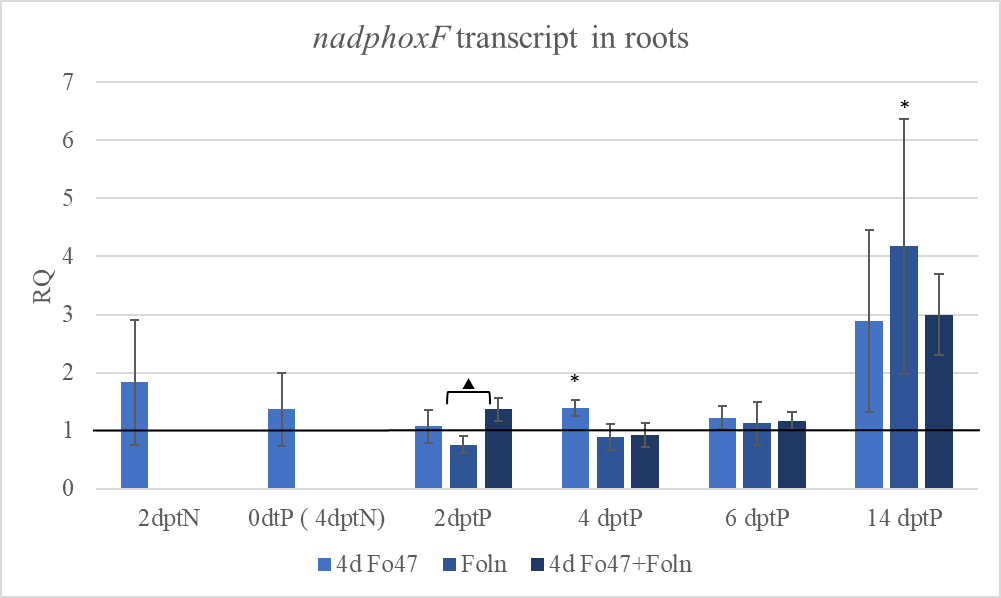

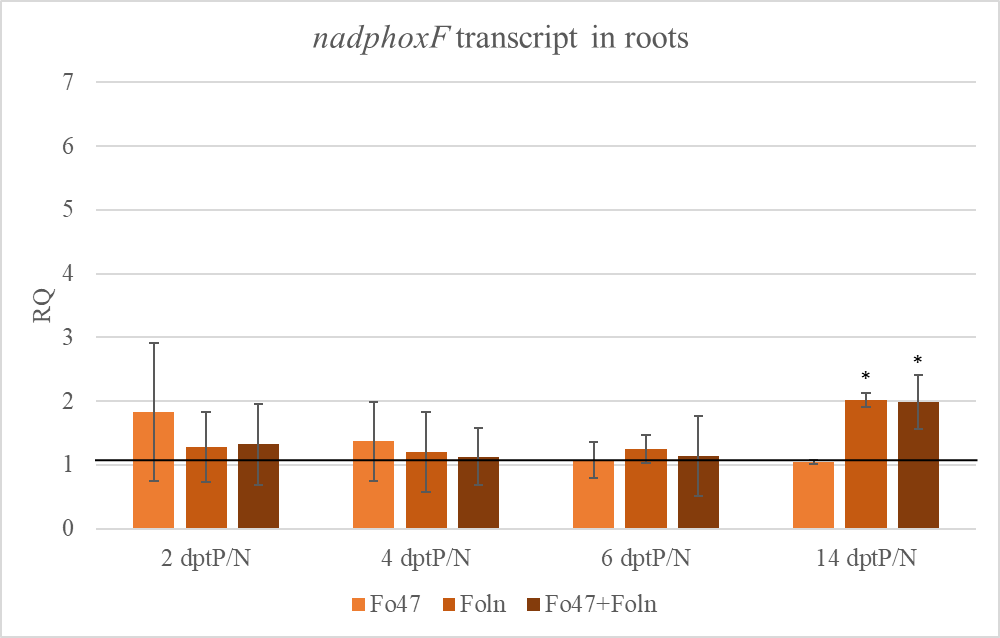

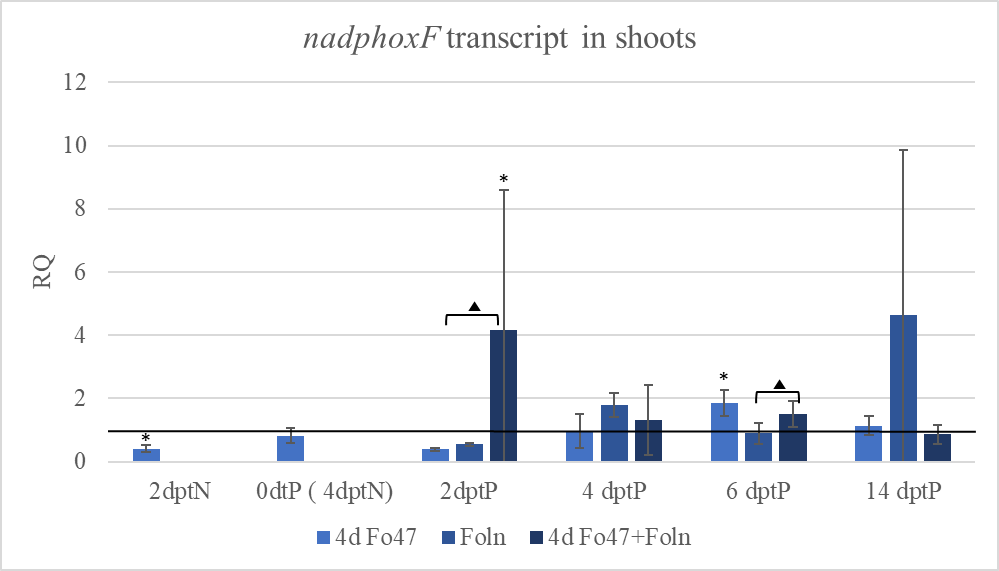

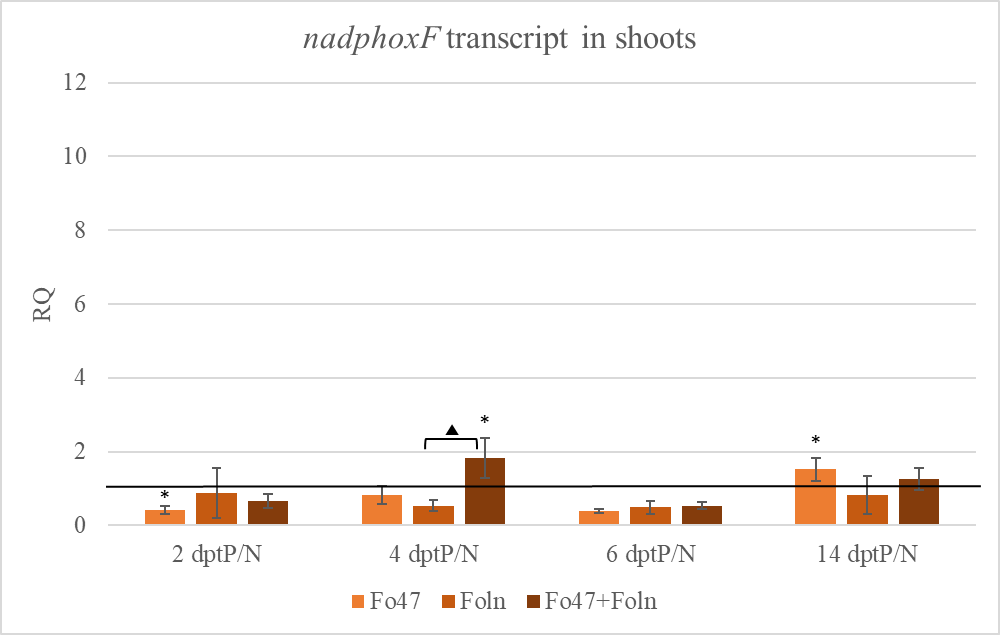


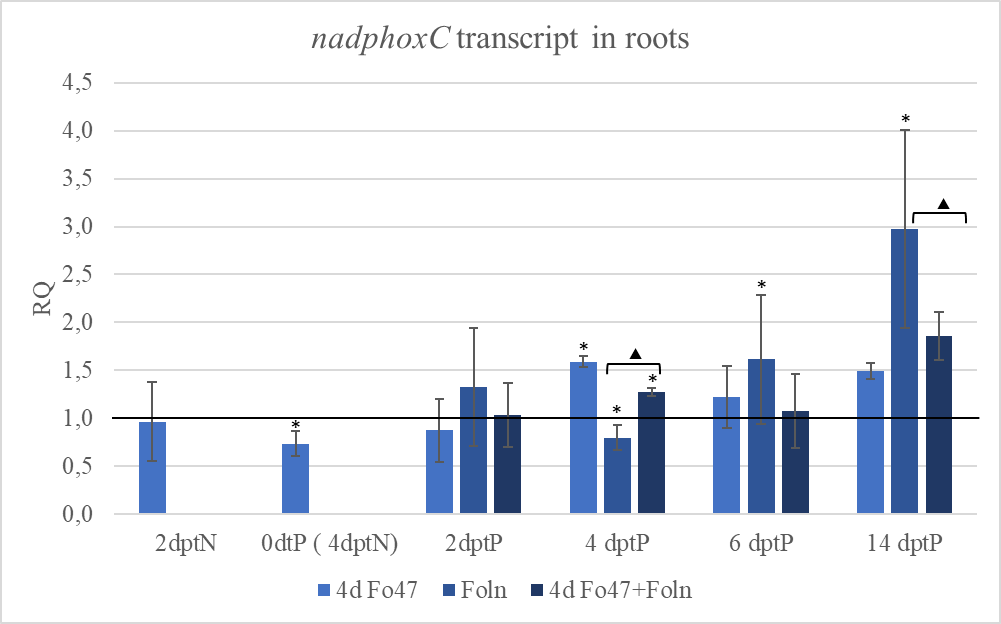

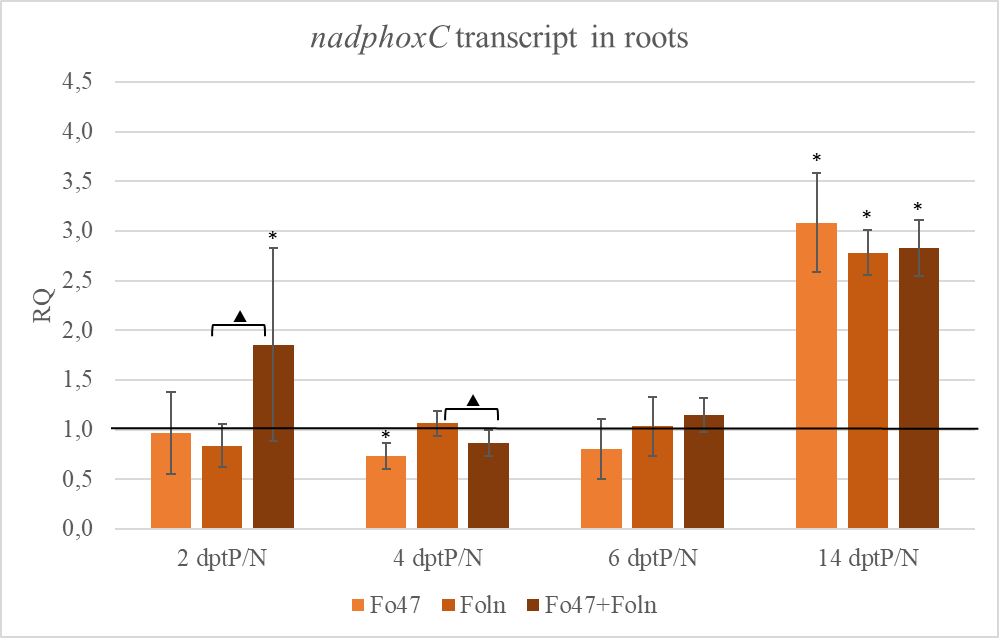

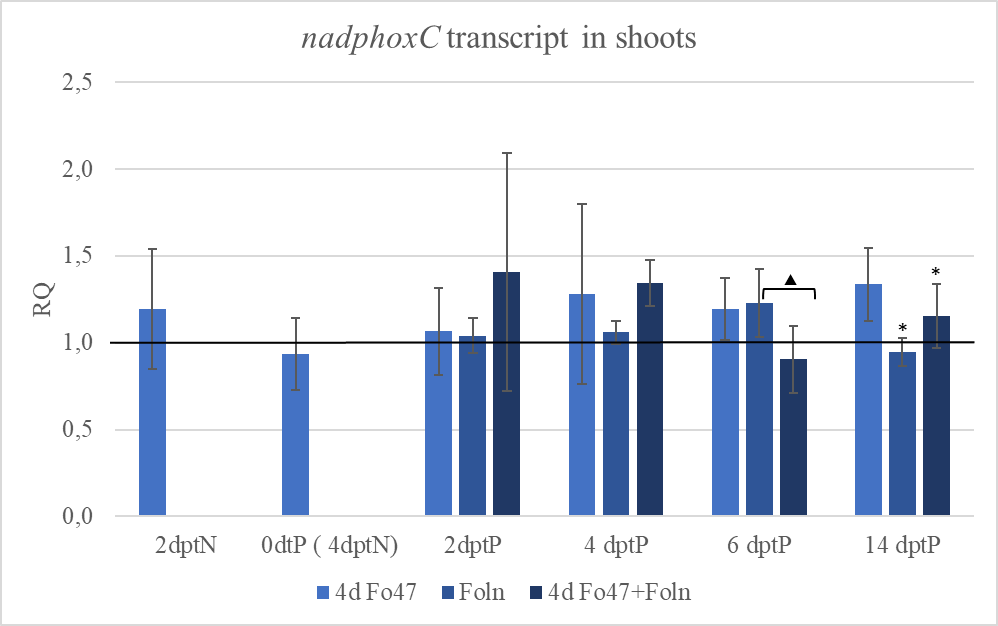

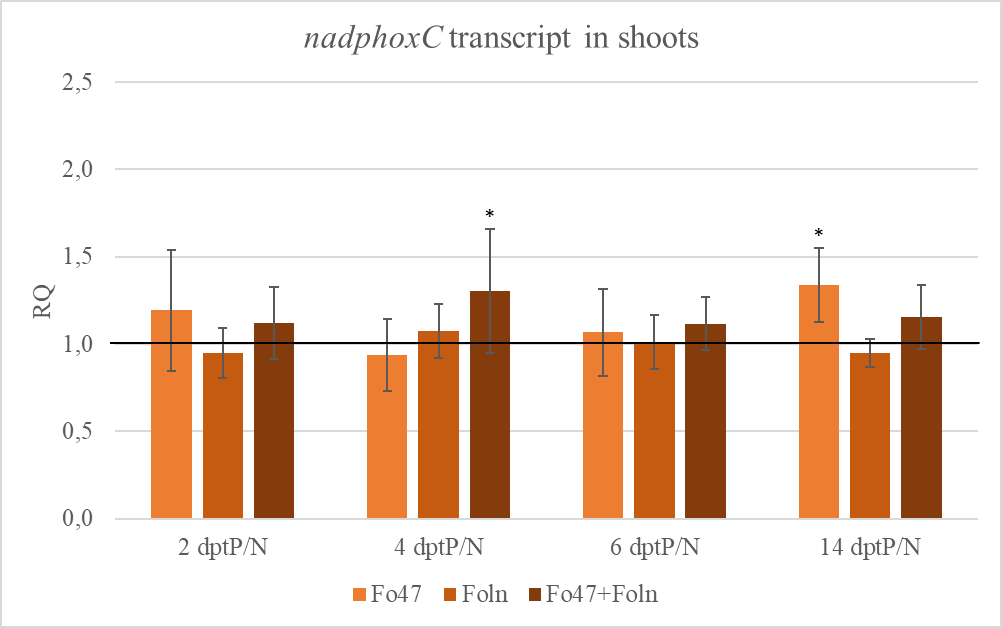

Supplement: Supplementary file 2 — Figure S2: Transcript levels of PR genes and genes involved in ROS metabolism in roots and shoots of plants primed with non‐pathogenic strain Fo47 and plants treated with both strains simultaneously. Changes in transcript levels of PR genes (chitinase and β‐glucanase) and ROS metabolism genes (NADPH oxidase D, NADPH oxidase F, and NADPH oxidase C, three isoforms of superoxide dismutase (sod): sodCu/Zn, sodMn, and sodFe, catalase and ascorbate peroxidase) are shown as relative quantity (RQ) to the reference gene (actin) for control. Results were obtained by real‐time PCR on a cDNA template and are presented as mean ± SD (n = 3). The significance of differences between groups was determined using a one‐way ANOVA, followed by Fisher's post hoc test. Differences were considered statistically significant when p < 0.05 (* for comparison to control, non‐treated plants from the same time point as the sample; ▲for comparison of plants primed with non‐pathogenic strain Fo47 or plants treated with both strains simultaneously with Foln treated plants from the same time point as sample). [file EMI4-18-e70263-s001.docx]

A)


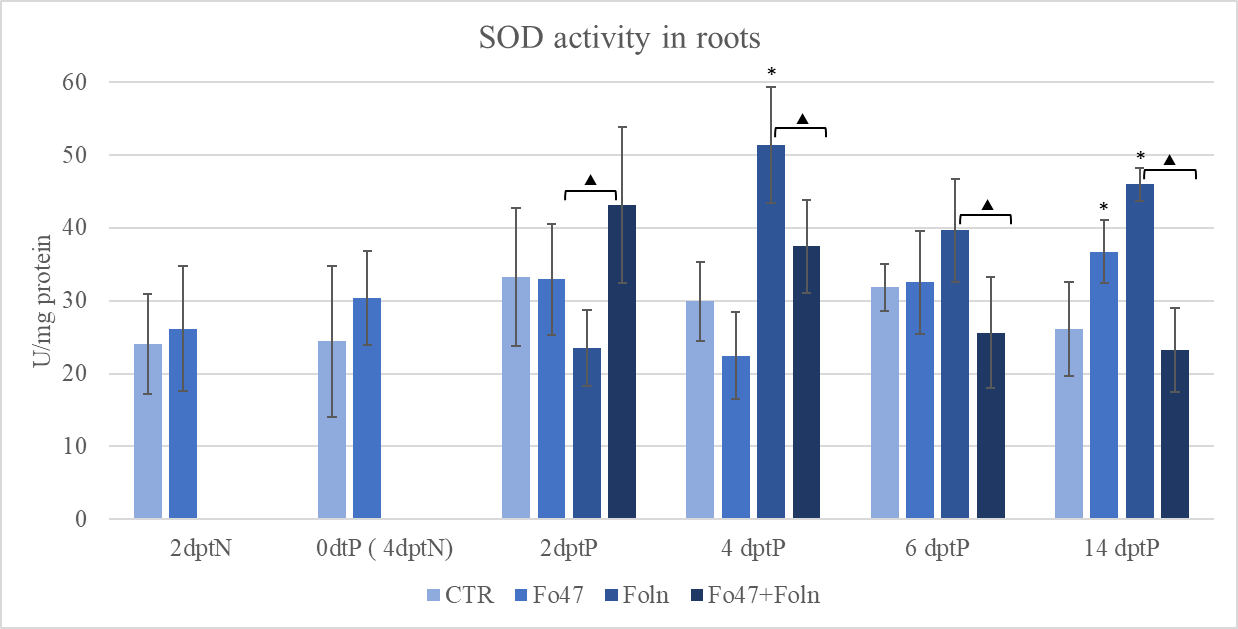

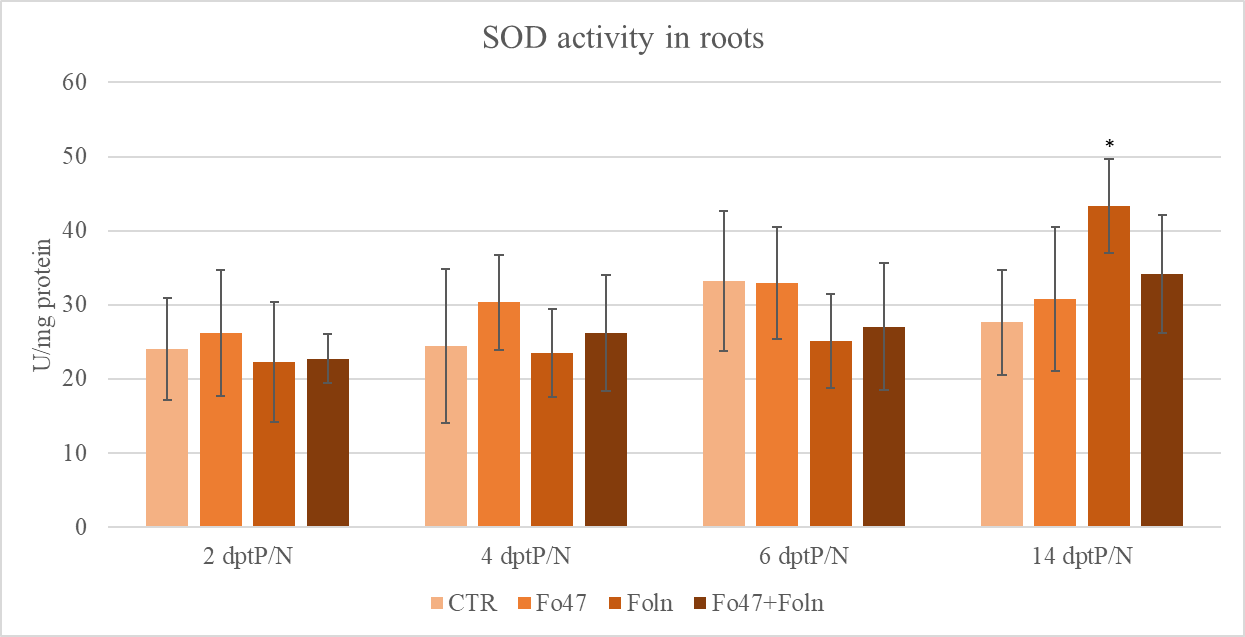


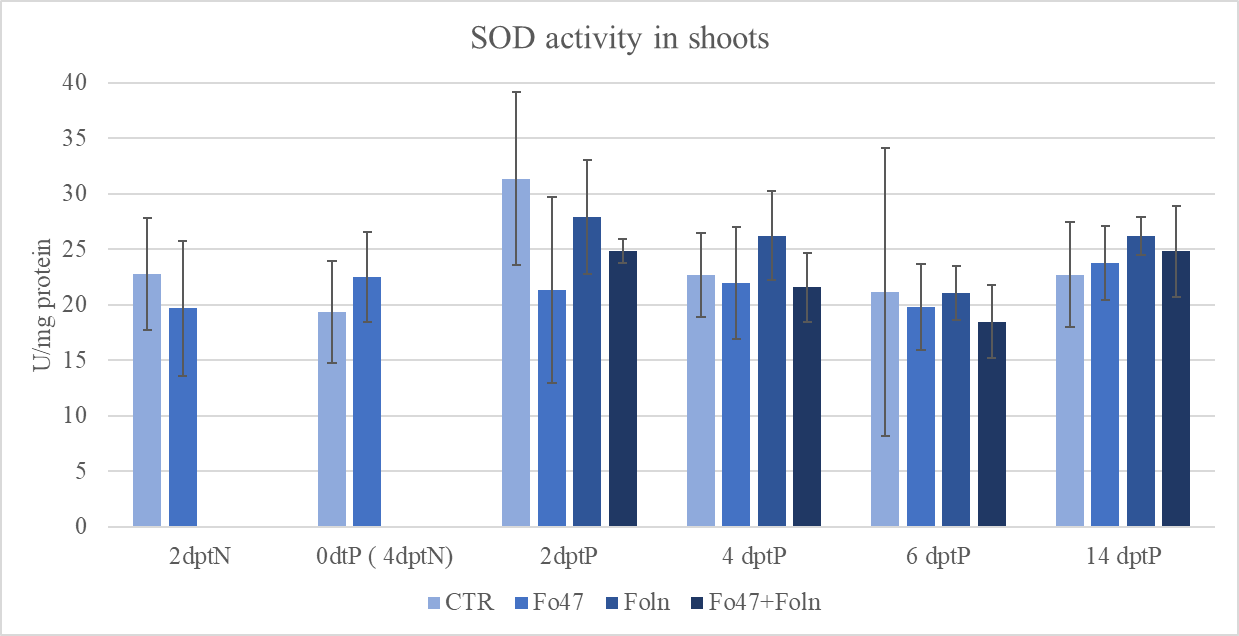

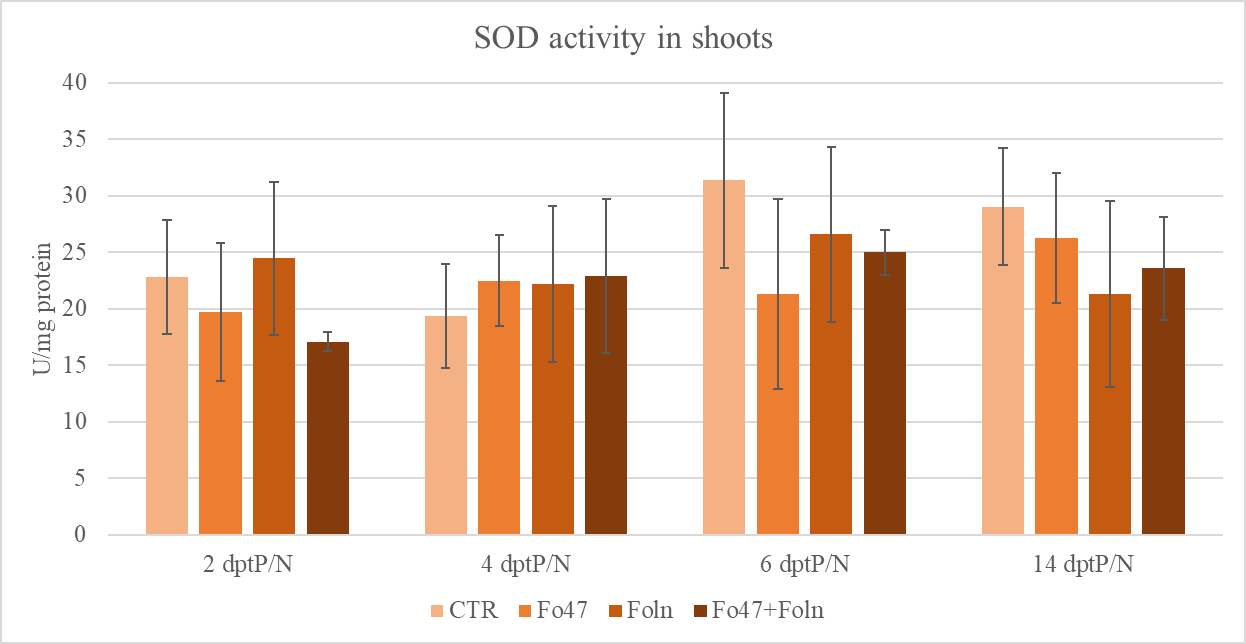


B)


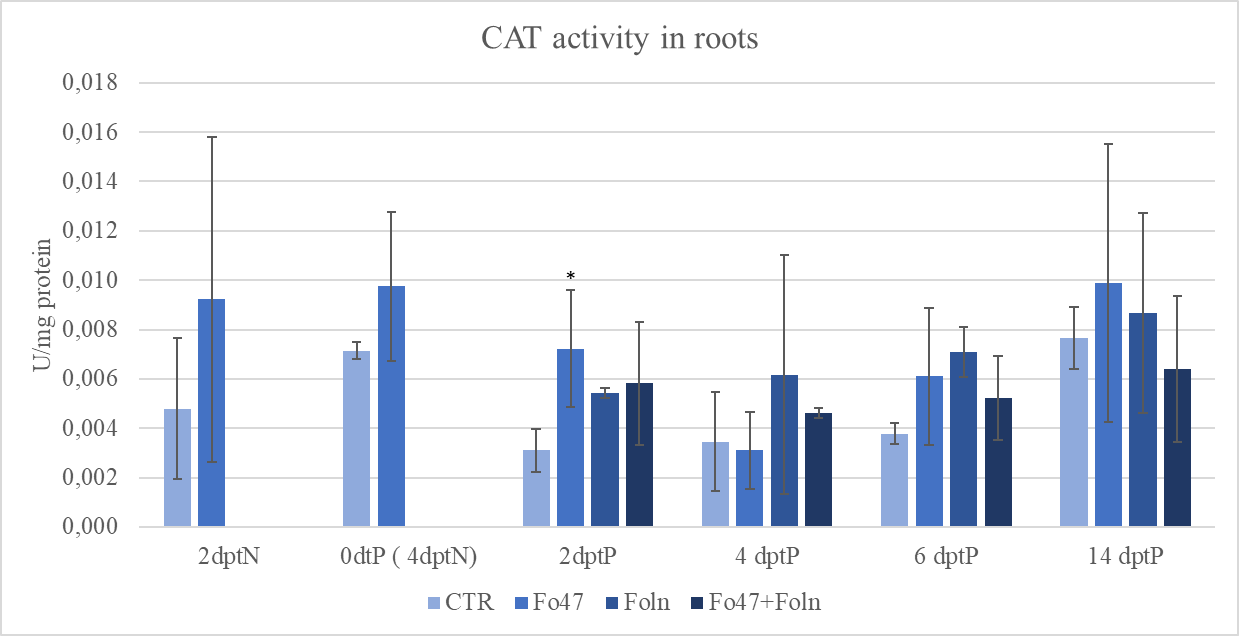

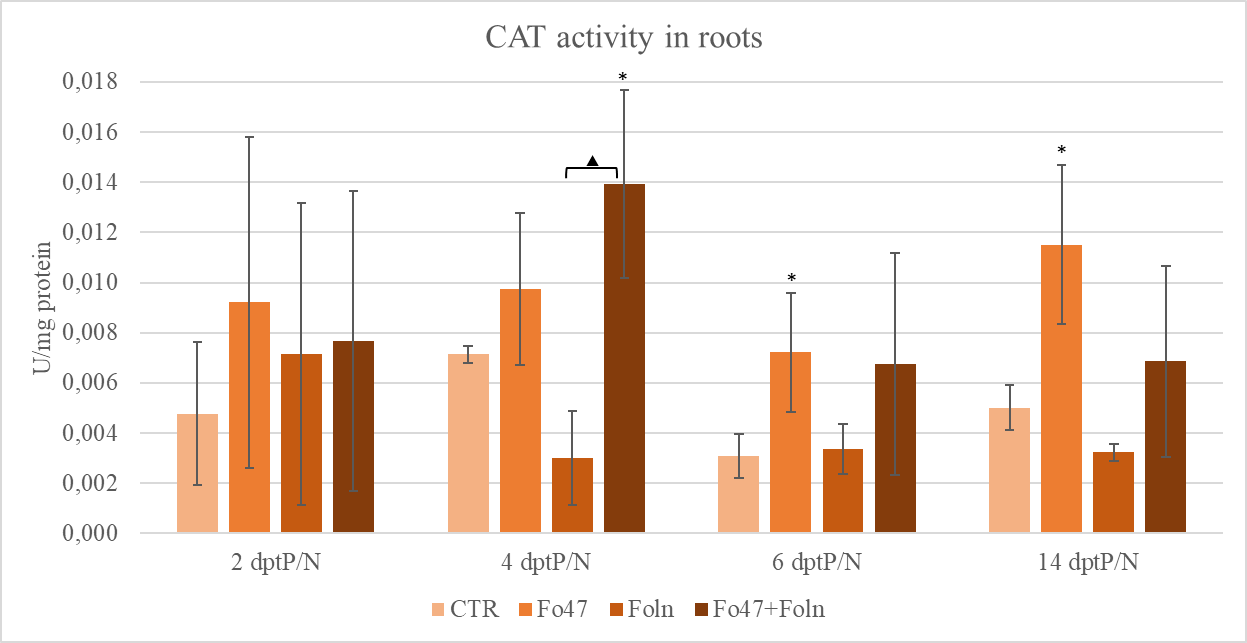


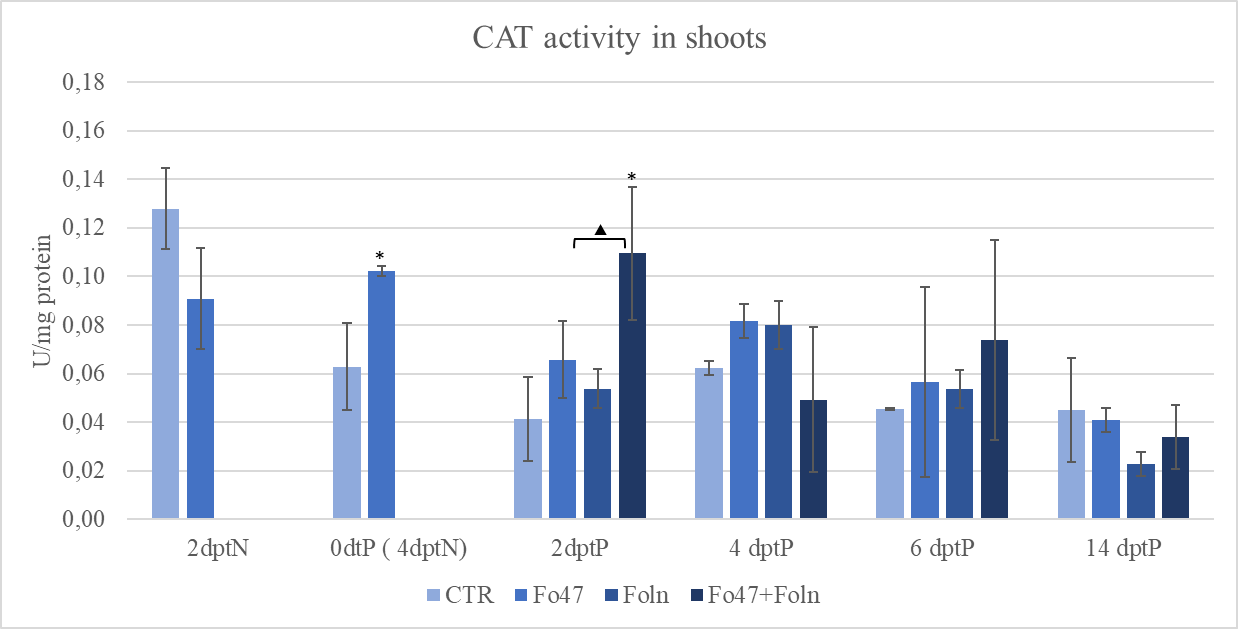

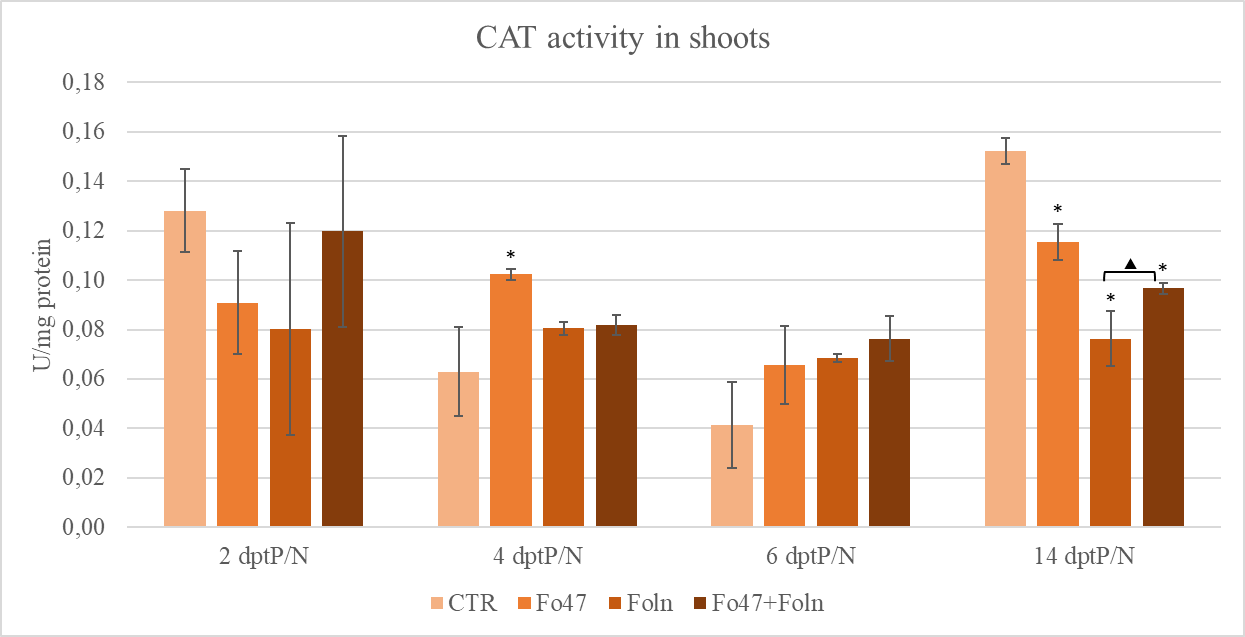


C)


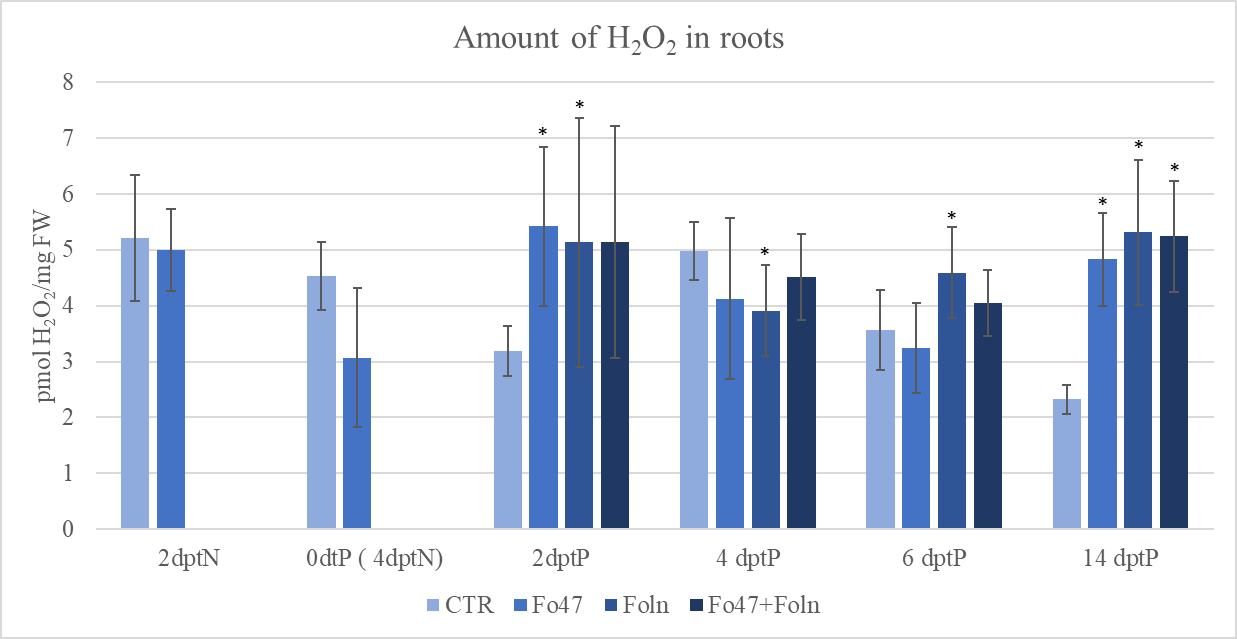

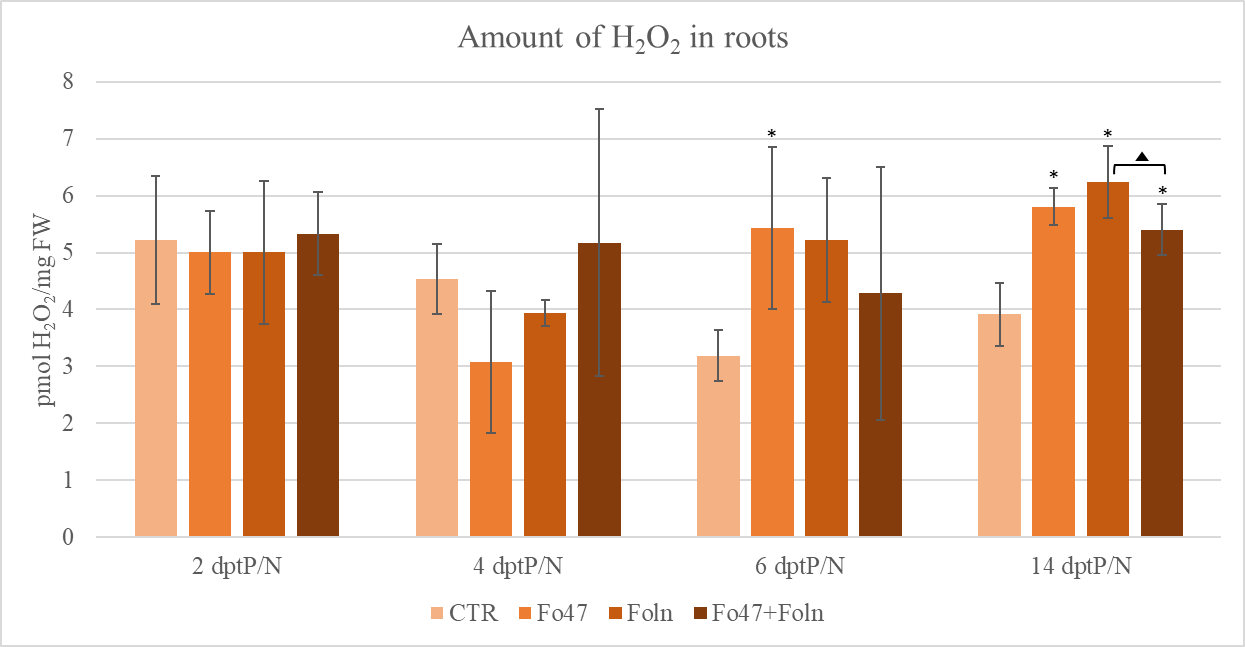

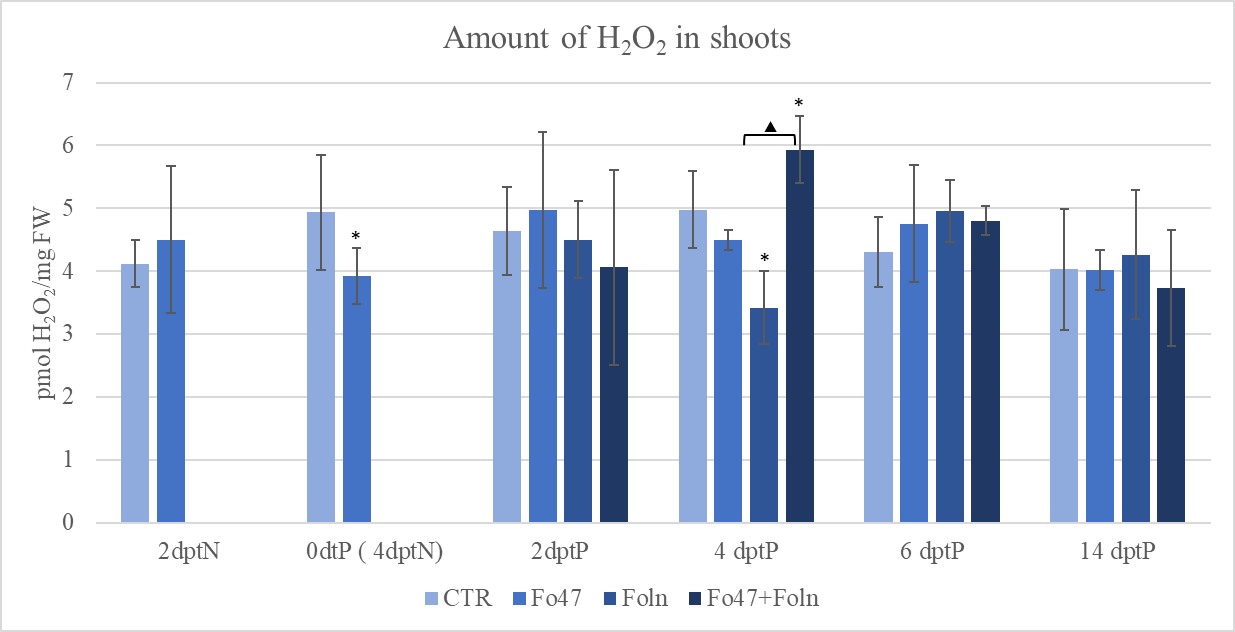

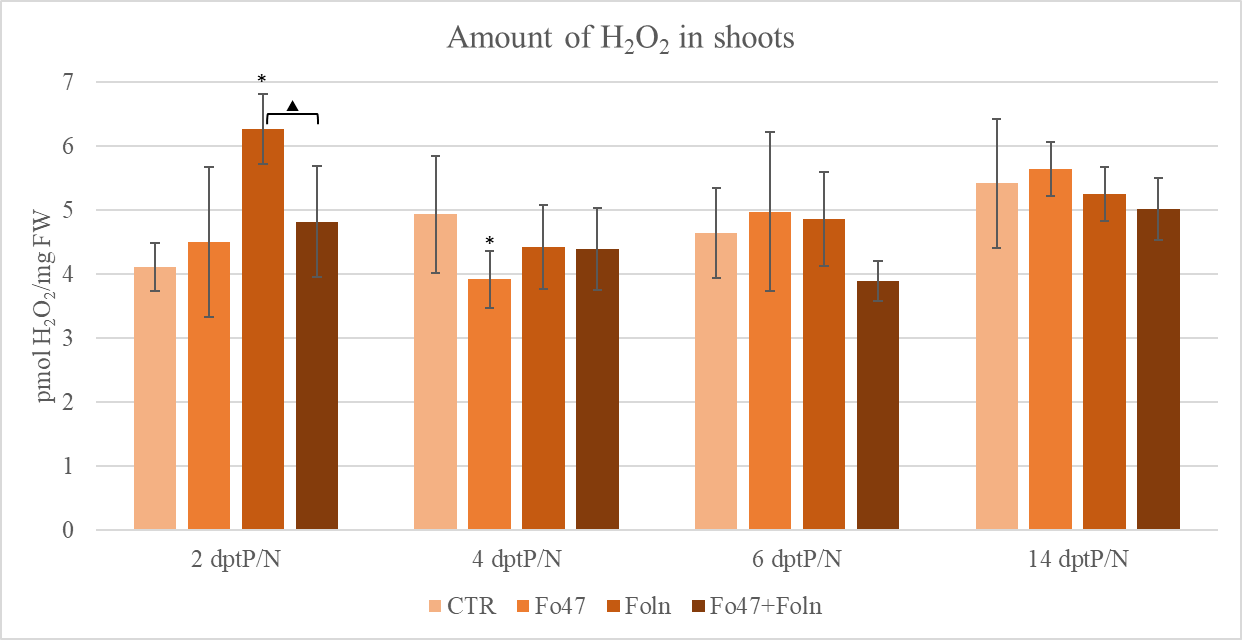


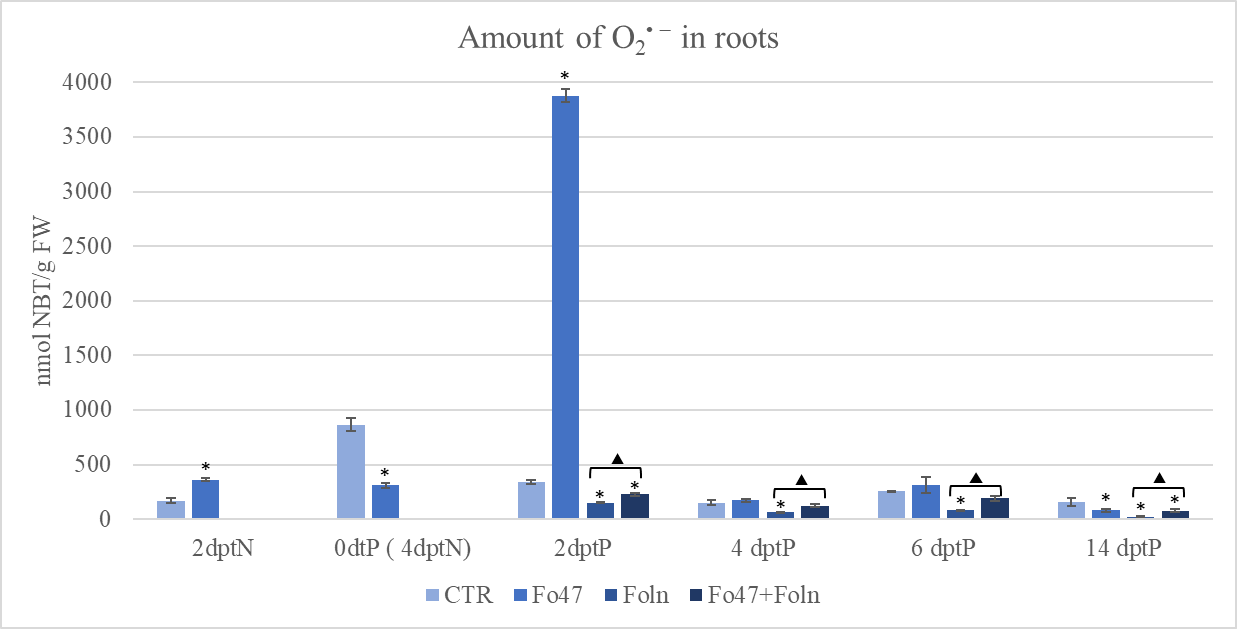

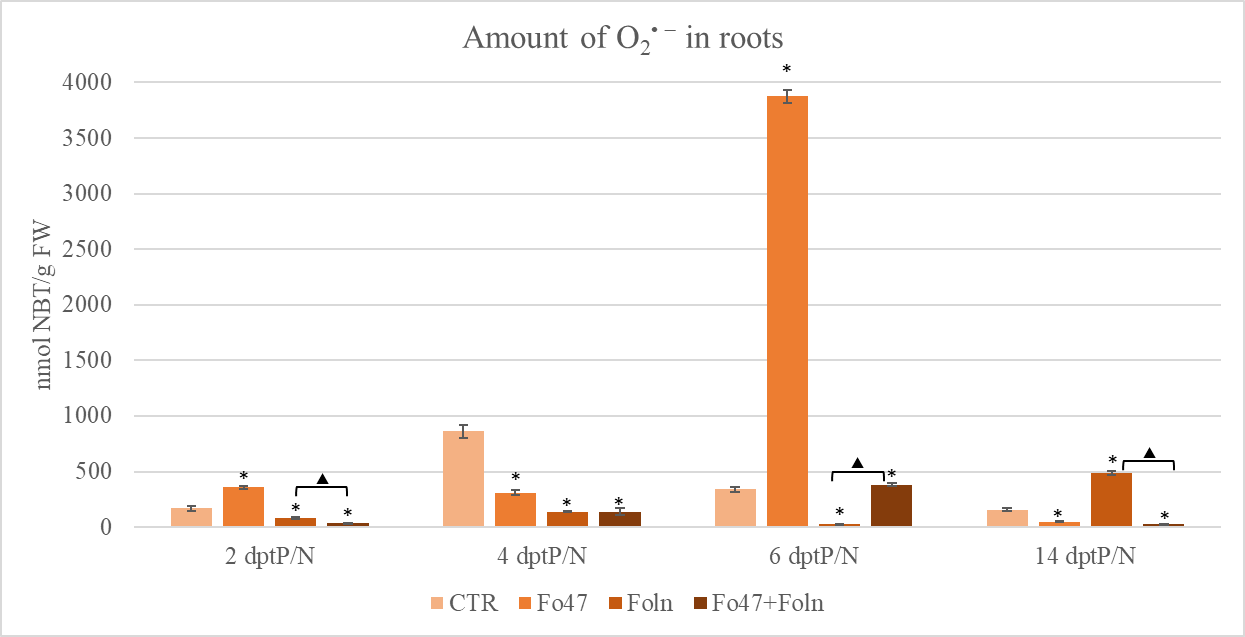

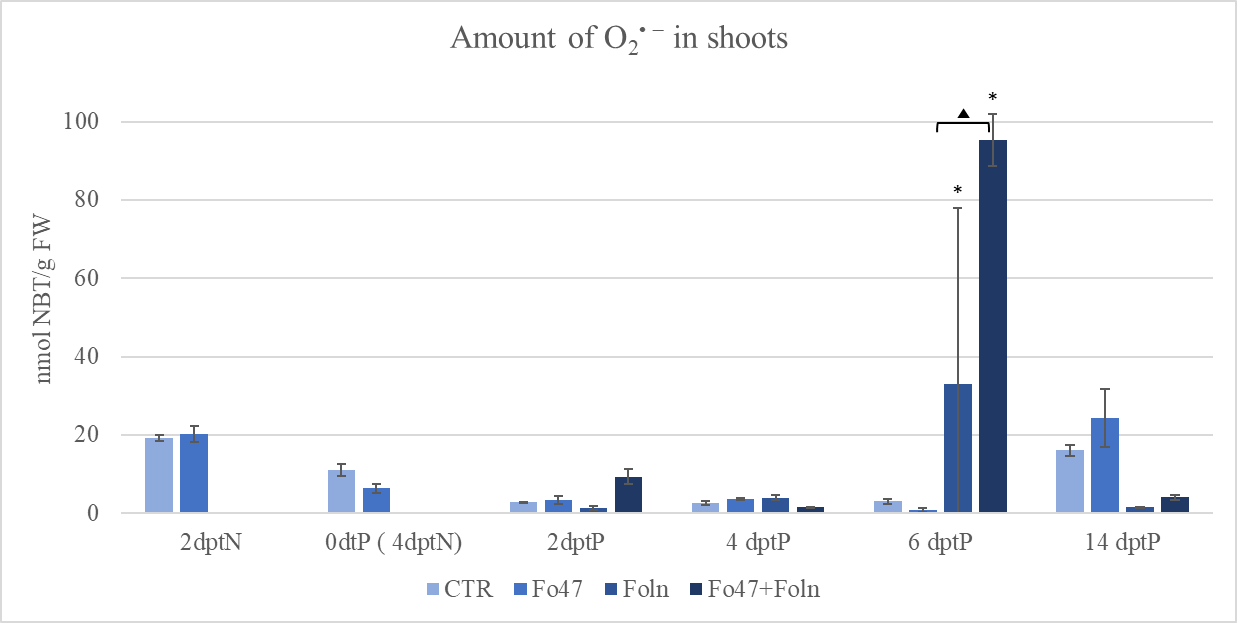

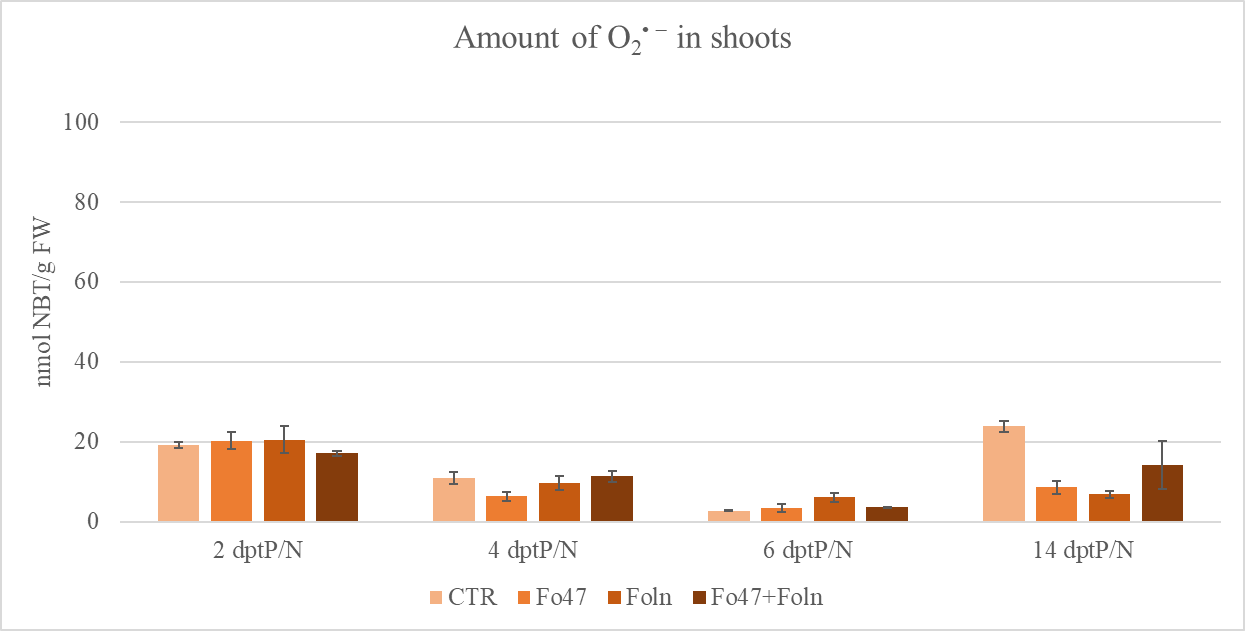

Supplement: Supplementary file 3 — Figure S3: Catalase and superoxide dismutase activity and H2O2 and O2 − content in roots and shoots of plants primed with non‐pathogenic strain Fo47 and plants treated with both strains simultaneously. Bars represent the mean ± SD from three replicates. The significance of differences between groups was determined using a one‐way ANOVA, followed by Fisher's post hoc test. Differences were considered statistically significant when p < 0.05 (* for comparison to control, non‐treated plants from the same time point as the sample; ▲for comparison of plants primed with non‐pathogenic strain Fo47 or plants treated with both strains simultaneously with Foln treated plants from the same time point as sample). [file EMI4-18-e70263-s002.docx]

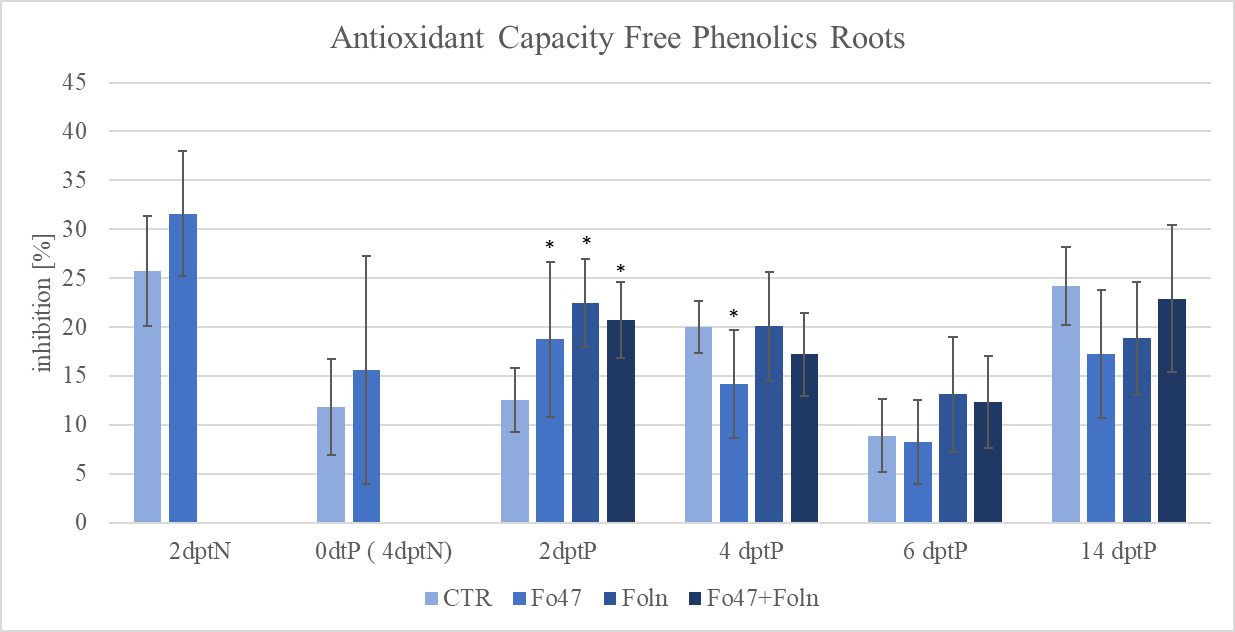

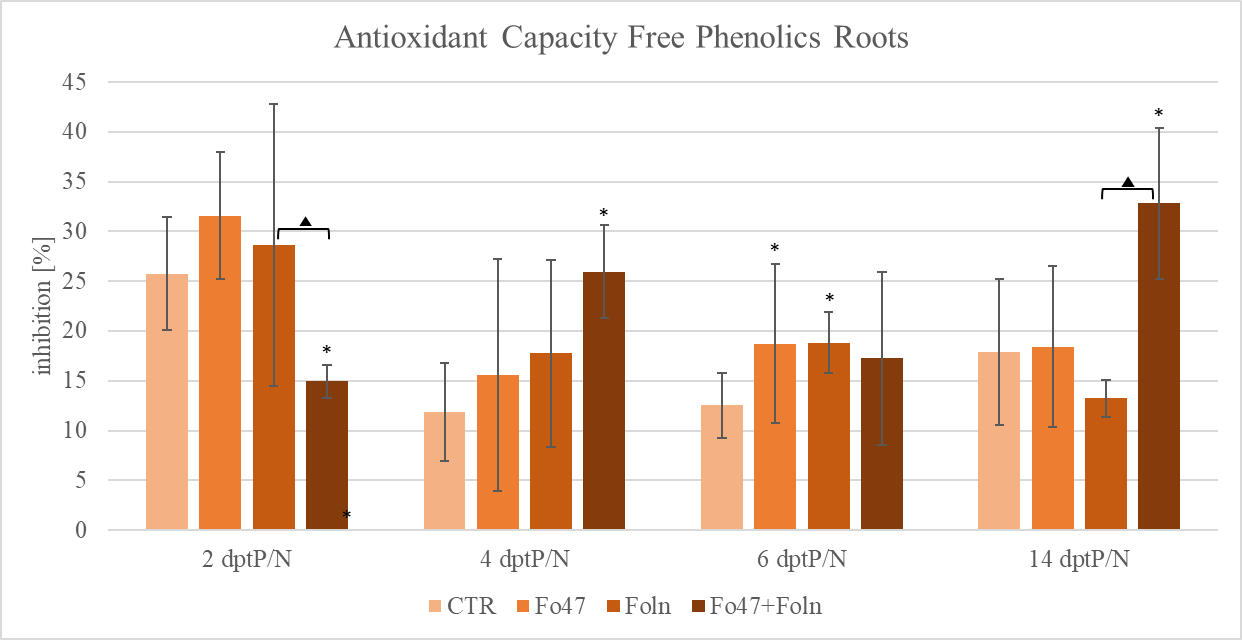


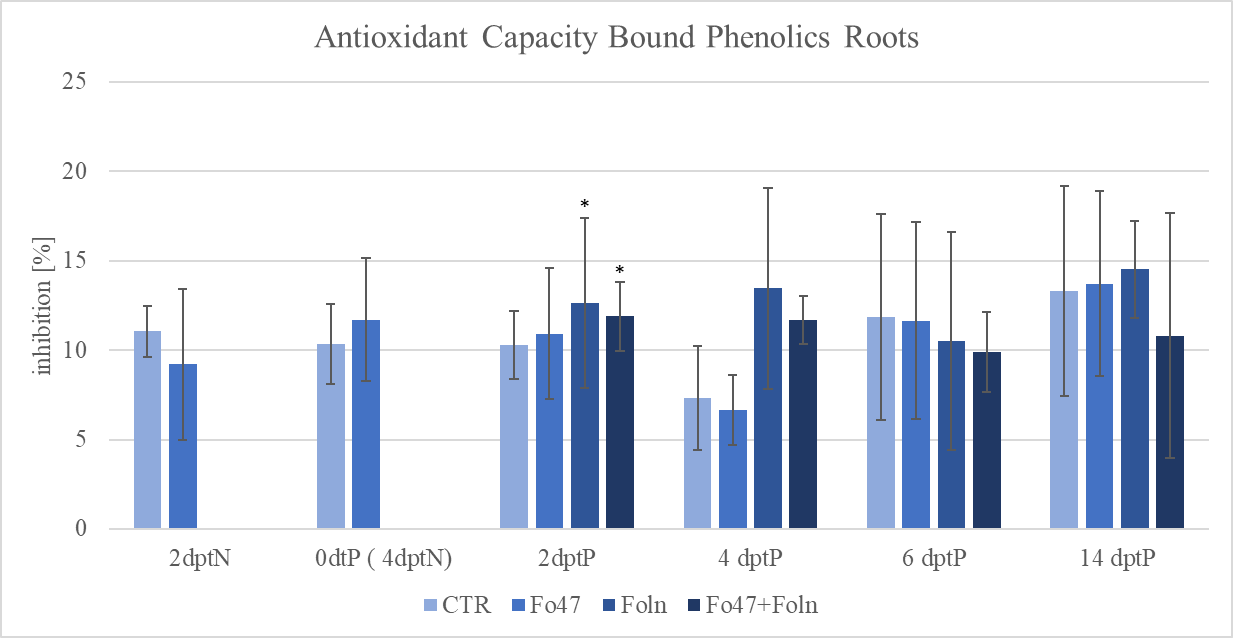

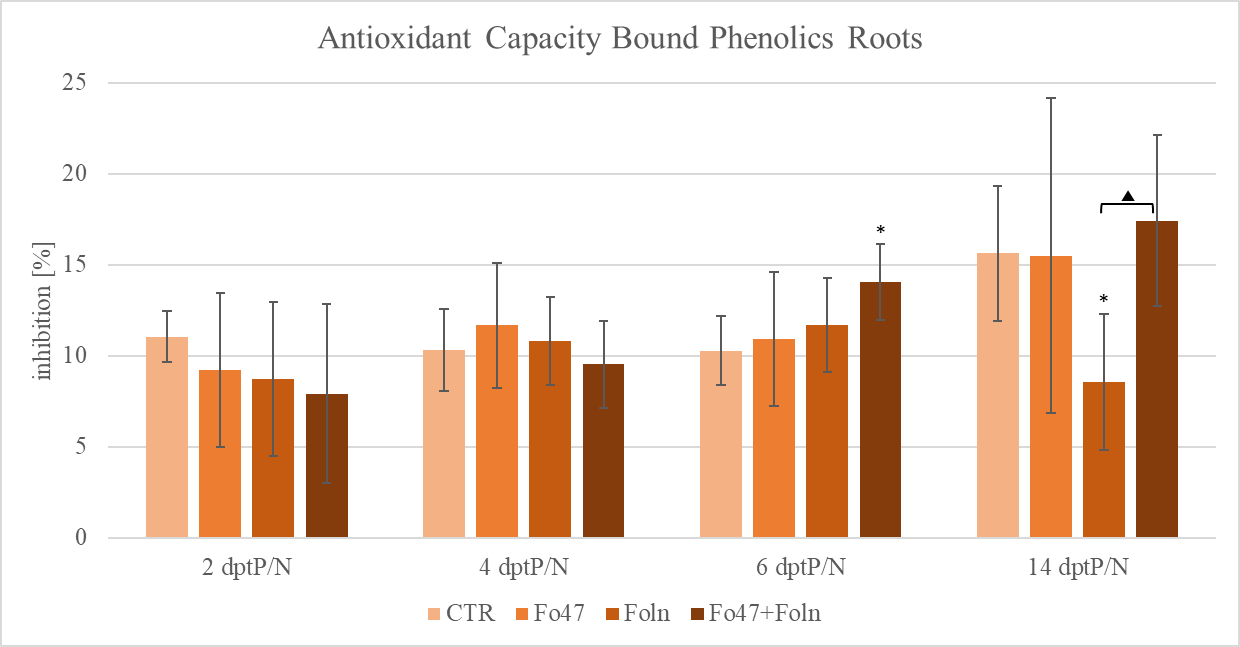


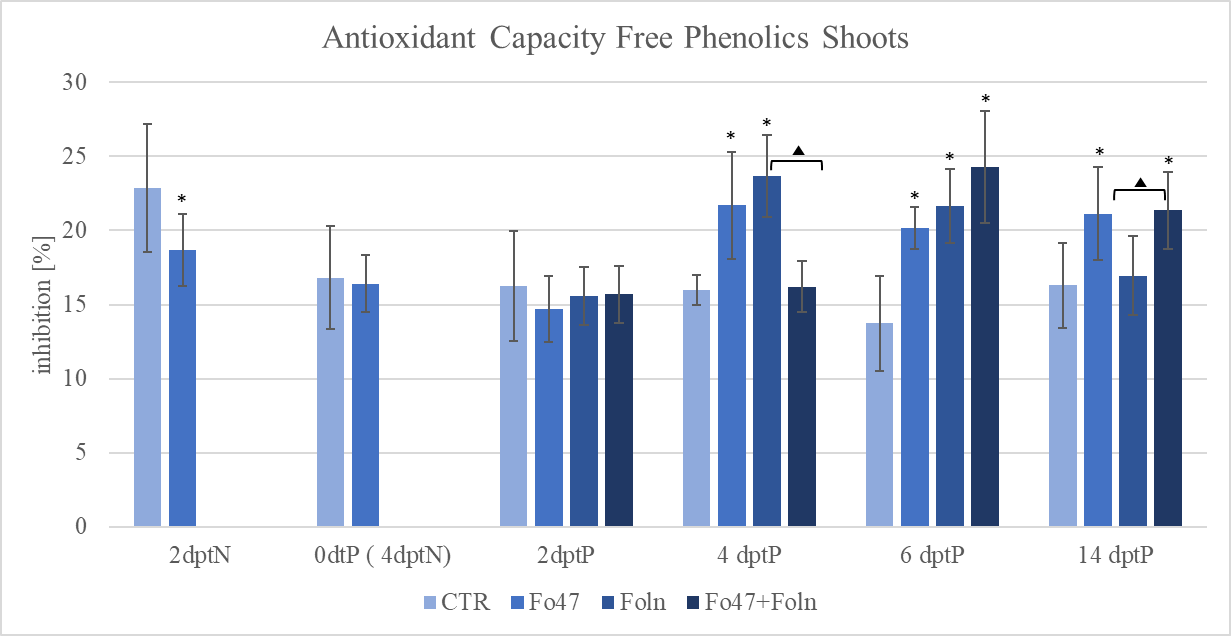

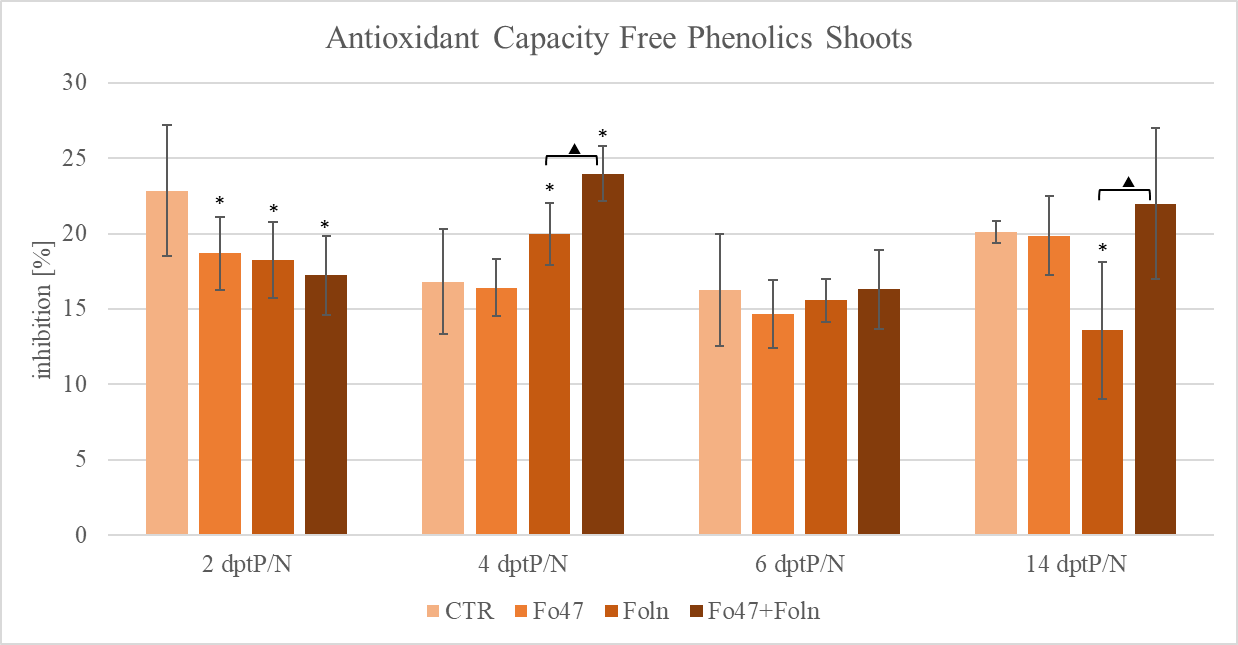


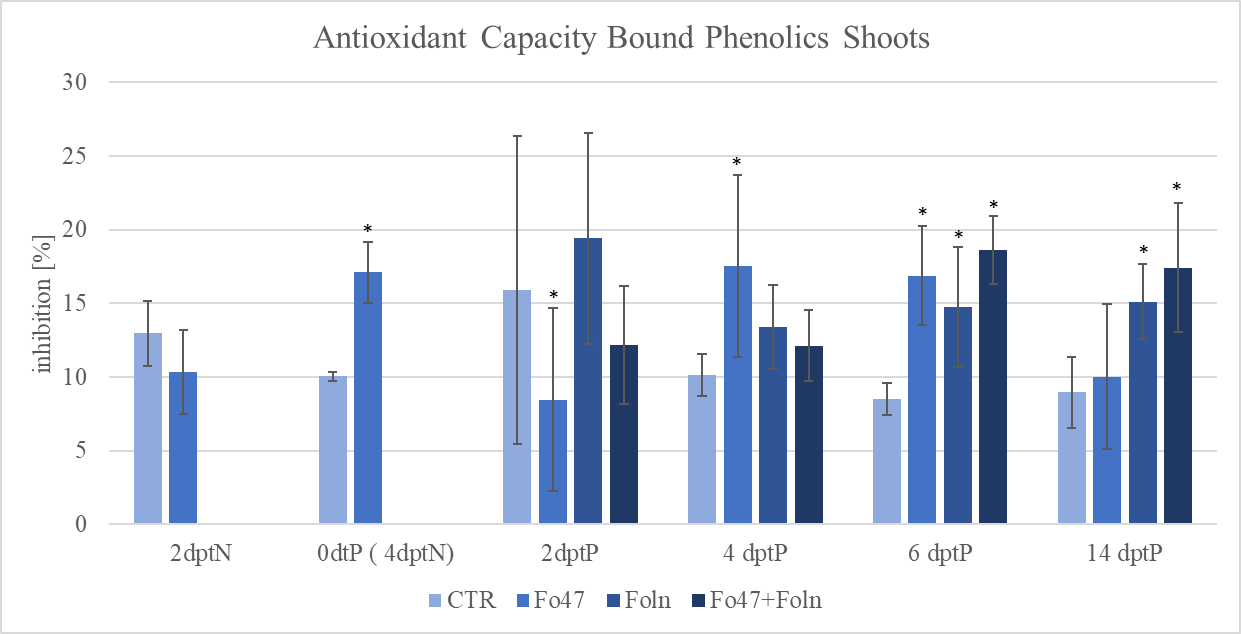

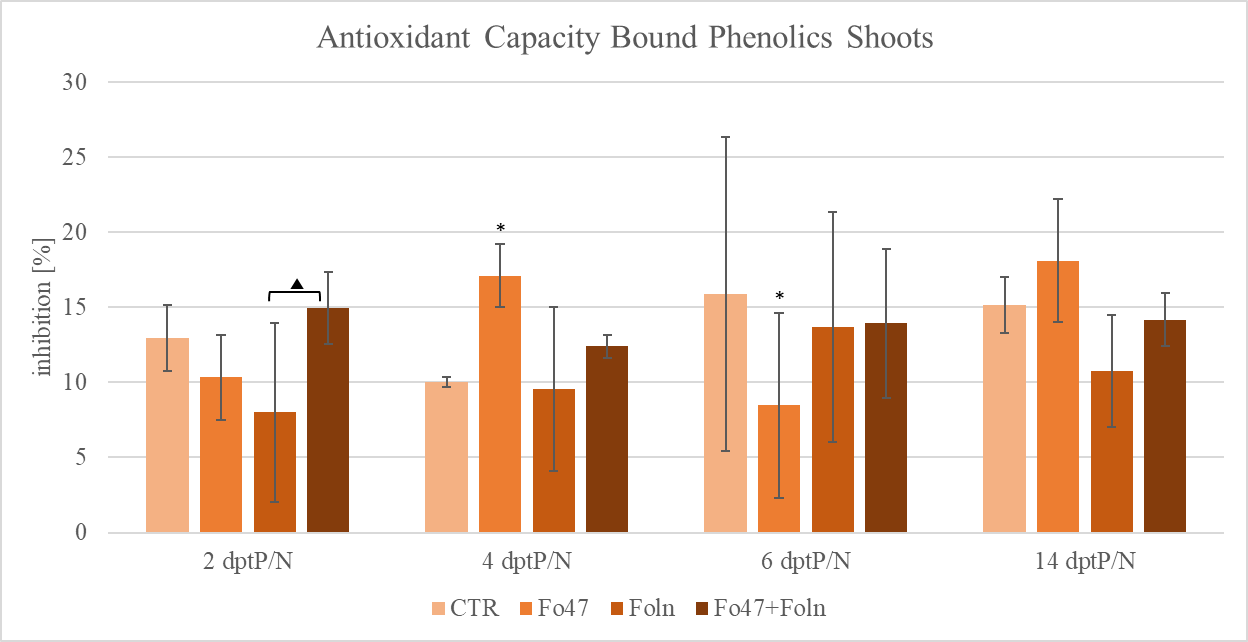

Supplement: Supplementary file 4 — Figure S4: Antioxidant potential in extract including free phenolic compounds and in extract bound phenolic compounds from roots and shoots of plants primed with non‐pathogenic strain of Fusarium oxysporum and plants treated with both strains of Fusarium oxysporum simultaneously. The significance of differences between groups was determined using a one‐way ANOVA, followed by Fisher's post hoc test. Differences were considered statistically significant when p < 0.05 (* for comparison to control, non‐treated plants from the same time point as the sample; ►◄ for comparison of plants primed with non‐pathogenic strain Fo47 or plants treated with both strains simultaneously with Foln treated plants from the same time point as sample). [file EMI4-18-e70263-s004.docx]
