# Supplementary material for: Systemic Activation of the Antioxidant System by Root Priming With Non‐Pathogenic Fusarium oxysporum in Flax Infected With Pathogenic Fusarium oxysporum
Source: Environ Microbiol Rep. 2026 Jan 8;18(1):e70263. doi: 10.1111/1758-2229.70263 (PMC12784107; doi:10.1111/1758-2229.70263)
Supplement: Supplementary file 5 — Table S1: Sequences of primers used for the PCR reaction. [file EMI4-18-e70263-s005.docx]

| **GENE** | **FP** | **RP** |
| --- | --- | --- |
| **actin** | CCGGTGTTATGGTTGGAAT | TGTAGAAAGTGTGATGCCAAA |
| **chitinase** | CATCCAATGAATGGCCTT | GGCTGTTCGGAATGATATCTC |
| **β-glucanase** | CTAGGCAGCGTGAAAGC | CGTCGAAGAGGTTGGTG |
| **genome fragment of *F. oxysporum*** | CCAAGGCAGAAGTCGATGTA | AGGTCGTTGGTGAGAAAG |
| **fungal murein transglycosylase** | TCTCAACGGTGTCGAGTCTAA | CACCCTGGTTGCAGATAAT |
| **CuSOD** | CCACTGTAACTGGAAACATCT | GGTGTTCATCCTCAGGAGCA |
| **MnSOD** | CTGAAGGTGCTGCACTC | CATGCTCCCAGACATCAAT |
| **FeSOD** | ACCATGAATTCTTCTGGGAATC | CACCCGGAACCAAACTG |
| **catalase** | TGCAATCGTGGTTCCTGGTGTTTA | GGTGATTGTTGTGATGAGCACACT |
| **apx** | GGTGGTCACACTTTGGG | GAAGAGCCTTGTCACTCG |
| **NADPH oxidase D** | CATCACTTCTGCTCCCG | TTGTTGTTTCCCTGAAATCCAT |
| **NADPH oxidase F** | GGAAATGTGCTCACGTTG | GAACGCTAAGGTAGTCATCC |
| **NADPH oxidase C** | CATGATAGAGAACCTAGAAATGC | TACTTTGTGTTTCTTAGCCATCTC |

Table S1. Sequences of primers used for the PCR reaction.
