# Supplementary material for: Systemic Activation of the Antioxidant System by Root Priming With Non‐Pathogenic Fusarium oxysporum in Flax Infected With Pathogenic Fusarium oxysporum
Source: Environ Microbiol Rep. 2026 Jan 8;18(1):e70263. doi: 10.1111/1758-2229.70263 (PMC12784107; doi:10.1111/1758-2229.70263)
Supplement: Supplementary file 6 — Table S2: Phenolic compound content (vanillin, trans‐ferulic acid and p‐coumaric acid) and antioxidant potential (in extract including free phenolic compounds and in extract bound phenolic compounds) from roots and shoots of plants primed with non‐pathogenic strain of Fusarium oxysporum and plants treated with both strains of Fusarium oxysporum simultaneously. The significance of differences between groups was determined using a one‐way ANOVA, followed by Fisher's post hoc test. Differences were considered statistically significant when p < 0.05 (* for comparison to control, non‐treated plants from the same time point as the sample; ►◄ for comparison of plants primed with non‐pathogenic strain Fo47 or plants treated with both strains simultaneously with Foln treated plants from the same time point as sample). [file EMI4-18-e70263-s006.docx]

| Phenolic compounds in roots of plants primed with Fo47 | | | | | | | |
| --- | --- | --- | --- | --- | --- | --- | --- |
| [ug/g FW] | | vanillin | | trans-ferulic acid | | *p*-coumaric acid | |
|  |  | Mean | ± SD | Mean | ± SD | Mean | ± SD |
| 2 dptN | ctr | 0.45 | 0.15 | 11.08 | 0.57 | 2.06 | 0.10 |
|  | Fo47 | 1.04 | 0.50 | 7.75 | 0.10 | 1.89 | 0.14 |
| 4 dptN  (0 dptP) | ctr | 0.71 | 0.04 | 13.06 | 1.36 | 2.94 | 0.61 |
|  | Fo47 | 1.43 | 0.63 | 10.93 | 0.39 | 2.36 | 0.05 |
| 2 dptP  (6 dptN) | ctr | 1.31 | 0.29 | 10.60 | 0.91 | 2.31 | 0.21 |
|  | Fo47 | 0.91 | 0.11 | 10.74 | 0.64 | 1.97 | 0.17 |
|  | Fol | 2.96 | 2.11 | 12.94 | 1.93 | 2.66 | 0.27 |
|  | Fo47+Fol | 0.42 | 0.02 | 10.54 | 0.67 | 2.34 | 0.36 |
| 4 dptP  (8 dptN) | ctr | 2.83 | 0.85 | 11.46 | 1.55 | 2.87 | 0.44 |
|  | Fo47 | 2.40 | 0.09 | 11.15 | 0.29 | 2.41 | 0.25 |
|  | Fol | 3.07 | 2.21 | 11.05 | 0.43 | 1.79 | 0.03 |
|  | Fo47+Fol | 2.63 | 0.91 | 7.85 | 3.02 | 1.96 | 0.39 |
| 6 dptP  (10 dptN) | ctr | 1.95 | 0.00 | 5.84 | 5.44 | 1.30 | 1.03 |
|  | Fo47 | 2.12 | 1.72 | 10.60 | 2.57 | 2.29 | 0.52 |
|  | Fol | 0.52 | 0.27 | 10.67 | 1.22 | 2.47 | 0.37 |
|  | Fo47+Fol | 2.91 | 0.95 | 9.76 | 1.03 | 2.23 | 0.45 |
| 14 dptP  (18 dptN) | ctr | 3.53 | 2.36 | 11.72 | 0.17 | 2.24 | 0.00 |
|  | Fo47 | 5.22 | 0.00 | 12.77 | 0.37 | 2.25 | 0.00 |
|  | Fol | 1.69 | 0.73 | 13.13 | 2.70 | 3.26 | 0.39 |
|  | Fo47+Fol | 2.02 | 0.55 | 10.82 | 0.85 | 2.21 | 0.15 |

| Phenolic compounds in roots of plants treated with both strains simultaneously | | | | | | | |
| --- | --- | --- | --- | --- | --- | --- | --- |
| [ug/g FW] | | vanillin | | trans-ferulic acid | | *p*-coumaric acid | |
|  |  | Mean | ± SD | Mean | ± SD | Mean | ± SD |
| 2 dptP/N | ctr | 0.45 | 0.15 | 11.08 | 0.57 | 2.06 | 0.10 |
|  | Fo47 | 1.04 | 0.50 | 7.75 | 0.10 | 1.89 | 0.14 |
|  | Fol | 1.51 | 0.19 | 11.15 | 0.00 | 0.15 | 0.01 |
|  | Fo47+Fol | 2.79 | 1.57 | 8.08 | 0.35 | 1.52 | 0.13 |
| 4 dptP/N | ctr | 0.71 | 0.04 | 13.06 | 1.36 | 2.94 | 0.61 |
|  | Fo47 | 1.43 | 0.63 | 10.93 | 0.39 | 2.36 | 0.05 |
|  | Fol | 2.43 | 1.96 | 10.73 | 1.58 | 2.09 | 0.27 |
|  | Fo47+Fol | 0.33 | 0.09 | 9.12 | 0.81 | 1.87 | 0.21 |
| 6 dptP/N | ctr | 1.31 | 0.29 | 10.60 | 0.91 | 2.31 | 0.21 |
|  | Fo47 | 0.91 | 0.11 | 10.74 | 0.64 | 1.97 | 0.17 |
|  | Fol | 2.55 | 1.38 | 12.26 | 0.44 | 3.02 | 0.28 |
|  | Fo47+Fol | 2.71 | 1.34 | 12.09 | 0.17 | 2.44 | 0.18 |
| 14 dptP/N | ctr | 2.96 | 2.52 | 14.09 | 1.30 | 2.83 | 0.31 |
|  | Fo47 | 5.49 | 0.52 | 14.24 | 0.96 | 2.90 | 0.28 |
|  | Fol | 1.63 | 0.19 | 11.17 | 2.13 | 2.58 | 0.58 |
|  | Fo47+Fol | 3.45 | 2.29 | 15.62 | 0.62 | 3.68 | 0.19 |

| Phenolic compounds in shoots of plants primed with Fo47 | | | | | | | |
| --- | --- | --- | --- | --- | --- | --- | --- |
| [ug/g FW] | | vanillin | | trans-ferulic acid | | *p*-coumaric acid | |
|  |  | Mean | ± SD | Mean | ± SD | Mean | ± SD |
| 2dptN | ctr | 0.97 | 0.37 | 25.94 | 5.81 | 21.21 | 3.18 |
|  | Fo47 | 0.58 | 0.05 | 26.84 | 2.49 | 21.77 | 1.85 |
| 4 dptN  (0 dptP) | ctr | 0.97 | 0.48 | 23.03 | 3.03 | 20.12 | 0.06 |
|  | Fo47 | 1.56 | 0.04 | 32.57 | 1.26 | 24.96 | 0.09 |
| 2 dptP  (6 dptN) | ctr | 0.51 | 0.06 | 29.48 | 1.88 | 20.54 | 1.74 |
|  | Fo47 | 0.90 | 0.36 | 27.55 | 0.50 | 19.09 | 1.35 |
|  | Fol | 0.47 | 0.10 | 30.01 | 2.54 | 20.47 | 1.53 |
|  | Fo47+Fol | 0.51 | 0.14 | 27.21 | 0.26 | 18.14 | 0.44 |
| 4 dptP  (8 dptN) | ctr | 0.67 | 0.01 | 32.73 | 0.89 | 22.20 | 1.72 |
|  | Fo47 | 1.78 | 0.02 | 30.48 | 2.19 | 20.91 | 1.63 |
|  | Fol | 1.38 | 0.25 | 33.22 | 0.15 | 23.26 | 0.65 |
|  | Fo47+Fol | 1.69 | 0.01 | 29.61 | 3.07 | 21.43 | 2.46 |
| 6 dptP  (10 dptN) | ctr | 1.73 | 0.42 | 33.37 | 3.20 | 21.70 | 2.85 |
|  | Fo47 | 1.11 | 0.00 | 32.29 | 6.46 | 20.05 | 3.01 |
|  | Fol | 1.94 | 0.14 | 36.33 | 3.88 | 23.75 | 1.13 |
|  | Fo47+Fol | 0.69 | 0.12 | 29.15 | 0.49 | 17.39 | 0.80 |
| 14 dptP  (18 dptN) | ctr | 2.32 | 0.12 | 35.86 | 2.58 | 21.26 | 0.03 |
|  | Fo47 | 0.97 | 0.01 | 31.79 | 2.67 | 17.41 | 2.54 |
|  | Fol | 1.17 | 0.10 | 37.06 | 5.95 | 19.21 | 3.15 |
|  | Fo47+Fol | 2.63 | 0.33 | 36.09 | 1.10 | 19.25 | 0.29 |

| Phenolic compounds in shoots of plants treated with both strains simultaneously | | | | | | | |
| --- | --- | --- | --- | --- | --- | --- | --- |
| [ug/g FW] | | vanillin | | trans-ferulic acid | | *p*-coumaric acid | |
|  |  | Mean | ± SD | Mean | ± SD | Mean | ± SD |
| 2dptP/N | ctr | 0.97 | 0.37 | 25.94 | 5.81 | 21.21 | 3.18 |
|  | Fo47 | 0.58 | 0.05 | 26.84 | 2.49 | 21.77 | 1.85 |
|  | Fol | 0.39 | 0.08 | 21.26 | 1.72 | 21.41 | 0.38 |
|  | Fo47+Fol | 1.22 | 0.05 | 31.05 | 1.63 | 24.22 | 1.74 |
| 4 dptP/N | ctr | 0.97 | 0.48 | 23.03 | 3.03 | 20.12 | 0.06 |
|  | Fo47 | 1.56 | 0.04 | 32.57 | 1.26 | 24.96 | 0.09 |
|  | Fol | 0.75 | 0.25 | 26.07 | 0.81 | 21.09 | 0.60 |
|  | Fo47+Fol | 0.47 | 0.03 | 26.30 | 1.87 | 19.59 | 0.94 |
| 6 dptP/N | ctr | 0.51 | 0.06 | 29.48 | 1.88 | 20.54 | 1.74 |
|  | Fo47 | 0.90 | 0.36 | 27.55 | 0.50 | 19.09 | 1.35 |
|  | Fol | 0.88 | 0.12 | 30.79 | 0.90 | 20.56 | 0.11 |
|  | Fo47+Fol | 1.73 | 0.08 | 30.64 | 0.18 | 20.89 | 1.13 |
| 14 dptP/N | ctr | 2.36 | 0.24 | 31.49 | 1.94 | 21.45 | 3.97 |
|  | Fo47 | 1.84 | 0.76 | 29.28 | 2.83 | 17.11 | 2.10 |
|  | Fol | 2.21 | 0.23 | 34.18 | 2.73 | 19.61 | 1.90 |
|  | Fo47+Fol | 1.92 | 0.94 | 37.52 | 1.81 | 22.08 | 1.32 |

Table S2. Phenolic compound content (vanillin, trans-ferulic acid and *p*-coumaric acid) and antioxidant potential (in extract including free phenolic compounds and in extract bound phenolic compounds) from roots and shoots of plants primed with non-pathogenic strain of *Fusarium oxysporum* and plants treated with both strains of *Fusarium oxysporum* simultaneously. The significance of differences between groups was determined using a one-way ANOVA was performed, followed by Fisher’s post hoc test. Differences were considered statistically significant when P < 0.05 (* for comparison to control, non-treated plants from the same time point as the sample; ►◄ for comparison of plants primed with non-pathogenic strain Fo47 or plants treated with both strains simultaneously with Foln treated plants from the same time point as sample).
